# Native Capillary Electrophoresis–Mass Spectrometry of Near 1 MDa Non‐Covalent GroEL/GroES/Substrate Protein Complexes

**DOI:** 10.1002/advs.202306824

**Published:** 2024-01-08

**Authors:** Anne‐Lise Marie, Florian Georgescauld, Kendall R. Johnson, Somak Ray, John R. Engen, Alexander R. Ivanov

**Affiliations:** ^1^ Barnett Institute of Chemical and Biological Analysis Department of Chemistry and Chemical Biology Northeastern University 360 Huntington Avenue Boston MA 02115 USA

**Keywords:** ATP‐induced conformational rearrangement, chaperones, GroEL, GroES, native capillary electrophoresis–mass spectrometry

## Abstract

Protein complexes are essential for proteins' folding and biological function. Currently, native analysis of large multimeric protein complexes remains challenging. Structural biology techniques are time‐consuming and often cannot monitor the proteins' dynamics in solution. Here, a capillary electrophoresis‐mass spectrometry (CE–MS) method is reported to characterize, under near‐physiological conditions, the conformational rearrangements of ∽1 MDa GroEL upon complexation with binding partners involved in a protein folding cycle. The developed CE–MS method is fast (30 min per run), highly sensitive (low‐amol level), and requires ∽10 000‐fold fewer samples compared to biochemical/biophysical techniques. The method successfully separates GroEL_14_ (∽800 kDa), GroEL_7_ (∽400 kDa), GroES_7_ (∽73 kDa), and NanA_4_ (∽130 kDa) oligomers. The non‐covalent binding of natural substrate proteins with GroEL_14_ can be detected and quantified. The technique allows monitoring of GroEL_14_ conformational changes upon complexation with (ATPγS)_4–14_ and GroES_7_ (∽876 kDa). Native CE‐pseudo‐MS^3^ analyses of wild‐type (WT) GroEL and two GroEL mutants result in up to 60% sequence coverage and highlight subtle structural differences between WT and mutated GroEL. The presented results demonstrate the superior CE–MS performance for multimeric complexes' characterization versus direct infusion ESI–MS. This study shows the CE–MS potential to provide information on binding stoichiometry and kinetics for various protein complexes.

## Graphical abstract

High‐sensitivity capillary electrophoresis‐mass spectrometry (CE–MS) method to quickly characterize ≈1 MDa GroEL_14_ multimeric protein assembly under near‐physiological conditions is presented. The CE–MS method enables assessment of non‐covalent binding of substrate proteins to GroEL_14_ chaperon and its conformational changes upon complexation with nucleotides and GroES_7_ involved in a protein folding cycle, and in‐depth structural characterization of wild‐type and mutant GroEL_14_ complexes.

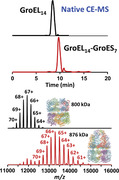

## 1 Introduction

**Figure 1:**
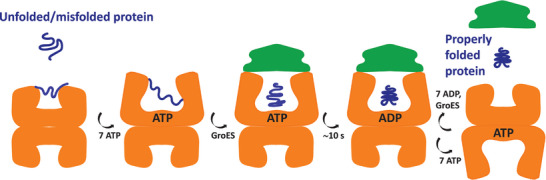
Schematic representation of GroEL‐assisted protein folding cycle. The functional protein folding cycle involves the cooperative binding of ATP and GroES to GroEL. One unfolded or partially folded substrate protein is encapsulated for ≈10 s in the nano‐cage formed by the chaperonin GroEL–GroES assembly for folding or refolding to occur in isolation.

To become functional, most nascent proteins must rapidly reach a unique 3D structure ensemble termed the native state. This phenomenon of protein folding is spontaneous for the majority of proteins (≈70% of newly synthesized cytosolic bacterial proteins).^[^ ^1^ ^]^ However, a major fraction of proteins (≈30%) that are structurally complex and often contain multiple domains cannot reach the native state spontaneously and require assistance from a family of conserved proteins called chaperones. Chaperones are also known under the name of “heat shock proteins” (Hsp) and are classified according to their molecular mass (e.g., Hsp60, Hsp70, Hsp90).^[^ ^2^, ^3^ ^]^ In *E. coli*, the only essential molecular chaperone system is formed by the chaperonin GroEL, an Hsp60 protein, and a co‐chaperonin GroES.^[^ ^4^ ^]^ The GroEL–GroES complex interacts with ≈250 different cytosolic proteins in *E. coli*, of which ≈80 are “strictly dependent” substrates. Such substrate proteins (SPs) are unable to acquire their native structure spontaneously at 37 °C and may rapidly aggregate in the absence of GroEL. GroEL is a multisubunit protein assembly (≈800 kDa) consisting of two heptameric rings stacked back‐to‐back. Each ring is formed by seven non‐covalently bound identical ≈57 kDa subunits, which assemble to form a central cavity (cage) that can accommodate only one single nascent or misfolded SP of 20–60 kDa at a time (**Figure** **1**). Each GroEL subunit has three domains: apical, intermediate, and equatorial. The apical domain is involved in the substrate binding, the equatorial domain fixes the adenosine triphosphate (ATP) nucleotide, and these two domains are physically linked by the intermediate domain. Seven identical ≈10 kDa subunits of GroES assemble as a dome‐shaped ring to form the “lid” of the GroEL cavity. In the presence of ATP and GroES, one unfolded SP at a time would be encapsulated for ≈10 s in the nano‐cage formed by the GroEL–GroES complex for folding or refolding.^[^ ^4^, ^5^, ^6^, ^7^ ^]^ Importantly, ATP binding and ATP hydrolysis to adenosine diphosphate (ADP) induce drastic conformational rearrangements of the apical and intermediate domains of each GroEL subunit, which results in a twofold increase of the central cage volume. Characterizing these conformational changes *in solution* is technically challenging because they represent intermediate states that are sporadically and intermittently populated.

Many techniques, including X‐ray crystallography, nuclear magnetic resonance (NMR), circular dichroism spectroscopy, Fourier transform infrared spectroscopy, high‐performance size exclusion chromatography (SEC), native polyacrylamide gel electrophoresis, capillary electrophoresis (CE), ion mobility spectrometry (IMS), and mass spectrometry (MS) are available for intact protein analysis. In contrast to spectroscopic techniques, which can only detect an average signal in mixed populations, SEC,^[^ ^8^, ^9^, ^10^ ^]^ IMS,^[^ ^11^, ^12^, ^13^ ^]^ and CE^[^ ^14^, ^15^, ^16^ ^]^ are powerful separation techniques that can differentiate protein oligomers and conformers. In CE, near‐physiological conditions can be used to preserve protein conformations and non‐covalent interactions. CE‐based methods were developed to study the folding, unfolding, or misfolding of proteins.^[^ ^17^, ^18^, ^19^ ^]^ Protein–ligand affinity interactions can also be probed with CE, which enables the measurement of binding stoichiometry and binding constants.^[^ ^20^, ^21^, ^22^, ^23^ ^]^ For instance, CE was exploited to determine the association and dissociation rates of the GroEL–GroES complex in the presence of ATP or ADP.^[^ ^24^ ^]^

The interfacing of CE with MS (CE–MS) represents a powerful and sensitive analytical tool for the characterization of intact proteins and can provide information on the protein dynamics and conformational states.^[^ ^16^, ^25^, ^26^, ^27^, ^28^, ^29^ ^]^ Taverna and co‐workers^[^ ^25^ ^]^ developed a CE–MS method capable of distinguishing the native form of antithrombin from its inactive latent form and demonstrated that both antithrombin conformers could associate during CE–MS analysis to form a non‐covalent heterodimeric complex. CE–MS is also very well suited to the analysis of oligomeric protein complexes, from dimers to multimeric assemblies.^[^ ^30^, ^31^, ^32^, ^33^, ^34^, ^35^ ^]^ Several groups reported CE–MS methods for relative quantitation of intact mAb dimeric forms.^[^ ^32^, ^33^, ^34^, ^35^ ^]^ Brodbelt and co‐workers^[^ ^36^ ^]^ reported a native CE–MS method for the characterization of ribosomal proteoforms. CE–MS methods were also developed to investigate protein–ligand interactions.^[^ ^37^, ^38^, ^39^ ^]^

Despite the increased sophistication of analytical instruments, native MS analysis of high molecular mass protein complexes is still challenging. In the nineties, Robinson and co‐workers^[^ ^40^, ^41^ ^]^ pioneered a preparative MS‐based platform to isolate and select macromolecular complexes, including GroEL, for ex situ downstream imaging by means of transmission electron microscopy (TEM) and atomic force microscopy. A few years later, the Heck group used MS and IMS–MS to study the binding properties of GroEL toward a variety of SPs.^[^ ^42^, ^43^ ^]^ The same group recently investigated new MS‐based analytical platforms using either infrared multiphoton dissociation or in‐gel cross‐linking mass spectrometry for native analysis of GroEL.^[^ ^44^, ^45^ ^]^ Belov et al. developed a two‐step MS‐based fragmentation approach that was applied to the characterization of GroEL monomeric subunits.^[^ ^46^ ^]^ Recently, for the first time, the Kelleher and co‐workers^[^ ^47^ ^]^ reported the CE–MS analysis of GroEL and used tandem MS to identify potential GroEL proteoforms. Nevertheless, until now, the potential of CE–MS for the detection and identification of ligand/protein‐bound GroEL complexes and the separation and characterization of structurally different GroEL conformers remained largely unexplored.

In the study presented here, we developed a CE–MS method, which allowed us to follow the functional cycle of GroEL‐assisted folding, detect the non‐covalent binding of various partners (nucleotides, co‐chaperones, and SPs) to GroEL, and analyze the induced conformational changes of GroEL under native conditions. The CE–MS method we developed is fast, reproducible, highly sensitive, and enables the injection of sample amounts at the amol level. In contrast to native MS approaches for characterizing GroEL, no buffer exchange of the protein samples with MS‐friendly buffers is required prior to CE–MS analysis. As a result, our method substantially minimizes sample consumption and sample loss, simplifies and shortens the analytical workflow, and allows one to maintain GroEL with minimized potential structural alterations at near‐physiological conditions. The method was applied to the analysis of several ligand/protein‐bound GroEL complexes. The detection of SP‐GroEL complexes did not induce a noticeable shift of the migration time compared to apo‐GroEL, since the SP is presumably not encapsulated inside the chaperonin nano‐cage in the absence of nucleotide and GroES. However, the nucleotide‐GroEL and GroES–GroEL complexes demonstrated a characteristic migration time shift corresponding to the substantial conformational rearrangements of the GroEL complexes. Native CE‐pseudo‐MS^3^ analyses of WT GroEL and two mutated GroEL variants were also performed. These experiments allowed us to confirm the sequence alterations at a high‐confidence level and highlighted subtle structural differences between WT GroEL and the two mutated counterparts. The results presented here demonstrate the superior performance of CE–MS for structural characterization of large intact protein complexes in comparison to direct infusion ESI–MS. The developed technique showed the potential for protein–ligand and protein–protein affinity studies of large ≈1 MDa non‐covalent multimeric protein complexes under near‐physiological conditions.

## 2 Results

### 2.1 CE–MS Method Development and Optimization

#### 2.1.1 Method Reproducibility and Detection Sensitivity of the Developed CE–MS Method

Bare fused silica (BFS) capillaries were used in this study for the CE–MS analysis of wild‐type (WT) and mutant GroEL complexes. For method development and optimization, native CE–MS experiments were performed with a mutated variant of WT GroEL_14_ (called thereafter mutant GroEL_14_), in which the three intrinsic solvent‐exposed cysteine residues (i.e., located not in the close proximity to the interface surface of the two‐rings’ interactions) were mutated into alanine residues (Figures S1A,B, Supporting Information). Adsorption‐related phenomena resulting in peak tailing were not observed for the investigated WT and mutant GroEL complexes using these uncoated capillaries. At pH 6.7, the GroEL monomeric subunit (theoretical pI 4.85) is negatively charged (see Figure S2 for a schematic representation of the GroEL migration under our CE–MS conditions, Supporting Information), and, conceivably, the tetradecameric GroEL_14_ complex is repulsed from the negatively charged inner silica surface due to its high density of negative charges of free silanol groups. As a result, the run‐to‐run intra‐day RSDs of migration times (t_m_), peak heights, and peak areas were less than 0.7%, 5%, and 6%, respectively, as determined with three consecutive CE–MS analyses of mutant GroEL_14_ (6 fmol equivalent injections) performed the same day on the same capillary. An inter‐day comparison (three CE–MS analyses of mutant GroEL_14_ performed on three different days (day 1, day 8, and day 31) on the same capillary) provided RSDs less than 0.8%, 8% and 9% for migration times, peak heights, and peak areas, respectively.

To achieve the best sensitivity in the CE–MS analysis of the 14‐mer GroEL, we examined the in‐source desolvation (ISD) energy range from 25 to 300 V. Highly efficient declustering of ammonium ions and desolvation was obtained for ISD levels ≥100 V. An ISD value set at 100 V resulted in the highest MS signal intensity levels, while preserving the integrity of the tetradecameric complex (Figure S3, Supporting Information). A decreased ISD value (≤100 V) resulted in a high decrease of the MS signal intensities (threefold and tenfold for 50 and 25 V, respectively, as compared to the signal intensities recorded at 100 V). Using an ISD of 150 V, the signal intensities were increased ≈1.5‐fold, compared to those recorded at 100 V, but traces of the 13‐mer GroEL were detected, indicative of subunit ejection. For ISD ≥ 200 V, the partial dissociation of the 14‐mer GroEL into the 13‐mer GroEL was even more significant. Yet, the quantities of GroEL_13_ detected in these CE–MS analyses were quite low (≤8%) even with the maximum ISD value of 300 V (Figure S3, Supporting Information), which was indicative of the high stability of the GroEL_14_ multimeric complex. In comparison, the same kind of experiments performed by direct infusion MS showed that an ISD value of 130 V was sufficient to generate ≈30% of GroEL_13_, and almost complete dissociation of the intact GroEL_14_ was observed for ISD set to 170 V (Figure S4A, Supporting Information). These results demonstrate that the gentle conditions of the developed CE–MS method enable efficient preservation of the native multimeric state of large protein complexes, which was evident from the charge state distribution. As shown in Figures S3 and S4 (Supporting Information), the increase of the ISD energy to 200–300 V resulted in a substantial increase in the charge state distribution of GroEL_13_ (from the maximum detected charge state of 37+ to 43+) and the corresponding shift toward detection of lower *m/z* values due to the partial unfolding of the complex by the applied desolvation energy. Additional experiments were carried out applying an in‐source collision‐induced dissociation (ISCID) energy (from 50 to 200 eV) in combination with an ISD energy (from 100 to 250 V). As expected, these experiments resulted in the detection of GroEL_13_ but, interestingly, an additional charge state distribution centered on the 53+ ion was detected, corresponding to a more folded state of GroEL_14_ (Figure S3O,P, Supporting Information).

The same range of ISD was applied to the CE–MS analysis of the co‐chaperonin GroES_7_. As shown in Figure S5 (Supporting Information), an ISD value of 50 V was sufficient to extract one monomer from the heptameric GroES_7_. For ISD set to 100 V, both GroES_6_ and GroES_5_ could be detected, but GroES_7_ was still the most abundant detected species (≥70%). At ISD values ≥150 V, almost complete dissociation of the native 7‐mer GroES was observed. Finally, the highest signal intensity levels for GroES_7_ were recorded with an ISD of 100 V. Based on these results, an ISD voltage of 100 V was selected as an optimal parameter to increase the sensitivity in the CE–MS analysis of GroEL, GroES, and GroEL–GroES complexes.

**Figure 2:**
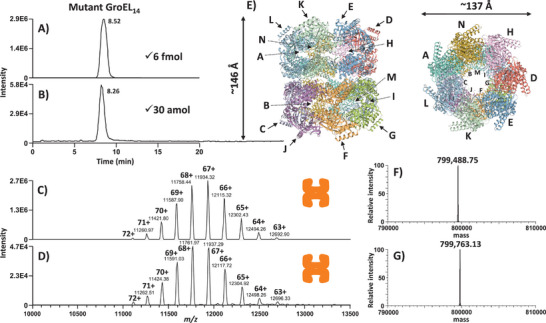
Native CE–MS analysis of 1 MDa multimeric protein assemblies. Base peak electropherograms (BPEs) were recorded in the CE–MS analysis of A) 6 fmol and B) 30 amol of 14‐mer mutant GroEL. The mass spectra presented in panels (C) and (D) were integrated across the BPE peaks shown in (A) and (B), respectively. F, G) Deconvolution of the CE–MS signal acquired during the migration time range of GroEL_14_ (i.e., between 8 and 9 min) using injected quantities equivalent to 6 fmol and 30 amol, respectively. The 3D higher‐order structure of the 14‐mer mutant GroEL is presented in panel E (side and top views are shown on the left‐ and right‐hand corners, respectively). The 14 subunit chains of the tetradecamer are labeled with letters A–N. (See Experimental Section for more details about the computational modeling of the GroEL_14_ 3D structure).

Next, we evaluated the limit of detection (LOD) and sensitivity of the CE–MS method (**Figure** **2**). Volumes as low as 1 nL, corresponding to ≈6 fmol of GroEL_14_ (injected at the concentration of 6 µm), could be typically injected in the CE–MS analysis of the tetradecameric GroEL_14_ complex. Figure 2A presents a characteristic base peak electropherogram (BPE) obtained from the CE–MS analysis of ≈6 fmol of mutant GroEL_14_. The deconvolution of the CE–MS signal (acquired at the MS resolution of 3125) resulted in an average experimental molecular mass (Mr_exp_) of 799 488.75 Da (Figure 2C,F), which was in a close match (ΔMr, 577 ppm) to the theoretical molecular mass (Mr_th_) of the mutant GroEL_14_ (799 027.77 Da, see Experimental Section), which did not account for coordinated cofactors and post‐translational modifications. A set of experiments was performed varying the MS resolution from 3125 to 50 000 at 200 *m/z* in the CE–MS analysis of GroEL_14_. As shown in Figure S6 (Supporting Information), a closer match to the Mr_th_ of GroEL_14_ was obtained at higher MS resolution values. With the resolution of 50 000, the difference between the Mr_th_ and Mr_exp_ of the mutant GroEL_14_ was 155 Da (i.e., 194 ppm), which can be attributed to incomplete desolvation or nonspecific binding of sodium, potassium, ammonium, and/or other adduct ions (e.g., the binding of six Na^+^ and one NH_4_^+^ adduct ions to GroEL_14_ would induce a mass error of 156 Da in the determination of the Mr_exp_ of the mutant GroEL_14_). However, as can be observed in Figure S6 (Supporting Information), the increase of the MS resolution from 3125 to 50 000 resulted in a >60‐fold decrease in the acquired MS signal intensity levels and an approximately eightfold decrease in the signal‐to‐noise (S/N) ratios. We, therefore, selected a resolution of 3125 to achieve the best LOD and sensitivity levels in the CE–MS analysis of GroEL_14_. At this MS resolution of 3125, the injection of quantities as low as 30 amol enabled the reliable detection of the intact GroEL_14_ protein at a S/N >25 (Figure 2D,G).

To assess the capability of the developed CE–MS method to differentiate the WT GroEL_14_ from the genetically mutated GroEL_14_ variant with minor perturbations in protein sequence (i.e., only four amino acids were replaced in the monomer sequence in comparison to the WT), CE–MS analysis of WT GroEL_14_ was also performed (Figure S7A,C, Supporting Information). Based on the deconvolution of the acquired CE–MS signal, the average Mr_exp_ (80 1238.19 Da at the MS resolution of 3125) closely matched (ΔMr, 588 ppm) to the Mr_th_ of WT GroEL_14_ (Mr_th_ 80 0767.13 Da, see Experimental Section). As expected, no significant migration time shift was observed between the WT and mutant GroEL_14_, which both migrated at 8.5 ± 0.2 min (*n* = 3). Additional experiments were performed with the co‐injection of WT and mutant GroEL_14_ and resulted in the detection of two different charge state envelopes corresponding to WT (*m/z* 11 611.55 at 69+) and mutant (*m/z* 11 585.65 at 69+) GroEL_14_, respectively (Figure S7D,E, Supporting Information). As observed for the mutant GroEL_14_, a more folded state of the WT GroEL_14_ was detected in specific CE–MS conditions applying a combination of ISD and ISCID voltages (Figure S3Q,R, Supporting Information).

For GroES_7_, the set of CE–MS experiments performed with the MS resolution ranging from 3125 to 50 000 at 200 *m/z* showed that the average Mr_exp_ of GroES_7_ (72 705.53 Da at the resolution of 50 000; Figure S8, Supporting Information) was the closest match (ΔMr, 43 ppm) to the Mr_th_ of the non‐truncated form of GroES_7_ (72 708.65 Da, see Experimental Section). These results confirmed the previously reported findings, which showed that the N‐terminal methionine of GroES was not truncated in vivo, contrary to GroEL, based on the results of N‐terminal sequencing.^[^ ^48^ ^]^ Besides, in GroEL and GroES, the N‐terminal methionine residue is bound to alanine (Figure S1A, Supporting Information) and asparagine (Figure S1C, Supporting Information) residues, respectively, which were commonly reported as cleavable and non‐cleavable residues, respectively.^[^ ^49^ ^]^

To benchmark our results against existing native MS approaches, direct infusion nanoESI‐MS analyses of SEC isolates of GroEL_14_ complexes were performed (Note S1, Supporting Information). These experiments resulted in a noticeably lower spectral quality (i.e., the signal was noisier, and broader peaks were observed in acquired spectra, perhaps due to adduct formation), a decreased sensitivity (≈13‐fold), and the detection of a less folded state, i.e., more denatured state of the GroEL_14_ complexes, compared to the data acquired using our developed CE–MS method (Figure S4B,E, Supporting Information). The partial unfolding of the GroEL_14_ complexes may be potentially due to SEC‐driven depletion of critical cofactor ions, e.g., Mg^2+^ and K^+^ ions that are present in a free‐state in the buffer solution, in which the GroEL_14_ sample was diluted, or that diffused out from the cation binding pockets of GroEL_14_ into the mobile phase during the SEC fractionation. As reported in numerous structural biology studies^[^ ^7^, ^50^, ^51^, ^52^, ^53^ ^]^ and demonstrated in our presented work (see below), Mg^2+^ and K^+^ ions have a fundamental role in the stabilization and folding of the GroEL_14_ complexes. The applied CE–MS conditions allowed us to maintain the structural integrity of the injected GroEL_14_ non‐covalent complexes with coordinated cofactors in a medium enriched with these necessary metal ions. The offline nanoESI‐MS analyses of the SEC isolates also required substantially larger amounts (≥6000‐fold) of GroEL_14_ complexes for sample preparation and direct infusion analyses, in comparison to our online CE–MS method. Overall, these results demonstrated the superior performance of our developed CE–MS method versus nanoESI‐MS analysis of SEC isolates in regards to sample consumption and detection sensitivity, which are two critical factors for the analysis of minute amounts of biological material, but also for preserving the native state of the GroEL_14_ complexes.

#### 2.1.2 Separation Performance of the Developed CE–MS Method

**Figure 3:**
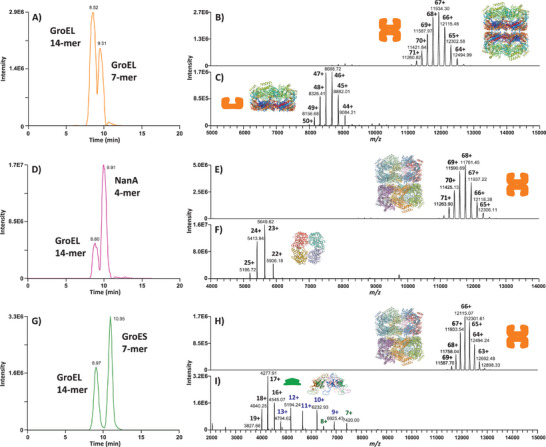
Separation performance of the developed CE–MS method. A–C) CE–MS analysis of GroEL_14_ and GroEL_7_ mutants under native conditions. A) BPE profile obtained from the co‐injection of 6 fmol of GroEL_14_ and 24 fmol of GroEL_7_. Two peaks are partially separated. The mass spectra integrated across the first and later migrating species are depicted in panels B) and C), respectively. Based on the detected charge state distributions, the first peak corresponds to the double‐ring GroEL_14_ and the second one to the single‐ring GroEL_7_. The 3D structures presented in panels B and C correspond to the computationally predicted higher‐order structures of the DR and SR mutant GroEL, where the N‐ and C‐termini of the monomeric subunits are colored in dark blue and red, respectively. D–F) CE–MS analysis of GroEL_14_ and NanA_4_ under native conditions. D) BPE profile obtained from the co‐injection of 6 fmol of GroEL_14_ and 2.5 fmol of NanA_4_. The integrated mass spectra of the first and later migrating species are depicted in panels E) and F), respectively. Based on the detected charge state distributions, the first peak corresponds to GroEL_14_ and the second one to the tetrameric NanA_4_ enzyme. G–I) CE–MS analysis of GroEL_14_ and GroES_7_ under native conditions. G) BPE recorded in the CE–MS analysis of equimolar quantities of GroEL_14_ and GroES_7_ (3 fmol of each chaperonin were co‐injected). H) Mass spectrum integrated across the first separated peak showing the characteristic charge state distribution of GroEL_14_. I) Mass spectrum of the later migrating species. The predominant charge state distribution centered around the 17+ ion corresponds to GroES_7_. Small amounts of GroES_6_ and GroES_5_ derived from ESI‐ and/or in‐source‐induced fragmentation are also observed. (See Experimental Section for the computational modeling of the protein 3D structures presented in panels B, C, E, F, H, and I).

To further demonstrate the separation performance of the CE–MS method, several oligomeric protein assemblies were analyzed in their native state, including the heptameric single‐ring (SR) variant of GroEL (see Experimental Section). The developed CE–MS method resulted in the separation (at resolution ≥0.7) of the tetradecameric (Mr_th_ 799 027.77 Da) and heptameric (Mr_th_ 399 479.45 Da) forms of GroEL (**Figure** **3**A–C). As shown in Figure 3A, the SR GroEL_7_ migrated ≈1 min later than the double‐ring (DR) GroEL_14_ mutant. It was noticed that the ion intensity of the SR GroEL_7_ was ≈5 times lower than that of the DR GroEL_14_, as determined by the detected MS signal intensity levels of the two types of GroEL homo‐oligomers injected at a 1:4 (DR:SR) molar ratio. The tetradecameric GroEL_14_ complex is formed by two identical heptameric rings that are stacked back‐to‐back (Figure 3B) and associated mainly by hydrophobic interactions. When the inter‐ring interactions are abolished by mutations, the dimerization of the two rings into the DR GroEL_14_ is prevented. Both uncoupled single‐rings are physiologically active binders (i.e., they can bind ATP nucleotide cofactor, GroES, and SPs) and possess a structure similar to those of the 14‐mer GroEL rings except for the GroEL hydrophobic interface between the two rings, which becomes fully solvent‐accessible in the SR GroEL_7_. In contrast, it is less exposed to the aqueous environment in the DR GroEL_14_. We hypothesize that the lower MS signal intensity of the SR GroEL_7_, when compared to the DR GroEL_14,_ is related to a lower ionization efficiency, which could be mainly due to its overall more hydrophobic (i.e., open cavity), less ionizable (i.e., a lower net charge per complex), and less polar surface. A twofold decreased number of readily ionizable, polar, hydrophilic residues exposed on the surface of the SR compared to the DR could contribute to the lower ionization efficiency of the 7‐mer SR.

GroEL_14_ interacts with nascent SPs after their ribosomal synthesis as well as misfolded SPs and promotes their appropriate folding (Figure 1). In the CE–MS analysis, we successfully detected the native form of N‐acetyl neuraminic acid aldolase (NanA, Mr_th_ 129 849.09 Da), a tetrameric enzyme that is known to interact with GroEL_14_ in vivo^[^ ^1^, ^5^, ^6^ ^]^ (Figure 3D–F). As depicted in Figure 3D, after mixing purified and natively folded GroEL_14_ with purified and natively folded NanA_4_ (no folding reaction cycle), the tetrameric NanA_4_ could be effectively separated from GroEL_14_ (at resolution ≥0.9). The CE–MS method was also applied to the analysis of dihydrodipicolinate synthase (DapA, Mr_th_ 124 555.09 Da), another obligate GroEL_14_ SP that possesses a similar structure and molecular mass as NanA.^[^ ^1^, ^5^, ^6^ ^]^ Surprisingly, the native tetrameric form of DapA could not be detected using our optimized CE–MS conditions, most probably due to its adsorption onto the inner surface of the BFS capillary or aggregation under the applied experimental CE–MS conditions since the theoretical pI of DapA monomer (≈5.98) is close to the pH of the BGE (6.7).

The co‐injection of GroEL 14‐mer and GroES 7‐mer resulted in the baseline separation of the two chaperonins at resolution ≥1.8 (Figure 3G–I). As expected, GroES_7_ migrated later than GroEL_14_ due to a lower density of negative charges at pH 6.7 (theoretical pI of GroES monomer ≈5.15). Finally, the migration pattern of the analyzed oligomeric assemblies showed a strong correlation with the net charge of the protein complexes, which was highly dependent on the pI of the protein monomer as well as the total number of monomeric subunits.

### 2.2 Native CE–MS Analysis of GroEL‐SP Complexes

GroEL‐assisted folding is an ATP‐dependent reaction cycle, which begins with the binding and trapping of non‐native SPs near the inner edge of the GroEL central cavity.^[^ ^4^, ^6^, ^54^, ^55^ ^]^ In the subsequent step, the GroEL‐SP complex binds the ATP nucleotide and GroES, which induces a substantial conformational rearrangement of the GroEL central cage, and results in the displacement and encapsulation of the SP into the chaperonin central cage (Figure 1). We applied the developed CE–MS method to the analysis of GroEL_14_ in complexation with SPs that interact with GroEL in *E. coli*. Two SPs, NanA and DapA, were used in these experiments carried out in the absence of ATP and GroES_7_, such that the SPs would bind to GroEL, but folding would not be possible, and the SPs would not be released. Prior to their incubation with GroEL_14_, NanA and DapA native tetramers were denatured with GuHCl. The binding of the denatured monomers of these SPs to GroEL_14_ was performed by diluting them into a GroEL_14_‐containing buffer (see Experimental Section). The residual low concentration of denaturing reagent (200 mm) in the incubation mixture did not affect the native folded state of GroEL_14_.

**Figure 4:**
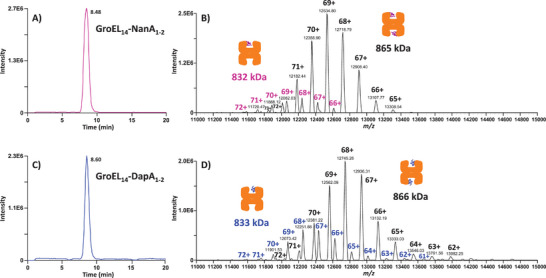
Native CE–MS analysis of SP‐bound GroEL_14_ complexes. BPEs recorded in the CE–MS analysis of A) GroEL_14_‐NanA and C) GroEL_14_‐DapA complexes. In each experiment, 6 fmol of GroEL_14_ were injected with an equimolar ratio (or a slight excess) of SP monomers (the SPs were denatured with GuHCl before their incubation with GroEL_14_, as described in the Experimental Section). B and D) Mass spectra integrated across the main peaks detected in the respective BPEs. The two charge state distributions observed in the mass spectrum of GroEL_14_ in complexation with NanA (B) indicate the binding of one and two NanA monomers to the 14‐mer GroEL chaperonin, respectively. The same binding stoichiometries were measured for DapA, as determined with the two different charge state distributions detected in the mass spectrum of the GroEL_14_‐DapA sample (D). Apo‐GroEL_14_ was not detected in these experiments.

As shown in the BPE profile in **Figure** **4**A, the CE–MS analysis of the GroEL_14_‐NanA sample resulted in the detection of a single peak migrating at ≈8.5 min. The mass spectrum integrated across this main electrophoretic peak revealed two charge state envelopes centered on the 68+ and 69+ ions, respectively (Figure 4B). The first envelope, the least abundant one (≈10% of the total signal intensity), corresponded to GroEL_14_ complexed with one NanA monomer, whereas the second envelope (≈90% of the total signal intensity) corresponded to GroEL_14_ bound to two NanA monomers (Table S1, Supporting Information). The GroEL_14_‐NanA complexes proved to be very stable *in solution* overtime, as demonstrated with the CE–MS analysis of the same GroEL_14_‐NanA sample stored at 4 °C for four days, which resulted in the detection of two charge state envelopes corresponding to the GroEL_14_‐NanA_1_ and GroEL_14_‐NanA_2_ complexes (Figure S9, Supporting Information). Compared to the freshly prepared GroEL_14_‐NanA sample, similar relative abundances of both complexes with the above‐shown stoichiometries were measured in the stored sample.

The BPE recorded in the CE–MS analysis of the GroEL_14_‐DapA sample led to the detection of one single peak migrating at ≈8.6 min (Figure 4C). Two charge state distributions with a prominent charge state at 68+ were observed in the mass spectrum integrated across this peak, which corresponded to GroEL_14_‐DapA_1_ and GroEL_14_‐DapA_2_ complexes (Figure 4D; Table S1, Supporting Information). The GroEL_14_‐DapA_2_ complex was detected in much higher abundance (≈80% of the total signal intensity) than the GroEL_14_‐DapA_1_ complex with a binding stoichiometry of 14:1.

The above‐described results showed that the developed CE–MS method could preserve non‐covalent and nonspecific hydrophobic interactions between the near 1 MDa GroEL_14_ chaperone and two natural in vivo substrates, NanA and DapA, and quickly measure the binding stoichiometries. In the absence of ATP nucleotide and GroES, the trapping of the SP inside the GroEL cavity (i.e., folding chamber) does not induce a substantial conformational alteration of the chaperonin subunits. Also, the mass difference between unbound GroEL_14_ and GroEL_14_ bound to NanA or DapA is minor (≤4% when one SP monomer is bound to GroEL_14_). This may explain why no significant migration time shift was observed for the SP‐bound GroEL_14_ complexes in comparison to apo‐GroEL_14_, since the electrophoretic mobility (µ_ep_) of a protein is governed by the ratio between its net charge and its hydrodynamic size. Overall, these results indicate that the CE–MS method can be applied to the analysis of the ≈300 natural GroEL SPs, since their binding to GroEL_14_ also involves nonspecific hydrophobic interactions.

### 2.3 Native CE–MS Analysis of GroEL‐Nucleotide Complexes

The binding of ATP to GroEL dramatically alters the conformation of the apical and intermediate domains of each GroEL subunit and results in a substantial enlargement of the GroEL central cage.^[^ ^56^, ^57^, ^58^ ^]^ To check if these conformational changes could be detected using our developed CE–MS method, GroEL_14_ was co‐injected with ATPγS (Mr_th_ 523.25 Da), a slowly hydrolyzed ATP analog, in which one of the oxygen atoms attached to the 3‐phosphate group is replaced by a sulfur. In the CE–MS conditions using a BGE of 50 mm ammonium acetate pH 6.7, the GroEL_14_‐ATPγS complex injected in the capillary was not detected even if the nucleotide amount incubated with GroEL_14_ prior to the CE–MS analysis was >300‐fold higher than that of the chaperonin. In each experiment where GroEL_14_ and ATPγS were co‐injected after a short incubation time (≈5 min), only ligand‐free GroEL_14_ was detected by CE–MS in the selected scan range of *m/z* 2000–300 000. The nucleotide binding is highly specific to the ATP‐binding domain and mainly involves hydrogen and ionic bonds. One hypothesis was that the GroEL_14_‐nucleotide complex was destabilized by the high electric field (≈22 kV m^−1^) applied across the capillary and dissociated during the CE–MS analysis. Indeed, previously reported studies showed that the electric field applied across the capillary could affect non‐covalent protein–ligand interactions, and result in the dissociation of the protein–ligand complex through an increase of the molecular dissociation constant of the complex.^[^ ^59^ ^]^ Another hypothesis was that the GroEL_14_‐nucleotide complex dissociated during the electrospray ionization process and/or was fragmented in the MS source following the application of ISD voltage. Yet, the experiments performed with GroES did not support this latter hypothesis (see the discussion on the CE–MS analysis of the GroEL–GroES complex below).

**Figure 5:**
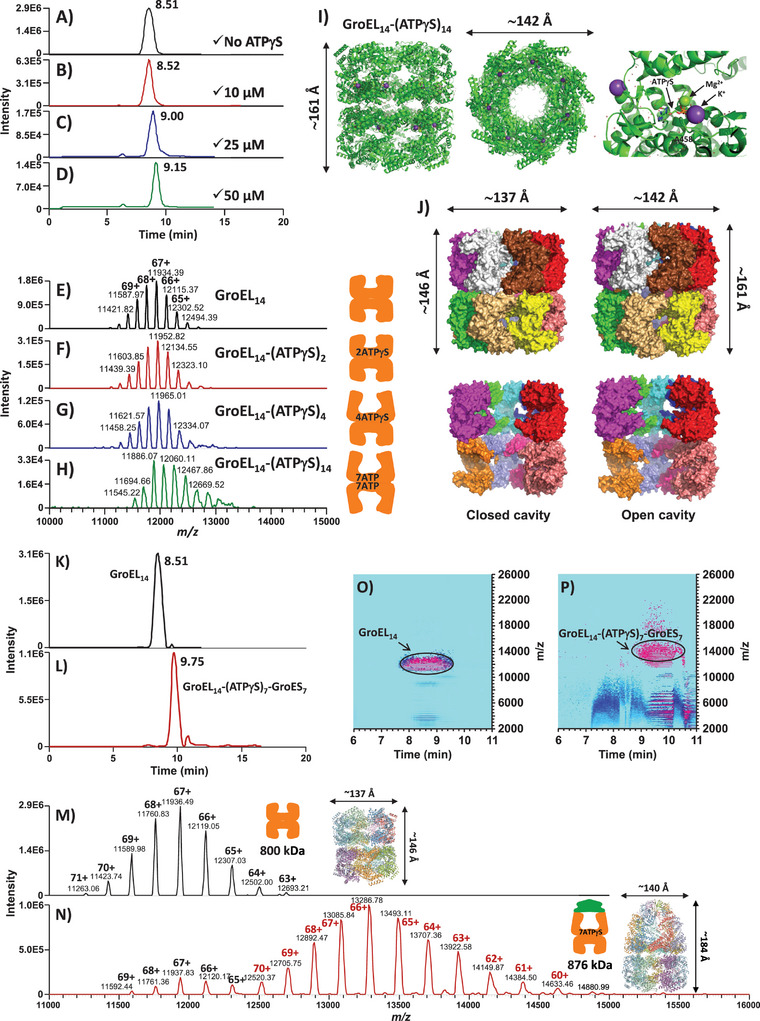
Native CE–MS analysis of GroEL_14_–(ATPγS)_n_ and GroEL_14_–(ATPγS)_7_–GroES_7_ complexes. Panels A–H) Co‐injection of GroEL_14_ and ATPγS (6 µm and 1 mm, respectively) in a capillary filled with 50 mm ammonium acetate BGE containing increased concentrations of ATPγS (from 0 to 50 µm). A–D) Recorded BPEs in the selected CE–MS conditions. E–H) Mass spectra integrated across the BPE peaks shown in panels A–D, respectively. Panel I) Computationally predicted 3D structure of the mutant GroEL_14_–(ATPγS)_14_ complex (side and top views are presented on the left and middle of the panel, respectively), where each subunit is bound to one ATPγS molecule. One nucleotide‐binding site is shown in the zoomed‐in region presented on the right. ATPγS is coordinated with Mg^2+^ and K^+^ cations, which are represented as green and purple balls, respectively. The mutated Ala 458, circled in dotted lines, can be in the vicinity of the nucleotide. Panel J) Surface representations of the 3D structures of unliganded mutant GroEL_14_ (left column) and mutant GroEL_14_–(ATPγS)_14_ (right column) complexes. The structural rearrangement of the central cages is clearly seen with the 3D structures where two and three front subunits were removed from the top and bottom rings, respectively, in the apo‐GroEL_14_ and GroEL_14_–(ATPγS)_14_ complexes (bottom row of the panel). Panels K, L) BPEs recorded following the injection of K) apo‐GroEL_14_ (6 µm) and L) GroEL_14_ and GroES_7_ (equimolar ratio of 6 µm) incubated with ATPγS (1 mm) and BeF_3_ (1 mm). The mass spectra integrated across the respective BPE peaks show the characteristic charge state distributions of apo‐GroEL_14_ (M) and GroEL_14_–(ATPγS)_7_–(BeF_3_)_7_–GroES_7_ (N) complexes. Panels O, P) Ion density maps of the CE–MS analyses of O) apo‐GroEL_14_ and P) GroEL_14_–(ATPγS)_7_–(BeF_3_)_7_–GroES_7_ complexes. The estimated sizes of the detected complexes are indicated in the panels M, N, based on the computationally predicted 3D structures of both complexes.

Several strategies were tested to stabilize the GroEL_14_–ATPγS complex during the CE–MS analysis. Assuming ATPγS dissociated from GroEL_14_ under the electric field, one strategy was to add a small amount of ATPγS in the BGE, such that the nucleotides present in the BGE could rapidly replace the nucleotides escaping from the ATP binding pockets of GroEL_14_ in the course of the CE analysis. Experiments were performed with the injection of a mixture of GroEL_14_ (6 µm) and ATPγS (1 mm) in a capillary filled with BGEs containing increased concentrations of ATPγS (from 10 to 50 µm). This resulted in the detection of several GroEL_14_–(ATPγS)_n_ complexes at different stoichiometries (from 14:2 to 14:14) (**Figure** **5**A–H; Table S1, Supporting Information). The number of ATPγS molecules bound to GroEL_14_ increased with an increased concentration of nucleotide in the BGE. With the relatively low concentration level of 10 µm of ATPγS in the BGE, a complex with two ATPγS molecules bound to GroEL_14_ was detected. 25 µm of nucleotide in the BGE resulted in the detection of GroEL_14_ complexed with four ATPγS molecules. Finally, a concentration of 50 µm of ATPγS was sufficient to detect a complex where each of the fourteen GroEL subunits was bound to one ATPγS molecule. Interestingly, a shift in the migration time was observed in each CE–MS analysis where the stoichiometry of the detected GroEL_14_–(ATPγS)_n_ complex was altered. The GroEL_14_–(ATPγS)_2_ complex did not migrate significantly later than apo‐GroEL_14_ but was the first migrating species among all detected GroEL_14_–(ATPγS)_n_ complexes. The GroEL_14_–(ATPγS)_14_ complex was the latest migrating species and migrated ≈0.6 ± 0.2 min later than apo‐GroEL_14_ (Figure 5A–D). The binding of ATPγS to the GroEL subunits brings additional negative charges inside the nucleotide‐binding pockets located in the equatorial domains of GroEL_14_ and does not affect the net global charge at the surface of the tetradecameric GroEL_14_ assembly.^[^ ^50^ ^]^ Moreover, these negative charges are partially neutralized by positively charged magnesium and potassium ions that interact with ATPγS,^[^ ^60^ ^]^ as shown in Figure 5I. Consequently, we attributed the characteristic migration time shifts to the conformational rearrangement of the GroEL subunits upon complexation with the nucleotide. Figure 5J shows a schematic representation of the conformational alteration of the 14‐mer GroEL mutant analyzed in this study, following the coordination of fourteen ATPγS molecules. The rearrangement of the intermediate and apical domains in the GroEL_14_–(ATPγS)_14_ complex induces an enlargement of the central cages of both rings and results in an increased hydrodynamic volume of the (ATPγS)_14_‐bound GroEL_14_ complex compared to unbound GroEL_14_, which supports the observed lower µ_ep_ of the GroEL_14_–(ATPγS)_14_ complex using our CE–MS conditions. As the binding of at least 3–4 nucleotide molecules in one ring is required for driving a significant degree of enlargement of the GroEL_14_ central cage,^[^ ^61^ ^]^ a similar migration behavior was expected between apo‐GroEL_14_ and GroEL_14_‐(ATPγS)_2_ complex. The migration time shift observed for the GroEL_14_–(ATPγS)_4_ complex let us assume that occupying only four nucleotide pockets of one ring by ATPγS was already sufficient to induce a substantial enlargement of this ring.^[^ ^58^ ^]^

Similar experiments were performed without the addition of the nucleotide in the GroEL_14_ sample prior to CE–MS analyses performed with BGEs containing increased concentrations of ATPγS. These experiments led to similar results and demonstrated that the presence of ATPγS nucleotide in the injected GroEL_14_ sample was not necessary to detect the GroEL_14_–(ATPγS)_n_ complexes at different stoichiometries. Finally, the above‐described experiments showed that the addition of the nucleotide in the BGE was a successful strategy to stabilize the GroEL_14_–ATPγS complexes during the CE migration. They also demonstrated the capability of the highly sensitive CE–MS method to detect and quickly separate several GroEL_14_ conformers under near‐physiological conditions (<30 min/run without any tedious and time‐consuming sample preparation). The characteristic migration time shifts observed for the GroEL–(ATPγS)_n_ complexes detected using the developed CE–MS technique effectively demonstrate how CE can unambiguously distinguish protein complexes that may have been derived from adduction during the ESI process owing to high concentrations of salts or substrates from protein complexes that were formed *in the liquid phase* prior or during the CE separation. This additional piece of information represents a major advantage in comparison to direct infusion ESI–MS that was used in previous studies for the analysis of nucleotide‐bound GroEL complexes.^[^ ^43^, ^62^ ^]^ To increase the MS signal intensity levels of the detected GroEL‐nucleotide complexes impacted by the yet low µm nucleotide concentrations added to the BGEs, alternative strategies using metal cofactors were tested in the presence of the co‐chaperone GroES_7_, as described below.

### 2.4 Native CE–MS Analysis of GroEL–GroES Complex with ATPγS

The functional protein folding cycle involves the cooperative binding of ATP and GroES to GroEL (Figure 1). Also, it was shown that initial ATP binding to the equatorial binding sites of one GroEL ring was required to drive the association of GroES with GroEL.^[^ ^61^, ^63^ ^]^ As the native CE–MS method we developed was capable of preserving the binding of several nucleotide molecules to the GroEL subunits, we applied the developed method to the analysis of the GroEL–GroES complex. Preliminary experiments were carried out with the injection of a mixture of GroEL_14_ (6 µm), GroES_7_ (6 µm), and ATPγS (1 mm) in a capillary filled with 50 mm ammonium acetate BGE devoid of ATPγS. As expected, this experimental setup did not allow us to detect the GroEL_14_–GroES_7_ complex. For these experiments carried out in the absence of ATPγS in the BGE, the BPE profile showed two separated peaks (at resolution ≈0.8, Figure S10A, Supporting Information) corresponding to apo‐GroEL_14_ and apo‐GroES_7_, respectively (Figure S10B,C, Supporting Information). Yet, the detected peaks were not separated at the high resolution of ≈1.8, as observed with the co‐injection of GroEL_14_ and GroES_7_ in the absence of ATPγS both in the injected sample and BGE (Figure 3G–I). These results indicated that the GroEL_14_–GroES_7_ complex dissociated during the CE migration prior to the ionization process.

A set of experiments were carried out with the injection of a mixture of GroEL_14_ (6 µm) and GroES_7_ (6 µm) in a capillary filled with BGEs containing the increased concentration levels of ATPγS (from 10 to 50 µm). These experiments resulted in the detection of GroEL_14_–(ATPγS)_n_ (where *n* = 3–14) and GroEL_14_–(ATPγS)_7_–GroES_7_ complexes (Figure S10D, Supporting Information). As we observed previously with the CE–MS analysis of the GroEL_14_–ATPγS complex in the absence of GroES (see above), the stoichiometry of GroEL_14_–(ATPγS)_n_ varied depending on the ATPγS concentration in the BGE, and 50 µm of nucleotide in the BGE resulted in the detection of the GroEL_14_–(ATPγS)_14_ complex with both rings fully occupied with APTγS. As shown in Figure S10D (Supporting Information), the relative proportion of GroEL_14_–(ATPγS)_7_–GroES_7_ complex compared to GroEL_14_–(ATPγS)_n_ complex increased with an increased concentration of ATPγS in the BGE. With the low concentration level of 10 µm of ATPγS in the BGE, 4 ± 0.5% of GroEL_14_–(ATPγS)_7_–GroES_7_ was detected compared to GroEL_14_–(ATPγS)_3_, based on the most abundant charge state signal intensity of the detected complexes. Using 15, 20, and 25 µm of ATPγS in the BGE, the relative proportions of GroEL_14_–(ATPγS)_7_–GroES_7_ complex were 21 ± 2%, 31 ± 1%, and 40 ± 2%, respectively, compared to the GroEL_14_–(ATPγS)_n_ (where *n* = 5–9) complex. With 40 µm of ATPγS in the BGE, an equimolar ratio of GroEL_14_–(ATPγS)_7_–GroES_7_ and GroEL_14_–(ATPγS)_13_ complexes was detected, and with 50 µm of ATPγS, the GroEL_14_–(ATPγS)_7_–GroES_7_ complex was predominant, compared to the detected GroEL_14_–(ATPγS)_14_ complex, and accounted for ≈60 ± 3%. As expected, the absolute MS signal intensities of the detected complexes decreased proportionally to the amount of nucleotide added to the BGE. Figure S11 (Supporting Information) depicts a characteristic CE–MS analysis of a mixture of GroEL_14_ and GroES_7_ performed using 50 µm of ATPγS in the BGE. The recorded BPE profile showed two major separated peaks (Figure S11A, Supporting Information). The first migrating species corresponded mainly to GroEL_14_–(ATPγS)_14_ complex, with a relatively small amount of GroEL_14_–(ATPγS)_7_–GroES_7_ complex (<15%) (Figure S11B, Supporting Information). A high amount of apo‐GroES_7_ and a very small amount of GroEL_14_–(ATPγS)_7_–GroES_7_ complex (<2%) were detected under the second peak (Figure S11D, Supporting Information). The region between the two BPE peaks corresponded to the migration zone of the GroEL_14_–(ATPγS)_7_–GroES_7_ complex (Figure S11C, Supporting Information). Apo‐GroEL_14_ was not detected in these experiments. The ion density map acquired in the CE–MS analysis clearly showed two different charged ion distributions separated over time, corresponding respectively to GroEL_14_–(ATPγS)_14_ and GroEL_14_–(ATPγS)_7_–GroES_7_ complexes (Figure S11E, Supporting Information). The GroEL_14_–(ATPγS)_7_–GroES_7_ complex migrated ≈0.7 min later than the GroEL_14_–(ATPγS)_14_ complex. The ion density map also showed that apo‐GroES_7_ migrated later (≈0.8 min) than the GroEL_14_–(ATPγS)_7_–GroES_7_ complex. Based on X‐ray crystallography, electron microscopy (EM), and cryo‐EM, a plethora of previously reported studies showed that the conformations of GroEL_14_ and GroEL_14_–GroES_7_ complexes were dramatically different, following a major conformational change of GroEL_14_, triggered by the nucleotide binding, upon complexation with the co‐chaperone GroES_7_.^[^ ^6^, ^56^, ^57^, ^58^, ^61^, ^64^ ^]^ We, therefore, attributed the shift of the migration time between GroEL_14_–(ATPγS)_14_ and GroEL_14_–(ATPγS)_7_–GroES_7_ complexes to the substantial conformational rearrangement of GroEL_14_ upon complexation with the co‐chaperone GroES_7_ and seven ATPγS molecules, under the described CE–MS conditions. The mass difference of 69 kDa between GroEL_14_–(ATPγS)_14_ and GroEL_14_–(ATPγS)_7_–GroES_7_ complexes could not explain the significant migration time shift between both complexes since, as described above with the SP‐bound GroEL_14_ complexes, a mass increment of up to 66 kDa did not affect the migration time of the near 1 MDa GroEL_14_ protein assembly in the absence of a drastic conformational alteration of the GroEL_14_ folding chamber. Also, the net global charge of GroEL_14_ is not expected to be significantly altered following the complexation with the co‐chaperone since GroES_7_ is also negatively charged at the pH of the analysis.

Biochemical studies showed that the coordination of Mg^2+^ ions to the nucleotides bound to the GroEL subunits is required for the conformational rearrangement of GroEL and stabilization of the GroEL–GroES complex.^[^ ^7^, ^50^, ^51^, ^52^ ^]^ For this reason, all previous experiments were carried out at a low concentration of Mg^2+^ ions (15–75 µm) in the BGE (see Experimental Section and Figure S10D, Supporting Information). To assess the impact of Mg^2+^ ions on the formation and stabilization of the GroEL–GroES complex at the molecular level, designated CE–MS experiments were carried out with a BGE containing 15 µm of ATPγS with or without Mg^2+^ ions. As shown in Figure S12 (Supporting Information), the experiments performed with a BGE devoid of Mg^2+^ ions did not enable the detection of the GroEL_14_–(ATPγS)_7_–GroES_7_ complex. In contrast, the addition of at least 15 µm of Mg^2+^ to the BGE resulted in the successful detection of the GroEL_14_–(ATPγS)_7_–GroES_7_ complex (Figure S12, Supporting Information). These results convincingly demonstrate the fundamental role of Mg^2+^ ions in the stabilization of the GroEL–GroES complex in the course of CE migration.

To increase the signal intensity levels of the GroEL–GroES complex detected by CE–MS, we tested alternative strategies using metal cofactors, including aluminum trifluoride (AlF_3_)^[^ ^65^ ^]^ and trifluoroberyllate ion (BeF_3_^−^) that were reported for different applications previously.^[^ ^66^, ^67^, ^68^, ^69^ ^]^ Experiments were performed with the incubation of GroEL_14_ (6 µm), GroES_7_ (6 µm), ATPγS (1 mm), and AlF_3_ (1 mm) or BeF_3_^−^ (1 mm) prior to their injection in a capillary filled with 50 mm ammonium acetate BGE devoid of nucleotide. In our hands, the use of aluminum fluoride did not enable the stabilization and detection of the GroEL_14_–(ATPγS)_7_–GroES_7_ complex in the CE–MS analysis under the described conditions, but was successful in improving the detectability of the GroEL_14_–(ADP)_7_–GroES_7_ complex, as described below. The use of BeF_3_^−^ cofactor resulted in a substantial stabilization of the GroEL–GroES complex during both the CE–MS migration and ESI process and allowed us to detect the GroEL_14_–(ATPγS)_7_–GroES_7_ complex at a substantially higher intensity level (Figure 5K–N). Compared to the CE–MS experiments performed with a nucleotide‐containing BGE, the peak intensity level of the detected GroEL_14_–(ATPγS)_7_–GroES_7_ complex was increased ≈100‐fold. Considering that each ATPγS molecule was coordinated with one Be^2+^ cation, three F^−^ anions, one Mg^2+^ cation, and one K^+^ cation (see Experimental Section), the GroEL_14_–(ATPγS)_7_–GroES_7_ complex was detected with a mass accuracy of ≈529 ppm, the Mr_exp_ and Mr_th_ of this complex being 876 768.69 Da and 876 305.05 Da, respectively. However, other bound ions, cofactors, and water molecules could expectedly contribute to the mass difference. As shown in Figure 5K,L,O,P, these experiments confirmed the migration time shift (≈1.2 min) between the detection of apo‐GroEL_14_ and GroEL_14_–(ATPγS)_7_–GroES_7_ complex, and that the developed native CE–MS method could separate different conformational states of the tetradecameric GroEL chaperone.

### 2.5 Native CE–MS Analysis of GroEL–GroES Complex with ADP

GroEL–GroES association is driven by the initial binding of either ATP or ADP to the nucleotide‐binding sites of the GroEL subunits.^[^ ^56^, ^70^ ^]^ We carried out CE–MS experiments with ADP nucleotide in order to detect the GroEL–GroES complex with an experimental setup involving ADP instead of ATPγS. A set of experiments was performed with the injection of a mixture of GroEL_14_ (6 µm), GroES_7_ (6 µm), and ADP (1 mm) in a capillary filled with 50 mm ammonium acetate BGEs containing increased concentrations of ADP (from 10 to 50 µm). With the low concentration level of 10 µm of ADP in the BGE, the BPE profile showed one main peak migrating ≈8.9 min (a small peak corresponding to GroES migrated later at ≈10.2 min) (Figure S13A, Supporting Information). The mass spectrum integrated across the BPE peak showed two charge state distributions corresponding to GroEL_14_–(ADP)_3_ (Mr_exp_ 801 047.32 Da; ΔMr, 406 ppm), and GroEL_14_–(ADP)_7_–GroES_7_ (Mr_exp_ 877 768.03 Da; ΔMr, 2,374 ppm) complexes, respectively (Figure S13C and Table S1, Supporting Information). The low intensity level of the CE–MS signal of the GroEL_14_–(ADP)_7_–GroES_7_ complex and nonspecific adduction most probably explain the lower mass accuracy for the latter complex. The relative amount of GroEL_14_–(ADP)_7_–GroES_7_ complex accounted for ≈15% compared to the GroEL_14_–(ADP)_3_ complex (≈85%). Interestingly, when using 50 µm of ADP in the BGE, only the GroEL_14_–(ADP)_7_–GroES_7_ complex was detected (Figure S13B,D, Supporting Information), and a reproducible shift of the migration time (≈0.7 min) was observed compared to the analysis where GroEL_14_–(ADP)_3_ was the highest abundance detected species. These results showed that the GroEL_14_–(ADP)_7_–GroES_7_ complex had a lower µ_ep_ than the GroEL_14_–(ADP)_3_ complex in our CE–MS conditions, in accordance with a conformational change of GroEL_14_ upon complexation with GroES_7_ and seven ADPs.

Previously reported studies showed that AlF_3_ stabilized the association of GroEL with GroES in the presence of ADP.^[^ ^50^, ^52^, ^65^ ^]^ A set of experiments was carried out with the injection of a mixture of GroEL_14_ (6 µm), GroES_7_ (6 µm), ADP (1 mm), and AlF_3_ (1 mm) in a capillary filled with a BGE containing increased concentrations of ADP (from 10 to 50 µm). With 10 µm of ADP in the BGE, a similar BPE profile was recorded compared to the analysis devoid of AlF_3_ and the GroEL_14_–(ADP)_3_–(AlF_3_)_3_ (Mr_exp_ 801 289.26 Da; ΔMr, 394 ppm) and GroEL_14_–(ADP)_7_–(AlF_3_)_7_–GroES_7_ (Mr_exp_ 877 954.45 Da; ΔMr, 1,914 ppm) complexes were detected (Figure S14, Supporting Information). As shown in Figure S14B (Supporting Information), the thorough comparison of the ion charge state distributions of the GroEL_14_–(ADP)_3_ and GroEL_14_–(ADP)_3_–(AlF_3_)_3_ complexes clearly showed the *m/z* shift induced by the coordination of three AlF_3_ ligands, which resulted in a mass increment of 241.94 Da for GroEL_14_–(ADP)_3_–(AlF_3_)_3_, in good agreement with the expected theoretical mass shift of 251.93 Da. Interestingly, the relative quantities of GroEL_14_–(ADP)_7_–(AlF_3_)_7_–GroES_7_ and GroEL_14_–(ADP)_3_–(AlF_3_)_3_ complexes accounted for ≈35% and ≈65% in these experiments performed with the AlF_3_ metal cofactor, indicating a lower molecular dissociation rate for the GroEL_14_–(ADP)_7_–(AlF_3_)_7_–GroES_7_ complex in comparison to the dissociation kinetics of the GroEL_14_–(ADP)_7_–GroES_7_ complex. As expected, based on our above‐described experiments, using 50 µm of ADP in the BGE, only the GroEL_14_–(ADP)_7_–(AlF_3_)_7_–GroES_7_ complex was detected, with a lower signal intensity level.

Overall, these results demonstrated that the use of AlF_3_, concomitantly with the addition of a low ADP concentration in the BGE, was a successful strategy to stabilize the GroEL–GroES–ADP complex, and that the developed CE–MS method was capable of detecting the coordination of metal ions to the ≈1 MDa ligand‐bound GroEL complexes.

### 2.6 Structural Characterization of GroEL Complexes

#### 2.6.1 Structural Characterization of DR WT GroEL

**Figure 6:**
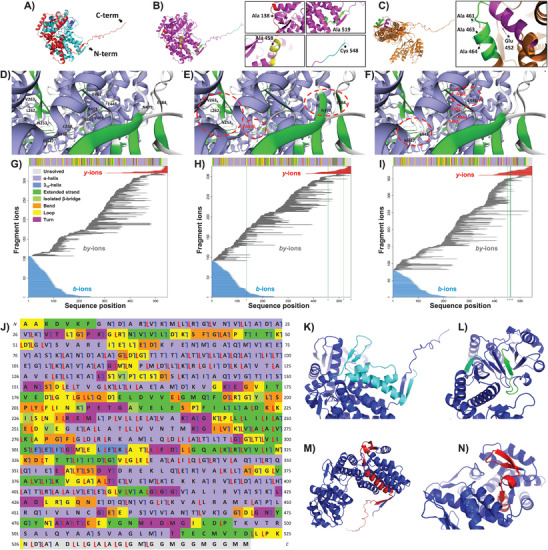
3D structures and native CE‐pseudo‐MS^3^ analyses of WT GroEL and in vitro cloned GroEL mutants. 3D structures of the monomeric subunits of double‐ring (DR) WT GroEL (A), DR mutant GroEL (B), and single‐ring (SR) mutant GroEL (C), based on the AlphaFold predicted structure of the WT GroEL monomer (see Experimental Section). In panel A, the sequence regions that were fragmented in the N‐ and C‐termini of the WT, resulting in the detection and assignment of *b*‐ and *y*‐type terminal fragment ions, as described in Panel G, are colored in cyan and red, respectively. The inserts in panels B and C depict the position of the mutated amino acids in the structural motifs of the DR and SR mutant GroEL, respectively (regions colored in red, yellow, green, and cyan in insert B, and in green and magenta in panel C correspond to sequence regions where the mutations were introduced). For the mutant DR, one mutation (M548C) was introduced at the end of the C‐terminus that corresponds to a loose loop based on the AlphaFold predicted structure of WT GroEL. For the mutant SR, the four amino acids that were mutated (R452E, E461A, S463A, V464A) are in the same vicinity in the C‐terminus of the sequence. D–F) Selected regions of the computationally predicted 3D structures of DR WT (D), DR mutant (E), and SR mutant (F) GroEL to illustrate locus‐dependent structural differences between the WT and its two mutated counterparts. Letters J, K, and L correspond to the subunit chain name of the oligomeric assemblies. The red dotted lines circle protein regions with altered structural motifs for the DR and SR GroEL compared to the WT, as predicted with the computational modeling of the WT and mutant 3D structures. The amino acids highlighted in red correspond to mutated amino acids. See the Experimental Section for the design of the computationally predicted higher‐order 3D structures of the monomeric subunits and oligomeric assemblies of the three analyzed GroEL species. G–I) Plots of the fragment ions detected and identified in native CE‐pseudo‐MS^3^ analyses of DR WT (G), DR mutant (H), and SR mutant (I) GroEL, applying HCD collisional energy. *b*‐ and *y*‐type terminal fragment ions are plotted as cyan and red lines, respectively, and *by*‐type internal fragment ions are plotted as gray lines. The *y*‐axis indicates the number of each type of fragment ions that were assigned for each GroEL species. The vertical green lines delineate the position of the mutated amino acids in the sequence of the DR (H) and SR (I) mutant GroEL. The color‐coded bars at the top of the fragmentation maps are motif‐scale bars that fit the amino acids to their corresponding structural motif in the WT and the mutated GroEL variants (light grey, unresolved region; light blue, *α*‐helix; skyblue, 3_10_‐helix; green, extended strand participating in *β*‐ladder; lime, one residue in isolated *β*‐bridge; orange, bend; yellow, loop; purple, hydrogen‐bonded turn; see Experimental Section and Figure S17, Supporting Information). J) Color‐coded fragmentation map showing the regions of the WT GroEL sequence covered by terminal and internal fragmentations (blue bars correspond to *b*‐type cleavages yielding *b*‐ or *by*‐type ions, and red bars correspond to *y*‐type cleavages yielding *y*‐ or *by*‐type ions) and their corresponding structural motifs (the color codes are identical to those described in panels (G–I). K–N) Computationally predicted AlphaFold structures of the WT GroEL monomers. The region highlighted in cyan in panel K corresponds to the N‐terminal sequence region from AA 2 to 73. The regions highlighted in greencyan and green in panel L correspond to the 186–191 and 208–217 AA sequence regions, respectively. The region highlighted in red in panel M corresponds to the C‐terminal sequence region from AA 477 to 548 (panel N is a top view of panel M showing the three *β*‐strands located in the 477–496 AA sequence region).

Native CE‐pseudo‐MS^3^ analysis of GroEL 14‐mer generated highly informative HCD MS^3^ spectra (Figure S15A–C, Supporting Information), which enabled unambiguous and accurate structural characterization of the chaperonin at a high‐confidence level (**Figure** **6**). CE‐pseudo‐MS^3^ analysis of WT GroEL_14_ (Figure S15D, Supporting Information) yielded 105 *b*‐type and 18 *y*‐type terminal fragment ions derived from the fragmentation of the N‐ and C‐termini of the WT monomeric subunits, respectively (Figure 6A,G), and 213 *by*‐type fragment ions derived from internal fragmentation of the subunits (Figure 6G). Conversely to terminal fragment ions that include either the N‐terminus (like *b*‐type ions) or the C‐terminus (like *y*‐type ions) of the protein backbone, internal fragment ions (like *by*‐type ions) are derived from multiple fragmentation cleavages of the protein backbone and do not include either of the protein sequence N‐ and C‐termini.^[^ ^71^, ^72^, ^73^, ^74^ ^]^ To minimize the false discovery rates, stringent parameters were selected in the search of internal fragments (e.g., narrow CE migration time isolation window of ≤0.5 min, internal fragment mass error tolerance of 1 ppm, and minimum internal fragment length of 5 amino acid residues (AA)). As additional validation of the reliability of internal fragment assignments, supplementary levels of manual data examination were applied (see Experimental Section). This manual evaluation allowed us to show that >60% of the identified internal fragments could be matched with terminal fragments. Also, the high level of purity of the analyzed WT GroEL, the high separation efficiency of the CE–MS method (i.e., fragments derived from multiple nonseparated precursor ions may lead to ambiguous identifications), and the requirement for the identified *b‐*, *y‐*, and *by*‐ions to be detected in the fragmentation spectra acquired during the defined CE migration time window of the corresponding complex of interest dramatically reduced the probability of false positive matches, and increased the confidence level of the provided results. We also noticed a strong relationship between the secondary structure motifs and their susceptibility to internal fragmentation. For instance, such unstructured protein sequence motifs as loops, bends, and turns were highly prone to internal fragmentation, based on the detection of higher numbers of internal cleavages corresponding to these motifs of the protein. Based on terminal *b‐* and *y*‐type fragmentation only, the sequence coverage of WT GroEL accounted for 23% with the detection of 123 inter‐amino acid residue cleavage sites (Figure S16A, Supporting Information). The inclusion of all identified internal fragments could potentially increase the sequence coverage of WT GroEL up to 63%, with the detection of 346 inter‐amino acid residue cleavage sites in total (Figure 6G,J; Figure S16B, Supporting Information). As shown in Figure 6J and Figure S16B (Supporting Information), the C‐terminus of the WT monomers from AA 476 to 547 was the least prone to HCD fragmentation. In this C‐terminal region, only 30% of the inter‐residue bonds were fragmented, whereas 68% of the inter‐residue bonds were fragmented in the N‐terminal region from AA 1 to 72, and 42% were fragmented in the less efficiently covered sequence region from AA 151 to 222. Based on the predicted secondary structure of WT GroEL (Figure 6J; Figure S17, Supporting Information), the C‐terminal region from AA 476 to 547 contains four *β*‐strands, one *α*‐helix, four loops, three turns, one bend, and an unresolved region (526–547 AA) of the protein, which may correspond to a loose loop based on the computationally predicted AlphaFold^[^ ^75^ ^]^ structure of WT GroEL (Figure 6M,N). No fragmentation was detected in the WT sequence regions from AA 185 to 190 and from AA 207 to 216, which are made up of one *β*‐strand and one bend (185–190 AA), and one turn, one residue in an isolated *β*‐bridge, one *β*‐strand, and one bend (207–216 AA), respectively (Figure 6J,L). Finally, the higher proneness to fragmentation of the N‐terminus of the WT subunits may be explained by a higher density of *α*‐helices and loops in the N‐terminal region. Indeed, twice as many amino acid residues are involved in *α*‐helices and loops in the 1–72 AA region, composed of three *α*‐helices and seven loops, compared to the C‐terminal 476–547 AA region (Figure 6J,K).

#### 2.6.2 Structural Characterization of DR Mutant GroEL

CE‐pseudo‐MS^3^ analysis of DR mutant GroEL 14‐mer (Figure S15E, Supporting Information) yielded 89 *b*‐type and 19 *y*‐type terminal fragment ions, and 216 *by*‐type internal fragment ions (Figure 6H). The sequence coverage of the DR mutant GroEL accounted for 20% with 108 cleaved sites based on terminal fragmentation only (Figure S18A, Supporting Information), and could be potentially increased up to 57% with the detection of 313 cleaved sites in total, when internal fragments were included (Figure S18B, Supporting Information). As observed for the WT, the C‐terminal region of the DR mutant was the least prone to HCD fragmentation. In the sequence region of the DR mutant from AA 476 to 547, where two mutations were introduced (Figure 6B,H; Figure S1B, Supporting Information), only 28% of the inter‐residue bonds were fragmented, whereas 65% and 44% of the inter‐residue bonds were fragmented in the N‐terminus from AA 1 to 72, and in the less efficiently covered sequence region from AA 176 to 247, respectively (Figure S18B, Supporting Information). Like in the WT GroEL, the sequence region of the DR mutant from AA 207 to 216 forms a turn, a residue in an isolated *β*‐bridge, a *β*‐strand, and a bend, based on the predicted secondary structure of the DR mutant (Figure S17, Supporting Information). This region is not prone to HCD fragmentation, based on the detection of only two cleavage sites in this portion of the sequence. Yet, noticeable differences were observed in the HCD fragmentation patterns of the WT and DR mutant GroEL. For instance, in the 475–486 AA sequence region, ≈50% of the inter‐residue bonds were fragmented in the WT GroEL, whereas half as many bonds were fragmented in this region in the DR mutant. Based on the predicted secondary structures of the WT and mutant GroEL, the two types of GroEL exhibit different structural motifs in this sequence region. The amino acid residues N479 and E484 are part of a loop in the WT but part of an extended strand in the DR mutant. Also, the amino acid residue E483 is part of a bend in the WT but part of a hydrogen‐bonded turn in the DR mutant (Figure 6D,E; Figure S17, Supporting Information). In contrast, the 259–263 AA sequence region was fragmented twice as much in the DR mutant compared to the WT. These specific fragmentation patterns may be derived from a subtle structural difference between the GroEL complexes since the amino acid residues L262 and V263 are part of an *α*‐helix in the WT but part of a loop and a turn in the DR mutant (Figure 6D,E). Moreover, the 301–307 AA sequence region, where E303, E304, and I305 are part of a turn in the mutant DR but a 3_10_‐helix in the WT (Figure S17, Supporting Information), also exhibits a specific fragmentation pattern with 80% and 100% of the amide bonds fragmented in the DR mutant and in the WT, respectively.

#### 2.6.3 Structural Characterization of SR Mutant GroEL

Native CE‐pseudo‐MS^3^ analysis of the SR mutant GroEL 7‐mer (Figure S15F, Supporting Information) resulted in the detection of 79 *b*‐type and 27 *y*‐type terminal fragment ions (Figure S19A, Supporting Information). Compared to the WT and DR mutant GroEL, a higher relative proportion of terminal *y*‐type ions (26% of the total number of terminal fragment ions versus 15% and 18% for the WT and mutant DR, respectively) was detected for the SR mutant. In addition, 5 *b*‐type and 18 *y*‐type terminal fragment ions were specific to the HCD fragmentation of the SR mutant and were not detected in the HCD fragmentation of WT GroEL (87% of these specific fragments were not detected as well in the HCD fragmentation of the DR mutant) (Figure S19A, Supporting Information). The four mutations introduced in the C‐terminus of the SR mutant sequence (Figure 6C; Figure S1B, Supporting Information) may explain a higher propensity to C‐terminal fragmentation. Internal fragmentation of the SR subunits yielded 279 *by*‐type fragment ions and could potentially increase the sequence coverage of the SR mutant from 19% to 70% and the number of inter‐amino acid cleavage sites from 106 to 384, compared to the results obtained with the inclusion of *b‐* and *y‐*type terminal fragments only (Figure 6I; Figure S19B, Supporting Information). As observed for the WT and the DR mutant, the C‐terminal region of the SR mutant was the least prone to HCD fragmentation, with only 39% of the inter‐residue bonds fragmented in the 476–547 AA sequence region. In contrast, 75% of the N‐terminus inter‐residue bonds from AA 1 to 72 and 61% of the inter‐residue bonds of the less efficiently covered sequence region from AA 151 to 222 were fragmented. Yet, noticeable differences were observed in the HCD fragmentation of the WT, DR mutant, and SR mutant GroEL. For instance, in the 446–458 AA sequence region, where R452 was mutated to E452 in the mutant SR (Figure 6C,F), only 33% and 42% of the inter‐residue bonds were fragmented in the WT and the DR mutant, respectively, versus 92% in the SR mutant. Based on the computationally predicted 3D structures of the WT and mutant GroEL complexes (Figure 6D–F; Figure S17, Supporting Information), M447 and E448 are part of an *α*‐helix in the WT and mutant DR but part of a turn in the mutant SR in this sequence region. Interestingly, as observed for the DR mutant, the 301–307 AA sequence region is less prone to fragmentation in the SR mutant compared to the WT (80% and 100% of the peptide bonds fragmented, respectively). In this sequence region, the DR and SR GroEL mutants exhibit the same structural motifs, whereas a structural difference is observed in the WT, where E303, E304, and I305 are part of a 3_10_‐helix instead of a turn (Figure S17, Supporting Information). The above‐described results support the hypothesis that the HCD fragmentation patterns acquired in the native CE‐pseudo‐MS^3^ analyses of the WT and mutant GroEL multimeric assemblies are highly dependent on the overall integrity of the quaternary structure, as well as on the elements of the tertiary and secondary structures.

## 3 Discussion

Most functionally active proteins form covalent and non‐covalent oligomeric complexes in cells of living biological systems ranging from bacteria to eukaryotes. Such protein oligomerization of protein–protein, protein–ligand, and macromolecular complexes is essential for enabling the appropriate folding state, stability, and biological function (e.g., enzymatic activity) of proteins involved in such complexes. The GroEL–GroES chaperonin system is a well‐studied and characterized protein folding machinery for which nucleotide binding‐induced conformations were characterized by high‐resolution structural biology techniques (e.g., X‐ray crystallography, cryo‐EM, NMR, and hydrogen–deuterium exchange). GroEL and GroES chaperones were used as model oligomeric protein assemblies to develop our CE–MS method and verify its ability not only to preserve non‐covalent interactions between GroEL and its binding partners, but also to detect the conformational rearrangements of the tetradecameric GroEL following its binding with ATPγS/ADP nucleotides and GroES that occur during the functional protein folding cycle. Based on the current knowledge of the GroEL conformations in specific experimental conditions, we assessed the GroEL conformations in our set of CE–MS experiments and determined that different GroEL conformers exhibited distinct migration times and could be rapidly separated and identified. Future experiments will aim at reproducing the complete functional protein folding cycle with the CE–MS analysis of the GroEL–GroES–SP complexes under near‐physiological conditions. We expect that the binding and encapsulation of the SP in the GroEL nano‐cage will induce a detectable by CE–MS conformational rearrangement of the tetradecameric chaperonin under our CE–MS conditions, and will result in a characteristic migration time shift, enabling the separation of the GroEL–GroES–SP complex from apo‐GroEL and GroEL–GroES complex. Further developments of the CE–MS technique could provide direct evidence of the conformational changes of the detected GroEL complexes, by hyphenating the CE–MS method with structural biology techniques. Recently, the Rauschenbach and Coon laboratories developed MS‐based instrumentation to deposit selected (i.e., separated or isolated) ions of protein complexes, using MS ion beams, onto EM grids and obtain a 3D reconstruction of GroEL and other complexes.^[^ ^76^, ^77^ ^]^ Similarly, the native CE–MS method we developed, which has the capability of separating protein complexes of interest in various isolates and matrices under native conditions, could be coupled to the deposition of specific protein complex species on cryo‐EM or TEM/SEM grids under the conditions specifically required for downstream imaging. Such a setup may allow one to acquire complementary data using native MS and cryo‐EM for the structural characterization of intact high‐purity multisubunit and macromolecular complexes under native conditions. This approach could potentially lead to the development of a more simplistic, versatile, flexible, and affordable approach for cryo‐EM sample deposition than the aforementioned MS–EM technologies.

Although the developed CE–MS method in its current state could not solve key structural features of protein complexes that can only be elucidated using dedicated biophysical/biochemical techniques, the presented here CE–MS approach appears attractive as a quick, sensitive, effective, and robust complementary method that can provide a wealth of information on protein–ligand and protein–protein interactions *in solution*. The developed CE–MS technique offers several major advantages in comparison to the techniques typically used in structural biology: 1) a time‐effective and straightforward workflow with minimal sample preparation, which results in the detection and characterization of the GroEL complexes in less than 30 min (as opposed to weeks/months of sample preparation and analysis using, e.g., X‐ray crystallography and cryo‐EM); 2) a very high sensitivity with a LOD of 30 amol, which is the equivalent of the GroEL content of ≈200–300 *E. coli* cells (the required amounts of biologically active proteins are ≈10 000‐fold lower compared to conventional structural biology techniques); 3) a high separation performance, which enables the separation of GroEL_14_, GroEL_7_, GroES_7_, and tetrameric enzymes as well as the separation of GroEL_14_–ATPγS/ADP and GroEL_14_–ATPγS/ADP–GroES complexes at different stoichiometries; 4) the capability to differentiate protein complexes that may have been derived from adduct formation during the electrospray ionization process from protein complexes formed *in the liquid phase* prior or during CE–MS analysis (such differentiation would not be possible using direct infusion ESI–MS); 5) the capability to detect quickly the conformational rearrangements of GroEL upon complexation with ATPγS/ADP nucleotides and GroES under near‐physiological conditions, based on characteristic migration time shifts; 6) while the developed method was effectively applied to the well‐studied GroEL–GroES chaperonin to benchmark our findings to the published data, the manuscript presents a methodological framework to study other protein and macromolecular complexes (large and small); and 7) in contrast to other biochemical and native MS techniques mentioned above, the developed CE–MS technique was able to detect low level (≈5%) microheterogeneities in the purified mutant double‐ring GroEL, presumably corresponding to the truncation of the C‐terminal cysteine residues. Such microheterogeneities were not reported in the past for WT or mutant GroEL. The alternative techniques are not capable of detecting such low stoichiometry microheterogeneities in large protein complexes. Recent studies reported the combination of mobility CE (enabling molecular hydrodynamic radius measurements) and native MS (enabling solvent accessible surface area (SASA) determination) to predict the 3D ellipsoidal geometry of globular proteins *in the liquid phase* (i.e., under near‐native physiological conditions).^[^ ^78^, ^79^ ^]^ Similarly, our native CE–MS method could be used for the computational modeling of the 3D ellipsoidal shape of GroEL or other proteins and protein complexes. Although of low‐resolution, this structural information could be acquired at high speed, high sensitivity, and low sample consumption, and could provide complementary and crucial information on the conformation of proteins and multimeric protein assemblies that is not directly accessible using gas‐phase techniques such as ion mobility spectrometry.

The GroEL chaperone was selected in our study as a model multimeric protein assembly to develop highly efficient native CE‐pseudo‐MS^3^ methods that could be applied to the structural characterization of other types of protein complexes and macromolecular species. Native CE‐pseudo‐MS^3^ analyses of WT GroEL_14_ and two genetically mutated GroEL_14_ variants, with minor alterations in the protein sequence compared to the WT GroEL monomer, generated high quality and highly informative HCD MS^3^ spectra. With the accurate assignment of terminal fragments and the reliable identification of hundreds of internal fragments, the sequence alterations were confirmed at a high‐confidence level, with a sequence coverage that could reach 55–60% (after stringent filtering of the predicted internal fragments that do not match their complementary terminal fragments in their breakage/fragmentation site locations), based on the selection of stringent parameters in the search for internal fragments and the application of several levels of filtering and manual validation. To the best of our knowledge, such a sequence coverage has not been previously reported for native top–down analysis of subunits ejected from macromolecular complexes of ≈1 MDa. The accurate and unambiguous assignment of internal fragments for large intact proteins is a relatively new field in top–down MS‐based proteomics that provides potential incentives for the development of sound statistical models and bioinformatics tools for the reliable assignment of internal fragments that would account for numerous challenges (e.g., frameshift ambiguity, *a*‐fragments derived from the decomposition of *b*‐fragments, and all the above discussed puzzles) to address the lack of readily available computational methods.^[^ ^71^, ^72^, ^73^, ^74^ ^]^ Our study reflects how CE–MS, in combination with cutting‐edge data processing software tools, can enable in‐depth and highly informative analysis of multimeric protein assemblies under near‐physiological conditions. Based on the computationally predicted protein 3D structures, we demonstrated that the specific HCD fragmentation patterns recorded in the CE‐pseudo‐MS^3^ analyses of WT and mutant GroEL_14_ were highly dependent on moderate locus‐dependent changes in their preserved elements of secondary and tertiary structures.

Cellular proteomes exhibit a colossal diversity of proteins and protein complexes with specific and sometimes unique physicochemical properties. We strongly believe that the developed native CE–MS technique can be applied to other macromolecular systems and protein assemblies, e.g., the less studied since technically more challenging mammalian TRiC (TCP‐1‐containing ring complex) chaperonin, a hetero‐hexadecamer interacting with ≈10% of newly synthesized proteins. Depending on the molecular mass, hydrodynamic volume, hydrophobicity, isoelectric point of the analyzed protein complexes, as well as their proneness to dissociate or aggregate *in solution* and their electromigration behavior, method optimization may be required. Bare fused silica capillaries were used in our study since these capillaries were found to be most appropriate to the CE–MS analysis of GroEL complexes after comparing them with other capillary surface chemistries, including the neutral polyacrylamide coating. However, neutrally‐ or positively‐coated capillaries may be selected for other types of protein complexes, as it was previously reported by our and other groups.^[^ ^33^, ^34^, ^36^ ^]^ We observed that the electric field applied across the capillary did not destabilize the GroEL–NanA and GroEL–DapA complexes, but could possibly destabilize the GroEL‐nucleotide complexes to some extent in specific experimental conditions. Yet, we demonstrated that a simplistic and flexible strategy, e.g., the use of a metal cofactor, an approach frequently utilized in structural biology studies, could be employed to increase the stability, and therefore the detectability, of the GroEL complexes.

Defects of the cellular folding machinery can cause the formation of toxic protein aggregates and may be associated with devastating pathologies, including neurodegenerative and oncological diseases in humans. Such defects often involve heat shock proteins, e.g., Hsp70 and Hsp90, which are essential molecular chaperones that also exhibit conformational changes induced by ATP binding and hydrolysis. The developed CE–MS method could help investigate the interactions between potential drug candidates and molecular chaperones, as well as other biologically critical proteins, for the detection, investigation, monitoring, and treatment of human pathologies, using minute sample amounts. The developed CE–MS technique has the potential for studying non‐covalent and covalent interactions of a large variety of multimeric protein assemblies with different binding partners (ligands, proteins, metal cofactors), and for providing crucial and much‐needed information on the binding stoichiometry and kinetics of the analyzed ligand/protein–protein complexes (i.e., determination of association/dissociation constants) under native and near‐native conditions. The developed CE–MS method could also be integrated into a top–down proteomics platform and provide invaluable information on the sequence, post‐translational modifications, and metal cofactor/ligand‐binding sites of macromolecular protein complexes, and provide new insights into various biological processes, including catalytic activity and protein folding. Finally, our method could have broad applicability if integrated into a vast analytical platform combining biophysical/biochemical techniques, including electron microscopy and computational modeling of proteins’ tertiary and quaternary structures for a better understanding of the structures and functions of complex biological systems.

## 4 Experimental Section

### Materials and Chemicals

Deionized water, methanol (99.9%), MgCl_2_, and beryllium sulfate (99.99%) were obtained from Fisher Scientific (Ward Hill, MA). 1 n NaOH, 1 n HCl, ultrahigh purity ammonium acetate (99.999%), Tris‐HCl, KCl, GuHCl, DTT, adenosine 5′‐O‐(3‐thiotriphosphate) tetralithium salt, and aluminum fluoride (99.8%) were purchased from Sigma–Aldrich (St. Louis, MO). All sheathless bare fused silica (BFS) OptiMS CESI capillaries (91 cm × 30 µm i.d. × 150 µm o.d.) were from SCIEX (Brea, CA).

### Protein Expression and Purification

All proteins were expressed in *E. coli* BL21 (DE3) Gold (Stratagene, La Jolla, CA) strain and purified from the soluble fraction as previously described in. ref. [5] The double‐ring (DR) and single‐ring (SR) GroEL mutants were generated by QuikChange mutagenesis (Stratagene) from the WT GroEL gene. The four mutations (Cys138Ala, Cys458Ala, Cys519Ala, and Met548Cys) and the four mutations (Arg452Glu, Glu461Ala, Ser463Ala, and Val464Ala) introduced in the DR and SR mutants, respectively, were confirmed by DNA sequencing. Cells were grown at 20 °C to an optical density (OD) of 0.4–0.6 absorbance units, and protein expression was induced by the addition of 1 mm isopropyl *β*‐D‐1‐thiogalactopyranoside (IPTG). After 4–6 h at 20 °C, the cells were harvested by centrifugation (4000× g, 40 min, 4 °C), resuspended in 100 mm Tris‐HCl pH 7.5, 10 mm DTT, and subsequently frozen in liquid nitrogen. Thawed cells were incubated for 1 h at 4 °C in the presence of 1 mg L^−1^ lysozyme (Sigma–Aldrich) and 10 U L^−1^ benzonase (Merck, Darmstadt, Germany), and subsequently lysed on ice by sonication. All purification steps for the individual proteins (detailed in ref. [5]) were performed at 4 °C. Pre‐purified proteins were individually subjected to further purification using size exclusion chromatography (Sephacryl S300, 26/60 column (Cytiva, Marlborough, MA) equilibrated with 30 mm Tris‐HCl pH 7.5, 100 mm KCl, 1 mm EDTA) to remove aggregates and incompletely assembled complexes. Purified proteins were concentrated using Vivaspin 10 kDa MWCO ultrafiltration devices and frozen in liquid nitrogen in presence of 5% glycerol. To ensure the proteins were fully active, functional tests were performed as described in. ref. [5] The ATPase activity of WT and DR mutant GroEL in the presence and absence of GroES was measured using a coupled enzymatic assay. Enzyme activity was measured for DapA and NanA, and the protein concentration was determined spectrophotometrically at 280 nm. In this study, chaperonin concentrations refer to the oligomeric state, while SP concentrations refer to the monomeric state.

### GroEL and GroES Purification

GroEL and GroES chaperones were purified as previously described in refs. [5, 80, 81] Briefly, after the removal of cell debris and membranes by centrifugation at 4 °C (20 min at 48 000× g), the supernatant was adjusted to 20 mg mL^−1^ in 30 mm Tris pH 7.8, 50 mm NaCl, 1 mm EDTA, 1 mm DTT, and fractionated by successive steps of chromatography on DE52 weak anion exchange resin in the same buffer, DE52 in 25 mm histidine pH 5.5, 50 mm NaCl, 1 mm DTT, phenyl–Sepharose CL‐4B (Cytiva) in 20 mm MOPS pH 7.2, 400 mm NaCl, 1 mm DTT, and Sephacryl S300‐HR (Cytiva) in 10 mm MOPS pH 7.2, 50 mm KCl, 1 mm DTT. GroEL‐ and GroES‐containing fractions were pooled.

### Molecular Mass Determination

The theoretical relative molecular mass (Mr_th_) of the WT GroEL 14‐mer (GroEL_14_) was calculated based on the amino acid sequence of the WT GroEL monomer (Figure S1A, Supporting Information). The Mr_th_ of the unmodified WT GroEL_14_ (i.e., absence of N‐terminal methionine cleavage) and the Mr_th_ of the truncated WT GroEL_14_ (i.e., 14 N‐terminal methionine cleaved monomers) were 802 603.90 and 800 767.13 Da, respectively. The Mr_th_ of the mutant DR GroEL 14‐mer (GroEL_14_) was calculated based on the amino acid sequence of the WT GroEL monomer, where Cys138, Cys458, and Cys519 were mutated to Ala, and Met548 was mutated to Cys (Figure S1B, Supporting Information). The Mr_th_ of the unmodified and truncated mutant DR GroEL_14_ were 800 864.54 and 799 027.77 Da, respectively. The Mr_th_ of the mutant SR GroEL 7‐mer (GroEL_7_) was calculated based on the amino acid sequence of the WT GroEL monomer, where Arg452 was mutated to Glu, and Glu461, Ser463, and Val464 were mutated to Ala (Figure S1B, Supporting Information). The Mr_th_ of the unmodified and truncated mutant SR GroEL_7_ were 400 397.83 and 399 479.45 Da, respectively. The Mr_th_ of the WT GroES 7‐mer (GroES_7_) was calculated based on the amino acid sequence of the WT GroES monomer (Figure S1C, Supporting Information). The Mr_th_ of the unmodified and truncated GroES_7_ were 72 708.65 and 71 790.27 Da, respectively. The Mr_th_ of the WT NanA 4‐mer (NanA_4_) was calculated based on the amino acid sequence of the WT NanA monomer (Figure S1D, Supporting Information). The Mr_th_ of the unmodified and truncated NanA_4_ were 130 373.88 and 129 849.09 Da, respectively. The Mr_th_ of the WT DapA 4‐mer (DapA_4_) was calculated based on the amino acid sequence of the WT DapA monomer (Figure S1E, Supporting Information). The Mr_th_ of the unmodified and truncated DapA_4_ were 125 079.88 and 124 555.09 Da, respectively. The experimental molecular masses (Mr_exp_) of the detected GroEL complexes were calculated with three replicate CE–MS analyses.

Based on the computationally predicted 3D structures (see below) and previously reported works,^[^ ^50^, ^52^ ^]^ the molecular masses of GroEL_14_‐(ATPγS)_n_ (where *n* = 1–14), GroEL_14_–(ATPγS)_7_–GroES_7_, GroEL_14_–(ADP)_3_, and GroEL_14_–(ADP)_7_–GroES_7_ were calculated considering that each ATPγS or ADP nucleotide molecule bound to GroEL was coordinated to one Mg^2+^ (24.31 Da) and one K^+^ (39.10 Da) ions. When the metal cofactors BeF_3_ and AlF_3_ were used, the molecular masses of one Be^2+^ and three F^−^ ions (i.e., 66.01 Da) or one Al^3+^ and three F^−^ ions (i.e., 83.98 Da) were added at a molecular ratio metal cofactor: nucleotide (1:1) (Table S1, Supporting Information).

### CE–MS Methods

CE–MS experiments were conducted using a CESI 8000 instrument (SCIEX, Brea, CA). For all experiments, bare fused silica (BFS) OptiMS capillaries (91 cm × 30 µm i.d. × 150 µm o.d.) were used. Conditioning of the BFS capillaries was performed with a series of rinses (100 psi, 10 min) with MeOH, 0.1 m NaOH, 0.1 m HCl, and Milli‐Q water. Prior to each injection, a series of rinses of the separation and conductive lines were performed. For the separation capillary, these rinses included: 0.1 m HCl (100 psi, 3 min), Milli‐Q water (100 psi, 5 min), followed by the background electrolyte (BGE) (100 psi, 7 min). The conductive line was rinsed with the BGE (100 psi, 2 min). The cartridge and sample tray temperatures were 15 and 10 °C, respectively. All CE methods employed 20 kV in reverse polarity with a voltage ramp time set to 1 min. A supplemental pressure of 10 psi was applied at the inlet of the CE capillary in all experiments to generate a highly stable nano‐electrospray, counteract the cathodic EOF (Figure S2, Supporting Information), and shorten the analysis time, while preserving the CE resolution in the separation of the GroEL complexes (applying a supplemental pressure of 5–9 psi, lower spray stability and reproducibility of migration times and peak areas were observed). Prior to CE–MS analysis, all the protein complexes (chaperones and SPs) were diluted in a refolding buffer consisting of 50 mm Tris‐HCl pH 7.5, 50 mm KCl, and 15 mm MgCl_2_. Prior to their incubation with GroEL_14_, the oligomeric SPs (NanA or DapA tetramers) were denatured with guanidine hydrochloride (GuHCl) and dithiothreitol (DTT). For this denaturation step, 200 to 400 µm of SP were mixed with 6.5 m GuHCl and 9 mm DTT, pH 8.0. Then the denatured SPs were diluted 30‐fold in the GroEL_14_‐containing buffer at pH 7.5. Finally, the SP‐GroEL_14_ samples prepared for CE–MS analysis contained 6 µm of GroEL_14_, 6 or 12 µm of SP monomers, 200 mm GuHCl, and 0.3 mm DTT, pH 7.5. Sample injections were performed at 1 psi for 6 to 60 s (corresponding to 1 and 10 nL injection volumes, respectively, i.e., 0.2% and 1.6% of the capillary volume), depending on the BGE composition. The experiments were carried out with a BGE of 50 mm ammonium acetate pH 6.7 with or without ATPγS or ADP. For the analysis of GroEL_14_‐nucleotide and GroEL_14_–GroES_7_ complexes, different amounts of ATPγS or ADP (10 to 50 µm) were added to the BGE. Two different stock solutions of 100 mm ATPγS (or ADP) were prepared: one stock solution was prepared with UHPLC‐MS grade water (Thermo Scientific), whereas the other stock solution was prepared with a refolding buffer. Unless stated otherwise, all the experiments dedicated to the analysis of GroEL_14_‐nucleotide and GroEL_14_‐GroES_7_ complexes were performed with a BGE prepared with the nucleotide stock solution in a refolding buffer. In these experiments, the concentration of Mg^2+^ ions in the BGE varied from 15 to 75 µm, depending on the nucleotide concentration added to the BGE. Unless stated otherwise, the GroEL_14_ complexes were injected at 6 µm (injected quantity 6 fmol). For the experiments using the metal cofactors AlF_3_ and BeF_3_, GroEL_14_ (6 µm), GroES_7_ (6 µm), and the nucleotide (ATPγS or ADP, 1 mm) were incubated with the metal cofactors AlF_3_ (1 mm) or BeF_3_ (1 mm, generated by combing 1 mm BeSO_4_ and 10 mm KF) on average 20 min before their injection into the CE capillary.

### MS Instrumentation

Q Exactive Plus–UHMR Orbitrap MS (Thermo Fisher Scientific, Bremen, Germany) was used for all experiments. All CE‐single‐stage MS and CE‐tandem MS analyses were carried out in positive ESI mode using a Nanospray Flex source (Thermo Fisher Scientific) and an OptiMS adapter (SCIEX) to establish the mechanical and electrical interface between the CESI OptiMS cartridges and the Thermo Nanospray flex source. The temperature of the ion transfer tube (ITT) at the entrance into the MS was set to 250 °C. The distance between the nano‐electrospray emitter and the ITT was ≈10–12 mm, and the nano‐electrospray potential was set to +2.2 kV. Using these conditions, no electrical arcing phenomena were observed and high electrospray stability and optimal MS signal intensity levels were achieved, while improving the MS signal quality due to the emitter geometry, selected BGE, and the distance between the electrospray emitter and the ITT. The CE–MS^1^ analyses were performed with a trapping gas set to 6, an in‐source‐desolvation (ISD) voltage of −100 V, an automatic gain control (AGC) of 10^6^, a maximum injection time of 200 ms, 10 microscans, an S‐lens RF level set to 200, and, unless stated otherwise, the nominal resolution of 3125 at 200 *m/z*. The scan range was *m/z* 2000–30 000. For CE‐pseudo‐MS^3^ experiments, all ion fragmentation (AIF) mode was used. The CE‐pseudo‐MS^3^ analyses were performed with a trapping gas set to 1, a maximum injection time of 1000 ms, 10 microscans, and the nominal resolution of 100 000 at 200 *m/z*. To eject the monomeric subunits of GroEL in order to select and isolate them in the quadrupole mass filter for their subsequent fragmentation in the higher energy collision dissociation (HCD) cell, ISD and in‐source collision‐induced dissociation (ISCID) voltages of −100 V and +100 eV were applied, respectively. The fragmentation of the GroEL monomeric subunit ions was performed applying an HCD energy of 160 eV in the HCD cell. The scan range was set to *m/z* 350–6000, and the isolation range was set to *m/z* 3513–4113. Other parameters were as described above for CE–MS^1^ analyses. For nanoESI‐MS‐based direct infusion experiments, 9 µL of an isolate of 34 µm GroEL_14_ in 200 mm ammonium acetate pH 6.7 were infused manually using metal‐coated pulled glass NanoESI emitters (Protana, Odense, Denmark), with a tip internal diameter of 1–4 µm. To generate and maintain a stable nano‐electrospray, a voltage was applied on the metal coating of the NanoESI emitter, while manually applying a constant backpressure with an air‐filled syringe. The flow rate was, therefore, mainly dictated by the electrospray process. The NanoESI emitters were placed ≈4–5 mm away from the ITT, and the spray voltage was set to +1.4 kV. The MS parameters were as depicted above for CE–MS^1^ analyses. For CE‐nanoESI‐MS‐based direct infusion experiments, one isolate of 6 µm GroEL_14_ in 40 mm ammonium acetate pH 6.7 was infused through the CE capillary and the CESI sheathless interface by applying a continuous pressure of 5 psi. The CE–MS parameters were as described above for CE–MS^1^ analyses.

### Data Analysis

For data acquisition and processing, Xcalibur (v. 4.1.31.9) software was used. The signal‐to‐noise (S/N) ratios were measured by integrating the MS signal across CE–MS peaks in the base peak electropherograms (BPEs) recorded in the CE–MS analyses of GroEL_14_ using Xcalibur software. A manual evaluation of the S/N ratios was also performed by averaging the S/N ratios of the most intense peak of the charge state distribution selecting multiple scans, and confirmed the results generated by the Xcalibur software. CE–MS data were processed with BioPharma Finder (v. 4.0). Deconvolution was performed using the sliding window approach with the ReSpect algorithm for isotopically unresolved data. For the deconvolution of the mass spectra of WT and mutant GroEL, the following main parameters were set: output mass range: 790 000–810 000; deconvolution mass tolerance: 30 ppm; charge state range: 60–75; minimum adjacent charges: 6–10; target mass: 800 000; charge carrier: H; and peak detection quality measure: 95%. CE‐pseudo‐MS^3^ data were processed with BioPharma Finder (v. 4.0) and ClipsMS (v. 2.0.0).^[^ ^74^ ^]^ BioPharma Finder was used for the search of N‐ and C‐terminal fragment ions, and the main parameters were as follows: *m/z* range 350–6000; output mass range 50–60 000; S/N threshold 3; charge range 1–20; fragmentation mass tolerance 10 ppm; fit factor 70%; and the deconvolution algorithm was Xtract for isotopically resolved MS data. Sodium and potassium adducts were included in the search. For the assignment of internal fragments, ClipsMS (v. 2.0.0)^[^ ^74^ ^]^ was used with the following search parameters: terminal fragment and internal fragment error tolerances were set to 10 and 1 ppm, respectively. The minimum internal fragment length was restricted to five amino acid residues. The identified *b‐*, *y‐*, and *by*‐ions had to be detected in the fragmentation spectra acquired during the CE migration of the corresponding complex of interest. In addition, the BPEs recorded in CE‐pseudo‐MS^3^ analyses were integrated across a narrow CE migration time window (0.2–0.5 min), corresponding to only 2–5 pseudo‐MS^3^ spectra, in the search of *b‐*, *y‐*, and *by*‐ions. Processing and generation of theoretical fragments and mass matching assessment were performed based on the WT and mutant GroEL amino acid sequences (with truncated N‐terminal methionine) and the mass of an H+ proton (1.00728 amu). No modifications were included in the search for *b*‐, *y*‐, and *by*‐type ions corresponding to HCD fragmentation. As additional verification of the reliability of the internal fragments identified with ClipsMS software, a manual data examination was conducted, which consisted of the search for possible matches between the identified terminal and internal fragments. N‐ and C‐terminal amino acid residues of the detected *by*‐ions were required to match the C‐ and N‐termini of the corresponding and complementary *b‐* or *y‐*fragment ions, from which the internal ions expectedly derived. The output of ClipsMS algorithm was parsed and combined with BioPharma Finder (v. 4.0) output using custom Python scripts and plots were generated using R. The sequence coverage of the proteins was calculated as the number of identified inter‐amino acid residue cleavage sites divided by the total number of inter‐residue bonds. Manual examination of the acquired mass spectra was performed for all the detected protein complexes based on the theoretical molecular masses indicated in Table S1 (Supporting Information). When the CE–MS signal intensity levels were above 1E4, a strong agreement was noticed between the experimental molecular masses determined manually and those generated by data processing using the ReSpect™ deconvolution algorithm in BioPharma Finder (v. 4.0). For the CE–MS signal intensity levels below 1E4, a manual examination had to be performed systematically due to the misassignment of the ion charge states with BioPharma Finder software. The migration times and the quantification of the detected GroEL complexes were determined with three replicate CE–MS analyses.

Adobe Illustrator was used to draw the protein folding cycle depicted in Figure 1. The 3D structures of WT and mutant GroEL monomers were modeled with the Python version of the PyMOL software^[^ ^82^ ^]^ based on the UniProtKB accession number P0A6F5 and the AlphaFold^[^ ^75^ ^]^ computationally predicted structure. The 3D structures of mutant GroEL_14_, mutant GroEL_14_–(ATPγS)_14_, and GroES_7_ were modeled with PyMOL based on the PDB entries 1OEL, 1SX3, and 2C7C, respectively.^[^ ^50^, ^83^, ^84^ ^]^ In PyMOL, the wizard mutagenesis option was used to introduce the selected mutations in the 3D structures. SWISS‐MODEL fully automated server (https://swissmodel.expasy.org)^[^ ^85^ ^]^ was used to model GroEL_14_ (WT and mutants) and NanA_4_ tetramer based on the amino acid sequences of the respective proteins. In SWISS‐MODEL, the protein secondary structure motifs were annotated based on DSSP^[^ ^86^ ^]^ and PSIPRED,^[^ ^87^ ^]^ using the following letters: H, *α*‐helix; G, 3_10_‐helix; E, extended strand that participates in *β*‐ladder; B, residue in isolated *β*‐bridge; S, bend; C, loop; T, hydrogen‐bonded turn. The dimensions of mutant GroEL_14_, mutant GroEL_14_–(ATPγS)_14_, and mutant GroEL_14_–(ATPγS)_7_–GroES_7_ complexes were assessed based on the measurement of the computationally designed 3D structures and previously reported studies.^[^ ^56^, ^57^, ^58^, ^64^ ^]^ The computational modeling of the protein 3D structures included the first methionine residue at the N‐termini of the monomeric subunits. Therefore, a shift of +1 in the amino acid numbering is systematically observed compared to the numbering of *b* and *y* terminal and *by* internal fragment ions.

### Statistical Analysis

All CE–MS experiments were independently performed at least three times, and the data were shown as mean values ± standard deviation.

## Conflict of Interest

The authors declare no conflict of interest.

## Author Contributions

A.L.M. developed the CE–MS methods, performed the experiments, processed and interpreted the data, and designed the 3D structures of the GroEL complexes with molecular graphics software. F.G. overexpressed and purified the proteins and contributed to designing the study and data interpretation. K.R.J. conducted the initial CE–MS experiments and contributed to CE–MS method development. S.R. contributed to the data processing. J.R.E. contributed to discussions and interpretation of results. A.L.M. and A.R.I. designed the study, evaluated and interpreted the results, and wrote the manuscript. All co‐authors read and commented on the manuscript and supplementary information.

## Supporting information

Supporting Information

## Data Availability

All data were deposited in MassIVE MSV000091656.

## References

[1] Y.‐C. Tang , H.‐C. Chang , A. Roeben , D. Wischnewski , N. Wischnewski , M. J. Kerner , F. U. Hartl , M. Hayer‐Hartl , Cell 2006, 125, 903. 16751100 10.1016/j.cell.2006.04.027

[2] D. K. Clare , H. R. Saibil , Biopolymers 2013, 99, 846. 23877967 10.1002/bip.22361 PMC3814418

[3] O. Genest , S. Wickner , S. M. Doyle , J. Biol. Chem. 2019, 294, 2109. 30401745 10.1074/jbc.REV118.002806 PMC6369297

[4] H. Taguchi , J. Biochem. 2005, 137, 543. 15944406 10.1093/jb/mvi069

[5] F. Georgescauld , K. Popova , A. J. Gupta , A. Bracher , J. R. Engen , M. Hayer‐Hartl , F. U. Hartl , Cell 2014, 157, 922. 24813614 10.1016/j.cell.2014.03.038 PMC4071350

[6] M. Hayer‐Hartl , A. Bracher , F. U. Hartl , Trends Biochem. Sci. 2016, 41, 62. 26422689 10.1016/j.tibs.2015.07.009

[7] M. W. Jaworek , S. Möbitz , M. Gao , R. Winter , Phys. Chem. Chem. Phys. 2020, 22, 3734. 32010904 10.1039/c9cp06468k

[8] A. Goyon , S. Fekete , A. Beck , J.‐L. Veuthey , D. Guillarme , J. Chromatogr. B Analyt. Technol. Biomed. Life Sci. 2018, 1092, 368. 10.1016/j.jchromb.2018.06.029 29936373

[9] G. Sakashita , H. Kiyoi , T. Naoe , T. Urano , Sci. Rep. 2018, 8, 4008. 29507312 10.1038/s41598-018-22359-w PMC5838202

[10] G. Rouby , N. T. Tran , Y. Leblanc , M. Taverna , N. Bihoreau , MAbs 2020, 12, e1781743. 32633190 10.1080/19420862.2020.1781743 PMC7531515

[11] T. Wyttenbach , N. A. Pierson , D. E. Clemmer , M. T. Bowers , Annu. Rev. Phys. Chem. 2014, 65, 175. 24328447 10.1146/annurev-physchem-040513-103644

[12] H. Wang , J. Eschweiler , W. Cui , H. Zhang , C. Frieden , B. T. Ruotolo , M. L. Gross , J. Am. Soc. Mass Spectrom. 2019, 30, 876. 30887458 10.1007/s13361-019-02148-z PMC6504607

[13] P. Pathak , A. A. Shvartsburg , Anal. Chem. 2020, 92, 13855. 32886883 10.1021/acs.analchem.0c02551

[14] C. S. Jørgensen , L. R. Ryder , A. Steinø , P. Højrup , J. Hansen , N. H. Beyer , N. H. H. Heegaard , G. Houen , Eur. J. Biochem. 2003, 270, 4140. 14519126 10.1046/j.1432-1033.2003.03808.x

[15] A.‐L. Marie , N. T. Tran , F. Saller , Y. M. Abdou , P. Zeau , J.‐L. Plantier , R. Urbain , D. Borgel , M. Taverna , Electrophoresis 2016, 37, 1696. 26989842 10.1002/elps.201500456

[16] R. L. C. Voeten , I. K. Ventouri , R. Haselberg , G. W. Somsen , Anal. Chem. 2018, 90, 1464. 29298038 10.1021/acs.analchem.8b00015 PMC5994730

[17] P. G. Righetti , B. Verzola , Electrophoresis 2001, 22, 2359. 11519938 10.1002/1522-2683(200107)22:12<2359::AID-ELPS2359>3.0.CO;2-8

[18] H. Stutz , M. Wallner , H. Malissa , G. Bordin , A. R. Rodriguez , Electrophoresis 2005, 26, 1089. 15719362 10.1002/elps.200406195

[19] L. Bertoletti , F. Bisceglia , R. Colombo , S. Giorgetti , S. Raimondi , P. P. Mangione , E. De Lorenzi , Electrophoresis 2015, 36, 2465. 26084573 10.1002/elps.201500148

[20] S. N. Krylov , J. Biomol. Screen 2006, 11, 115. 16418314 10.1177/1087057105284339

[21] M. Shanmuganathan , P. Britz‐Mckibbin , Anal. Chim. Acta 2013, 773, 24. 23561903 10.1016/j.aca.2013.01.061

[22] A.‐L. Marie , N. T. Tran , E. P. Bianchini , F. Saller , S. Pautus , T. Abache , J.‐L. Plantier , R. Urbain , D. Borgel , M. Taverna , J. Pharm. Biomed. Anal. 2015, 111, 64. 25863018 10.1016/j.jpba.2015.02.042

[23] F. Yu , Q. Zhao , D. Zhang , Z. Yuan , H. Wang , Anal. Chem. 2019, 91, 372. 30392351 10.1021/acs.analchem.8b04741

[24] Y. Kawata , K. Hongo , K. Nosaka , Y. Furutsu , T. Mizobata , J. Nagai , FEBS Lett. 1995, 369, 283. 7649273 10.1016/0014-5793(95)00768-5

[25] A.‐L. Marie , E. Dominguez‐Vega , F. Saller , J.‐L. Plantier , R. Urbain , D. Borgel , N. T. Tran , G. W. Somsen , M. Taverna , Anal. Chim. Acta 2016, 947, 58. 27846990 10.1016/j.aca.2016.10.016

[26] L. Bertoletti , J. Schappler , R. Colombo , S. Rudaz , R. Haselberg , E. Domínguez‐Vega , S. Raimondi , G. W. Somsen , E. De Lorenzi , Anal. Chim. Acta 2016, 945, 102. 27968711 10.1016/j.aca.2016.10.010

[27] E. H. Domínguez‐Vega , R. Haselberg , G. W. Somsen , Capillary Zone Electrophoresis–Mass Spectrometry of Intact Proteins, Humana Press, New York, NY 2016. 10.1007/978-1-4939-4014-1_3 27473479

[28] F. P. Gomes , J. R. Yates 3rd , Mass Spectrom Rev 2019, 38, 445. 31407381 10.1002/mas.21599 PMC6800771

[29] Y. N. Francois , A. L. Marie , C. Ruel , R. Gahoual , N. T. Tran , M. Taverna , Advances in Chromatography, CRC Press, Boca Raton 2019.

[30] A. Nguyen , M. Moini , Anal. Chem. 2008, 80, 7169. 18710259 10.1021/ac801158q

[31] C. Przybylski , M. Mokaddem , M. Prull‐Janssen , E. Saesen , H. Lortat‐Jacob , F. Gonnet , A. Varenne , R. Daniel , Analyst 2015, 140, 543. 25408953 10.1039/c4an01305k

[32] Y.‐N. François , M. Biacchi , N. Said , C. Renard , A. Beck , R. Gahoual , E. Leize‐Wagner , Anal. Chim. Acta 2016, 908, 168. 26826699 10.1016/j.aca.2015.12.033

[33] A. M. Belov , R. Viner , M. R. Santos , D. M. Horn , M. Bern , B. L. Karger , A. R. Ivanov , J. Am. Soc. Mass Spectrom. 2017, 28, 2614. 28875426 10.1007/s13361-017-1781-1 PMC5709234

[34] A. M. Belov , L. Zang , R. Sebastiano , M. R. Santos , D. R. Bush , B. L. Karger , A. R. Ivanov , Electrophoresis 2018, 39, 2069. 29749064 10.1002/elps.201800067 PMC6119659

[35] V. Le‐Minh , N. T. Tran , A. Makky , V. Rosilio , M. Taverna , C. Smadja , J. Chromatogr. A 2019, 1601, 375. 31160095 10.1016/j.chroma.2019.05.050

[36] M. R. Mehaffey , Q. Xia , J. S. Brodbelt , Anal. Chem. 2020, 92, 15202. 33156608 10.1021/acs.analchem.0c03784 PMC7788560

[37] K. Vuignier , J.‐L. Veuthey , P.‐A. Carrupt , J. Schappler , Electrophoresis 2012, 33, 3306. 22949263 10.1002/elps.201200116

[38] C. M. Ouimet , M. Dawod , J. Grinias , V. A. Assimon , J. Lodge , A. K. Mapp , J. E. Gestwicki , R. T. Kennedy , Analyst 2018, 143, 1805. 29565056 10.1039/C7AN02098H PMC5902653

[39] H. Nevídalová , L. Michalcová , Z. Glatz , Electrophoresis 2019, 40, 625. 30600537 10.1002/elps.201800367

[40] J. L. P. Benesch , B. T. Ruotolo , D. A. Simmons , N. P. Barrera , N. Morgner , L. Wang , H. R. Saibil , C. V. Robinson , J. Struct. Biol. 2010, 172, 161. 20227505 10.1016/j.jsb.2010.03.004

[41] V. A. Mikhailov , T. H. Mize , J. L. P. Benesch , C. V. Robinson , Anal. Chem. 2014, 86, 8321. 25026391 10.1021/ac5018327

[42] E. Van Duijn , D. A. Simmons , R. H. H. Van Den Heuvel , P. J. Bakkes , H. Van Heerikhuizen , R. M. A. Heeren , C. V. Robinson , S. M. Van Der Vies , A. J. R. Heck , J. Am. Chem. Soc. 2006, 128, 4694. 16594706 10.1021/ja056756l

[43] E. V. Duijn , A. Barendregt , S. Synowsky , C. Versluis , A. J. R. Heck , J. Am. Chem. Soc. 2009, 131, 1452. 19138114 10.1021/ja8055134

[44] J.‐F. Greisch , S. A. M. Van Der Laarse , A. J. R. Heck , Anal. Chem. 2020, 92, 15506. 33180479 10.1021/acs.analchem.0c03412 PMC7711774

[45] J. F. Hevler , M. V. Lukassen , A. Cabrera‐Orefice , S. Arnold , M. F. Pronker , V. Franc , A. J. R. Heck , EMBO J. 2021, 40, 106174. 10.15252/embj.2020106174 PMC7883291 33459420

[46] M. E. Belov , E. Damoc , E. Denisov , P. D. Compton , S. Horning , A. A. Makarov , N. L. Kelleher , Anal. Chem. 2013, 85, 11163. 24237199 10.1021/ac4029328

[47] K. Jooß , J. P. McGee , R. D. Melani , N. L. Kelleher , Electrophoresis 2021, 42, 1050. 33502026 10.1002/elps.202000317 PMC8122066

[48] G. W. Farr , EMBO J. 2003, 22, 3220. 12839985 10.1093/emboj/cdg313 PMC165638

[49] P. T. Wingfield , Curr. Protoc. Protein Sci. 2015, 80, 35. 10.1002/0471140864.ps0601s80 PMC4410719 25829302

[50] C. Chaudhry , A. L. Horwich , A. T. Brunger , P. D. Adams , J. Mol. Biol. 2004, 342, 229. 15313620 10.1016/j.jmb.2004.07.015

[51] T. E. Walker , M. Shirzadeh , H. M. Sun , J. W. McCabe , A. Roth , Z. Moghadamchargari , D. E. Clemmer , A. Laganowsky , H. Rye , D. H. Russell , J. Am. Chem. Soc. 2022, 144, 2667. 35107280 10.1021/jacs.1c11341 PMC8939001

[52] S. S. Kudryavtseva , E. B. Pichkur , I. A. Yaroshevich , A. A. Mamchur , I. S. Panina , A. V. Moiseenko , O. S. Sokolova , V. I. Muronetz , T. B. Stanishneva‐Konovalova , Sci. Rep. 2021, 11, 18241. 34521893 10.1038/s41598-021-97657-x PMC8440773

[53] R. Gruber , A. Horovitz , Chem. Rev. 2016, 116, 6588. 26726755 10.1021/acs.chemrev.5b00556

[54] D. K. Clare , P. J. Bakkes , H. Van Heerikhuizen , S. M. Van Der Vies , H. R. Saibil , Nature 2009, 457, 107. 19122642 10.1038/nature07479 PMC2728927

[55] R. Natesh , D. K. Clare , G. W. Farr , A. L. Horwich , H. R. Saibil , Int. J. Biol. Macromol. 2018, 118, 671. 29959019 10.1016/j.ijbiomac.2018.06.120 PMC6096091

[56] M. Yokokawa , C. Wada , T. Ando , N. Sakai , A. Yagi , S. H. Yoshimura , K. Takeyasu , EMBO J. 2006, 25, 4567. 16977315 10.1038/sj.emboj.7601326 PMC1590003

[57] S. Chen , A. M. Roseman , A. S. Hunter , S. P. Wood , S. G. Burston , N. A. Ranson , A. R. Clarke , H. R. Saibil , Nature 1994, 371, 261. 7915827 10.1038/371261a0

[58] D. K. Clare , D. Vasishtan , S. Stagg , J. Quispe , G. W. Farr , M. Topf , A. L. Horwich , H. R. Saibil , Cell 2012, 149, 113. 22445172 10.1016/j.cell.2012.02.047 PMC3326522

[59] M. U. Musheev , Y. Filiptsev , V. Okhonin , S. N. Krylov , J. Am. Chem. Soc. 2010, 132, 13639. 20831170 10.1021/ja105754h

[60] D. C. Boisvert , J. Wang , Z. Otwinowski , A. L. Norwich , P. B. Sigler , Nat. Struct. Biol. 1996, 3, 170. 8564544 10.1038/nsb0296-170

[61] E. Chapman , G. W. Farr , W. A. Fenton , S. M. Johnson , A. L. Horwich , Proc. Natl. Acad. Sci. U S A 2008, 105, 19205. 19050077 10.1073/pnas.0810657105 PMC2592988

[62] A. Dyachenko , R. Gruber , L. Shimon , A. Horovitz , M. Sharon , Proc. Natl. Acad. Sci. U S A 2013, 110, 7235. 23589876 10.1073/pnas.1302395110 PMC3645570

[63] A. Horovitz , K. R. Willison , Curr. Opin. Struct. Biol. 2005, 15, 646. 16249079 10.1016/j.sbi.2005.10.001

[64] Z. Xu , A. L. Horwich , P. B. Sigler , Nature 1997, 388, 741. 9285585 10.1038/41944

[65] C. Chaudhry , EMBO J. 2003, 22, 4877. 14517228 10.1093/emboj/cdg477 PMC204461

[66] H. Taguchi , K. Tsukuda , F. Motojima , A. Koike‐Takeshita , M. Yoshida , J. Biol. Chem. 2004, 279, 45737. 15347650 10.1074/jbc.M406795200

[67] H. Taguchi , J. Mol. Biol. 2015, 427, 2912. 25900372 10.1016/j.jmb.2015.04.007

[68] X. Fei , X. Ye , N. A. Laronde , G. H. Lorimer , Proc. Natl. Acad. Sci. U S A 2014, 111, 12775. 25136110 10.1073/pnas.1412922111 PMC4156775

[69] S. Haldar , A. J. Gupta , X. Yan , G. Milicic , F. U. Hartl , M. Hayer‐Hartl , J. Mol. Biol. 2015, 427, 2244. 25912285 10.1016/j.jmb.2015.04.009

[70] H. S. Rye , A. M. Roseman , S. Chen , K. Furtak , W. A. Fenton , H. R. Saibil , A. L. Horwich , Cell 1999, 97, 325. 10319813 10.1016/s0092-8674(00)80742-4

[71] N. D. Schmitt , J. M. Berger , J. B. Conway , J. N. Agar , Anal. Chem. 2021, 93, 6355. 33844516 10.1021/acs.analchem.0c04670

[72] M. A. Zenaidee , B. Wei , C. Lantz , H. T. Wu , T. R. Lambeth , J. K. Diedrich , R. R. Ogorzalek Loo , R. R. Julian , J. A. Loo , J. Am. Soc. Mass Spectrom. 2021, 32, 1752. 34101447 10.1021/jasms.1c00113 PMC9090460

[73] Z. Rolfs , L. M. Smith , J. Proteome Res. 2021, 20, 5412. 34738820 10.1021/acs.jproteome.1c00599 PMC8790932

[74] C. Lantz , M. A. Zenaidee , B. Wei , Z. Hemminger , R. R. Ogorzalek Loo , J. A. Loo , J. Proteome Res. 2021, 20, 1928. 33650866 10.1021/acs.jproteome.0c00952 PMC8174100

[75] J. Jumper , R. Evans , A. Pritzel , T. Green , M. Figurnov , O. Ronneberger , K. Tunyasuvunakool , R. Bates , A. Zidek , A. Potapenko , A. Bridgland , C. Meyer , S. A. A. Kohl , A. J. Ballard , A. Cowie , B. Romera‐Paredes , S. Nikolov , R. Jain , J. Adler , T. Back , S. Petersen , D. Reiman , E. Clancy , M. Zielinski , M. Steinegger , M. Pacholska , T. Berghammer , S. Bodenstein , D. Silver , O. Vinyals , et al., Nature 2021, 596, 583. 34265844 10.1038/s41586-021-03819-2 PMC8371605

[76] M. S. Westphall , K. W. Lee , A. Z. Salome , J. M. Lodge , T. Grant , J. J. Coon , Nat. Commun. 2022, 13, 2276. 35478194 10.1038/s41467-022-29964-4 PMC9046196

[77] P. Fremdling , T. K. Esser , B. Saha , A. A. Makarov , K. L. Fort , M. Reinhardt‐Szyba , J. Gault , S. Rauschenbach , ACS Nano 2022, 16, 14443. 36037396 10.1021/acsnano.2c04831 PMC9527803

[78] H. Wu , R. Zhang , W. Zhang , J. Hong , Y. Xiang , W. Xu , Chem. Sci. 2020, 11, 4758. 34122932 10.1039/d0sc01965h PMC8159243

[79] M. He , P. Luo , J. Hong , X. Wang , H. Wu , R. Zhang , F. Qu , Y. Xiang , W. Xu , ACS Omega 2019, 4, 2377. 31459477 10.1021/acsomega.8b03224 PMC6648644

[80] M. J. Kerner , D. J. Naylor , Y. Ishihama , T. Maier , H.‐C. Chang , A. P. Stines , C. Georgopoulos , D. Frishman , M. Hayer‐Hartl , M. Mann , F. U. Hartl , Cell 2005, 122, 209. 16051146 10.1016/j.cell.2005.05.028

[81] M. K. Hayer‐Hartl , J. J. Ewbank , T. E. Creighton , F. U. Hartl , EMBO J. 1994, 13, 3192. 7913682 10.1002/j.1460-2075.1994.tb06618.x PMC395211

[82] S. Rosignoli , A. Paiardini , Biomolecules 2022, 1764, 12. 36551192 10.3390/biom12121764 PMC9775141

[83] K. Braig , P. D. Adams , A. T. Brünger , Nat. Struct. Biol. 1995, 2, 1083. 8846220 10.1038/nsb1295-1083

[84] N. A. Ranson , D. K. Clare , G. W. Farr , D. Houldershaw , A. L. Horwich , H. R. Saibil , Nat. Struct. Mol. Biol. 2006, 13, 147. 16429154 10.1038/nsmb1046 PMC2871290

[85] A. Waterhouse , M. Bertoni , S. Bienert , G. Studer , G. Tauriello , R. Gumienny , F. T. Heer , T. A. P. De Beer , C. Rempfer , L. Bordoli , R. Lepore , T. Schwede , Nucleic Acids Res. 2018, 46, W296. 29788355 10.1093/nar/gky427 PMC6030848

[86] W. Kabsch , C. Sander , Biopolymers 1983, 22, 2577. 6667333 10.1002/bip.360221211

[87] L. J. Mcguffin , K. Bryson , D. T. Jones , Bioinformatics 2000, 16, 404. 10869041 10.1093/bioinformatics/16.4.404

