# Supplementary material for: Native Capillary Electrophoresis–Mass Spectrometry of Near 1 MDa Non‐Covalent GroEL/GroES/Substrate Protein Complexes
Source: Adv Sci (Weinh). 2024 Jan 8;11(11):2306824. doi: 10.1002/advs.202306824 (PMC10953559; doi:10.1002/advs.202306824)
Supplement: Supplementary file 1 — Supporting Information [file ADVS-11-2306824-s001.pdf]

ADVANCED SCIENCE
Open Access

Supporting Information

for *Adv. Sci.*, DOI 10.1002/advs.202306824

Native Capillary Electrophoresis–Mass Spectrometry of Near 1 MDa Non-Covalent GroEL/GroES/Substrate Protein Complexes

*Anne-Lise Marie, Florian Georgescauld, Kendall R. Johnson, Somak Ray, John R. Engen and Alexander R. Ivanov**

Supplementary Information for

Native capillary electrophoresis-mass spectrometry of near 1 MDa non-covalent GroEL/GroES/substrate protein complexes

Anne-Lise Marie, Florian Georgescauld, Kendall R. Johnson, Somak Ray, John R. Engen, Alexander R. Ivanov*

* Corresponding author:
Tel: +1 617 373 6549
E-mail: a.ivanov@northeastern.edu

Figures S1-S19

Supplementary Note 1

Table S1

>sp|P0A6F5|CH60_ECOLI Chaperonin GroEL OS=Escherichia coli (strain K12) OX=83333 GN=groEL PE=1 SV=2

A)

MAAKDVKFGNDARVKMLRGVNVLADAVKVTLGPKGRNVVLDKSFGA
PTITKDGVSVAREIELEDKFENMGAQMVKEVASKANDAAGDGTTTAT
VLAQAIITEGLKAVAAGMNPMDLKRGIDKAVTAAVEELKALSVPCSDSK
AIAQVGTISANSDETVGKLIAEAMDKVGKEGVITVEDGTGLQDELDVV
EGMQFDRGYLSPYFINKPETGAVELESPFILLADKKISNIREMLPVLEAVA
KAGKPLLIIAEDVEGEALATLVVNTMRGIVKVAAVKAPGFGDRRKAML
QDIATLTGGTVISEEIGMELEKATLEDLGQAKRVVINKDTTTIIDGVGEEA
AIQGRVAQIRQQIEEATSDYDREKLQERVAKLAGGVAVIKVGAATEVEM
KEKKARVEDALHATRAAVEEGVVAGGGVALIRVASKLADLRGQNEDQ
NVGIKVALRAMEAPLRQIVLNCGEEPSVVANTVKGGDGNYGYNAATE
EYGNMIDMGILDPTKVTRSALQYAASVAGLMITTECMVTDLPKNDAA
DLGAAGGMGGMGGMGGMM

Figure S1: A) Amino acid sequence of the **wild-type (WT) double-ring (DR) GroEL$_{14}$** monomer (UniProtKB P0A6F5).

B)

```
DR mutant   MAAKDVKFGNDARVKMLRGVNVLADAVKVTLGPKGRNVVLDKSFGAPTITKDGVSVAREI 60
WT          MAAKDVKFGNDARVKMLRGVNVLADAVKVTLGPKGRNVVLDKSFGAPTITKDGVSVAREI 60
SR mutant   MAAKDVKFGNDARVKMLRGVNVLADAVKVTLGPKGRNVVLDKSFGAPTITKDGVSVAREI 60
            ************************************************************

DR mutant   ELEDKFENMGAQMVKEVASKANDAAGDGTTTATVLAQAIITEGLKAVAAGMNPMDLKRGI 120
WT          ELEDKFENMGAQMVKEVASKANDAAGDGTTTATVLAQAIITEGLKAVAAGMNPMDLKRGI 120
SR mutant   ELEDKFENMGAQMVKEVASKANDAAGDGTTTATVLAQAIITEGLKAVAAGMNPMDLKRGI 120
            ************************************************************

DR mutant   DKAVTAAVEELKALSVPASDSKAIAQVGTISANSDETVGKLIAEAMDKVGKEGVITVEDG 180
WT          DKAVTAAVEELKALSVPCSDSKAIAQVGTISANSDETVGKLIAEAMDKVGKEGVITVEDG 180
SR mutant   DKAVTAAVEELKALSVPCSDSKAIAQVGTISANSDETVGKLIAEAMDKVGKEGVITVEDG 180
            *****************.******************************************

DR mutant   TGLQDELDVVEGMQFDRGYLSPYFINKPETGAVELESPFILLADKKISNIREMLPVLEAV 240
WT          TGLQDELDVVEGMQFDRGYLSPYFINKPETGAVELESPFILLADKKISNIREMLPVLEAV 240
SR mutant   TGLQDELDVVEGMQFDRGYLSPYFINKPETGAVELESPFILLADKKISNIREMLPVLEAV 240
            ************************************************************

DR mutant   AKAGKPLLIIAEDVEGEALATLVVNTMRGIVKVAAVKAPGFGDRRKAMLQDIATLTGGTV 300
WT          AKAGKPLLIIAEDVEGEALATLVVNTMRGIVKVAAVKAPGFGDRRKAMLQDIATLTGGTV 300
SR mutant   AKAGKPLLIIAEDVEGEALATLVVNTMRGIVKVAAVKAPGFGDRRKAMLQDIATLTGGTV 300
            ************************************************************

DR mutant   ISEEIGMELEKATLEDLGQAKRVVINKDTTTIIDGVGEEAAIQGRVAQIRQQIEEATSDY 360
WT          ISEEIGMELEKATLEDLGQAKRVVINKDTTTIIDGVGEEAAIQGRVAQIRQQIEEATSDY 360
SR mutant   ISEEIGMELEKATLEDLGQAKRVVINKDTTTIIDGVGEEAAIQGRVAQIRQQIEEATSDY 360
            ************************************************************

DR mutant   DREKLQERVAKLAGGVAVIKVGAATEVEMKEKKARVEDALHATRAAVEEGVVAGGGVALI 420
WT          DREKLQERVAKLAGGVAVIKVGAATEVEMKEKKARVEDALHATRAAVEEGVVAGGGVALI 420
SR mutant   DREKLQERVAKLAGGVAVIKVGAATEVEMKEKKARVEDALHATRAAVEEGVVAGGGVALI 420
            ************************************************************

DR mutant   RVASKLADLRGQNEDQNVGIKVALRAMEAPLRQIVLNAGEEPSVVANTVKGGDGNYGYNA 480
WT          RVASKLADLRGQNEDQNVGIKVALRAMEAPLRQIVLNCGEEPSVVANTVKGGDGNYGYNA 480
SR mutant   RVASKLADLRGQNEDQNVGIKVALRAMEAPLEQIVLNCGEAPAAVANTVKGGDGNYGYNA 480
            *******************************.*****.** *:.****************

DR mutant   ATEEYGNMIDMGILDPTKVTRSALQYAASVAGLMITTEAMVTDLPKNDAADLGAAGGMGG 540
WT          ATEEYGNMIDMGILDPTKVTRSALQYAASVAGLMITTECMVTDLPKNDAADLGAAGGMGG 540
SR mutant   ATEEYGNMIDMGILDPTKVTRSALQYAASVAGLMITTECMVTDLPKNDAADLGAAGGMGG 540
            **************************************.*********************

DR mutant   MGGMGGMC      548
WT          MGGMGGMM      548
SR mutant   MGGMGGMM      548
            *******
```

Figure S1: B) Amino acid sequence alignment of the monomeric subunits of the **DR WT $GroEL_{14}$, DR mutant $GroEL_{14}$, and single-ring (SR) mutant $GroEL_{7}$**, generated by the open-access version of Clustal Omega™ software (an asterisk (*) indicates positions which have a single, fully conserved amino acid residue, a period (.) indicates conservation between groups of weakly similar properties, and a colon (:) indicates conservation between groups of strongly similar properties).

C)

>sp|P0A6F9|CH10_ECOLI Co-chaperonin GroES OS=Escherichia coli (strain K12) OX=83333 GN=groES PE=1 SV=1

MNIRPLHDRVIVKRKEVETKSAGGIVLTGSAAAKSTRGEVLAVGNGRIL
ENGEVKPLDVKVGDIVIFNDGYGVKSEKIDNEEVLIMSESDILAIVEA

D)

>sp|P0A6L4|NANA_ECOLI N-acetylneuraminate lyase OS=Escherichia coli (strain K12) OX=83333 GN=nanA PE=1 SV=2

MATNLRGVMAALLTPFDQQQALDKASLRRLVQFNIQQGIDGLYVGGST
GEAFVQSLSEREQVLEIVAEEAKGKIKLIAHVGCVSTAESQQLAASAKRYG
FDAVSAVTPFYYPFSFEEHCDHYRAIIDSADGLPMVVYNIPALSGVKLTLD
QINTLVTLPGVGALKQTSGDLYQMEQIRREHPDLVLYNGYDEIFASGLLA
GADGGIGSTYNIMGWRYQGIVKALKEGDIQTAQKLQTECNKVIDLLIKT
GVFRGLKTVLHYMDVVSVPLCRKPFGPVDEKYLPELKALAQQLMQERG

E)

>sp|P0A6L2|DAPA_ECOLI 4-hydroxy-tetrahydrodipicolinate synthase OS=Escherichia coli (strain K12) OX=83333 GN=dapA PE=1 SV=1

MFTGSIVAIVTPMDEKGNVCRASLKKLIDYHVASGTSAIVSVGTTGESATLN
HDEHADVVMMTLDLADGRIPVIAGTGANATAEAISLTQRFNDSGIVGCLTV
TPYYNRPSQEGLYQHFKAIAEHTDLPQILYNVPSRTGCDLLPETVGRLAKVKN
IIGIKEATGNLTRVNQIKELVSDDFVLLSGDDASALDFMQLGGHGVISVTANV
AARDMAQMCKLAAEGHFAEARVINQRLMPLHNKLFVEPNPIPVKWACKE
LGLVATDTLRLPMTPITDSGRETVRAALKHAGLL

Figure S1: Amino acid sequences of the monomeric subunits of C) **WT GroES$_7$** (UniProtKB P0A6F9), D) **WT NanA$_4$** (UniProtKB P0A6L4), and E) **WT DapA$_4$** (UniProtKB P0A6L2).

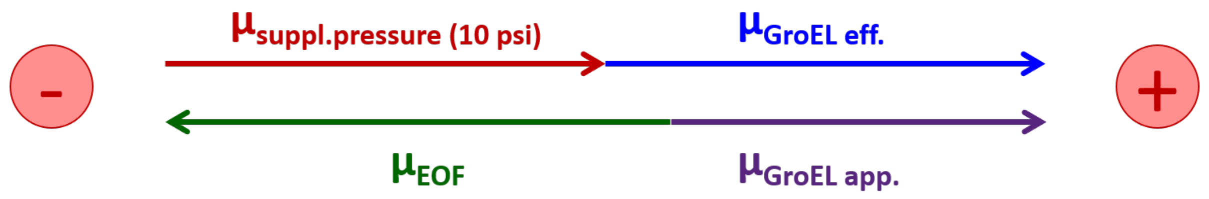

Figure S2: Schematic representation of the CE-MS analysis of GroEL complexes under our optimized CE-MS conditions. A supplemental CE pressure of 10 psi creates a flow rate of ~87 nL/min and was determined to be equivalent to an electro-driven mobility of ~9.5 x 10^{-8} m^2/V/s. The apparent mobility (μ_{app}) of apo-GroEL$_{14}$ and the electro-osmotic flow mobility (μ_{EOF}) were on average 8.1 x 10^{-8} m^2/V/s, and 1.1 x 10^{-7} m^2/V/s, respectively.

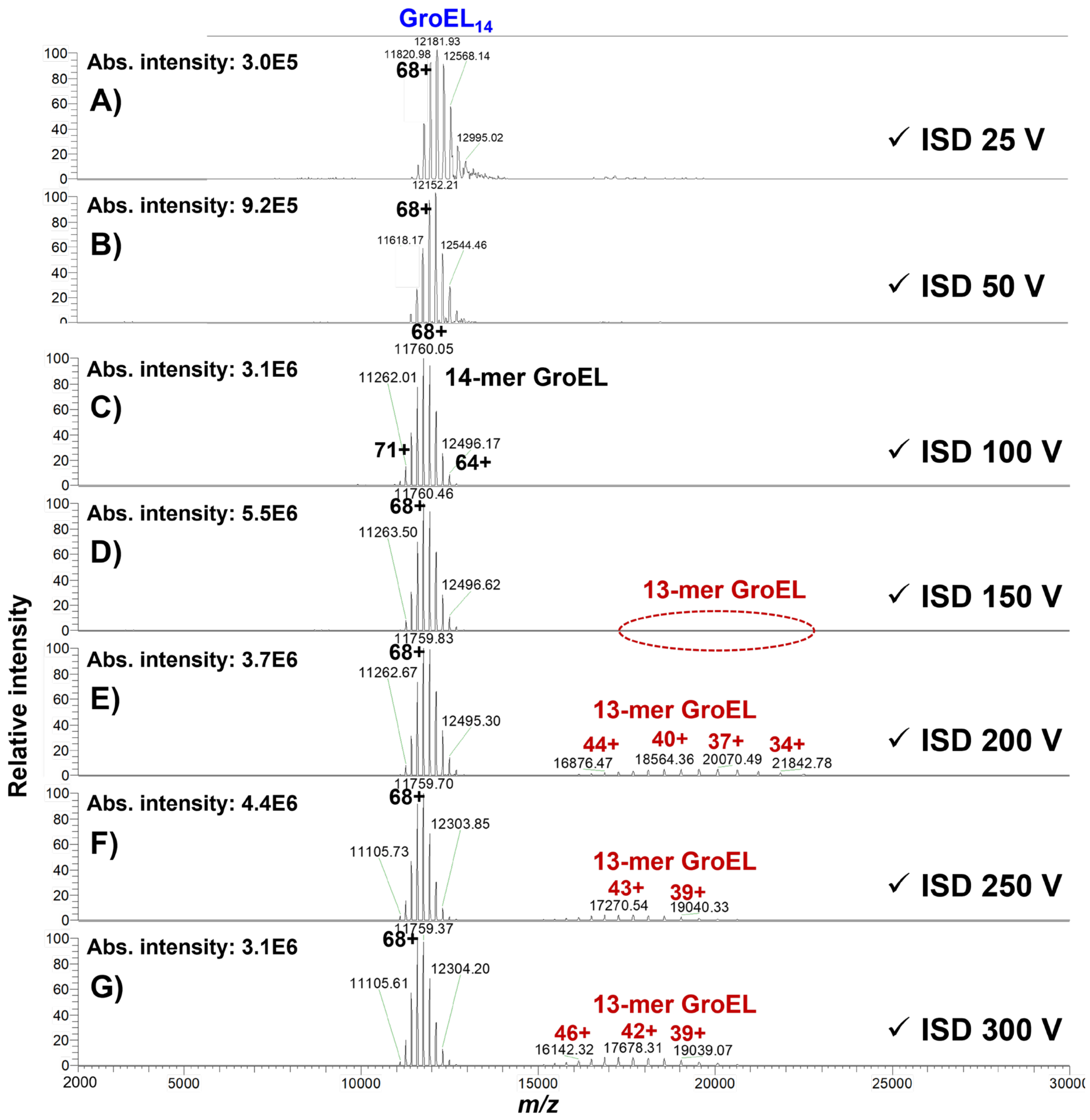
GroEL14
Abs. intensity: 3.0E5
A)
68+
12181.93
11820.98
12568.14
12995.02
✓ ISD 25 V
Abs. intensity: 9.2E5
B)
68+
12152.21
11618.17
12544.46
✓ ISD 50 V
Abs. intensity: 3.1E6
C)
68+
11760.05
11262.01
14-mer GroEL
71+
12496.17
64+
✓ ISD 100 V
Abs. intensity: 5.5E6
D)
68+
11760.46
11263.50
12496.62
13-mer GroEL
✓ ISD 150 V
Abs. intensity: 3.7E6
E)
68+
11759.83
11262.67
12495.30
13-mer GroEL
44+ 40+ 37+ 34+
16876.47 18564.36 20070.49 21842.78
✓ ISD 200 V
Abs. intensity: 4.4E6
F)
68+
11759.70
12303.85
11105.73
13-mer GroEL
43+ 39+
17270.54 19040.33
✓ ISD 250 V
Abs. intensity: 3.1E6
G)
68+
11759.37
12304.20
11105.61
13-mer GroEL
46+ 42+ 39+
16142.32 17678.31 19039.07
✓ ISD 300 V
Relative intensity
m/z

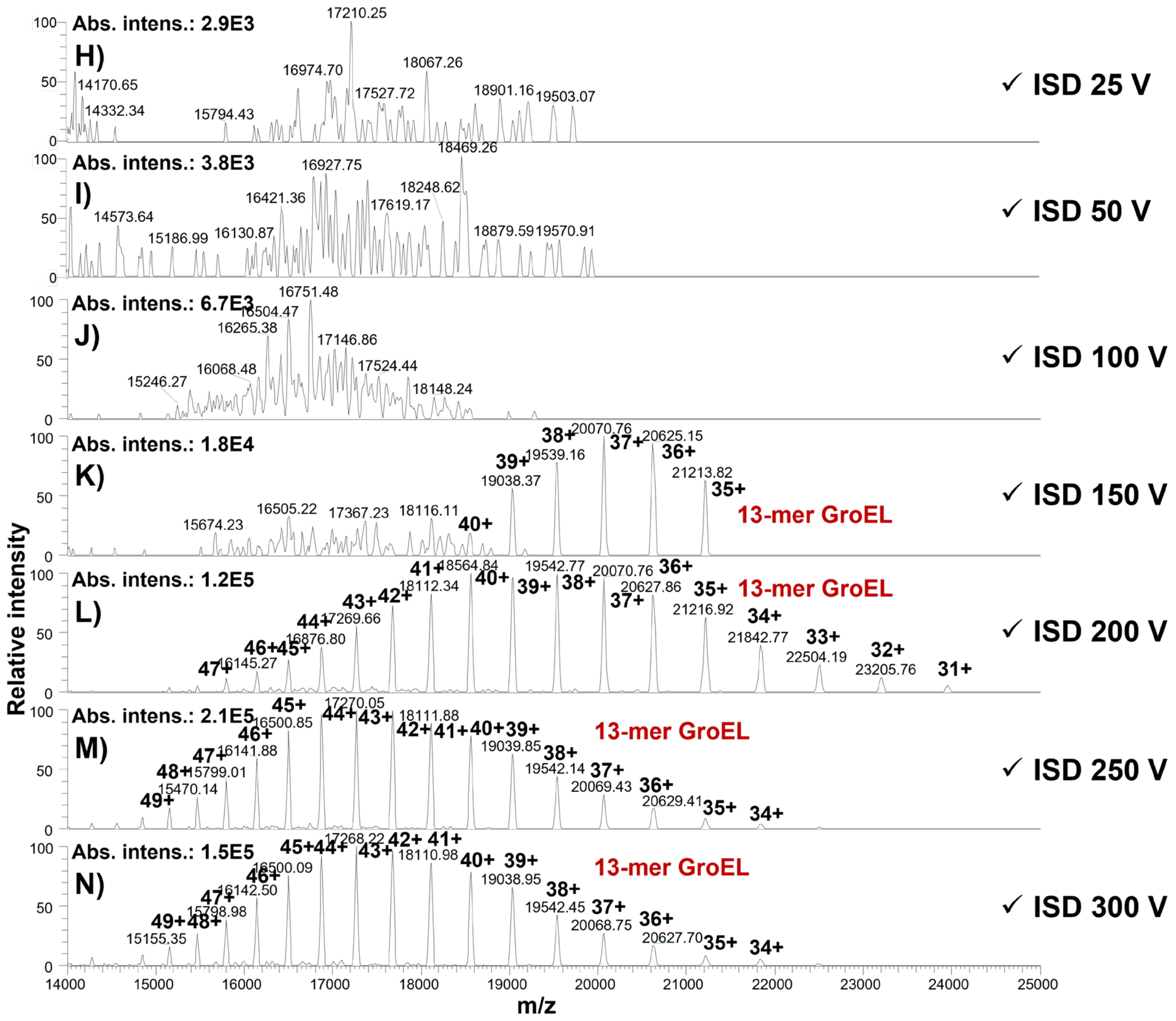

Figure S3: Native CE-MS analysis of mutant GroEL$_{14}$. A-G) Mass spectra acquired in the *m/z* 2,000-30,000 scan range varying the **in-source desolvation (ISD) voltage** from 25 to 300 V. Panels H-N are zoomed-in regions of panels A-G, respectively, in the *m/z* 14,000-25,000 scan range to show the detection of the GroEL 13-mer applying an ISD voltage ≥150 V.

O) **Mutant GroEL$_{14}$**

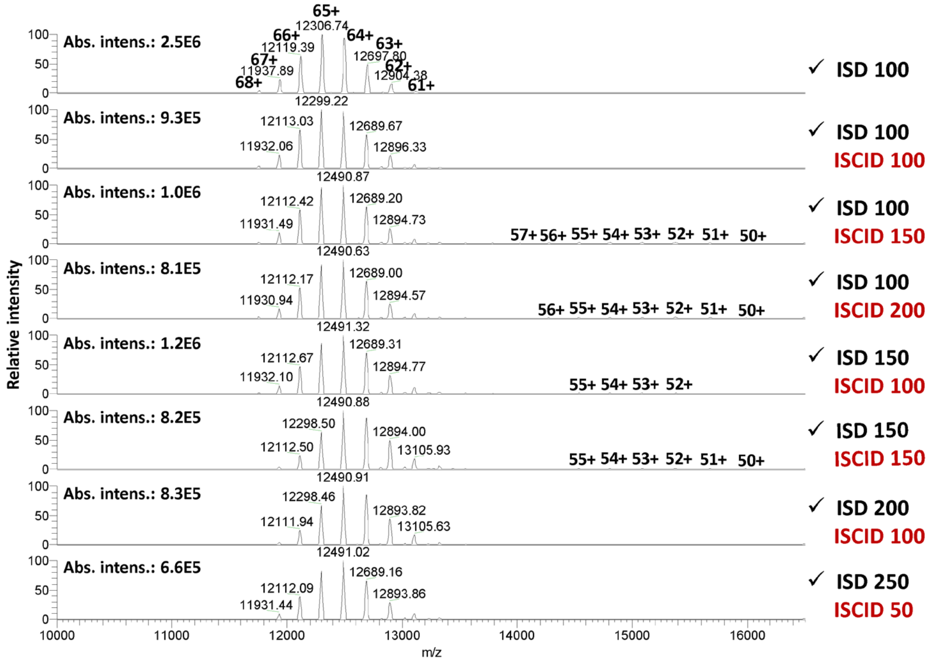

P)

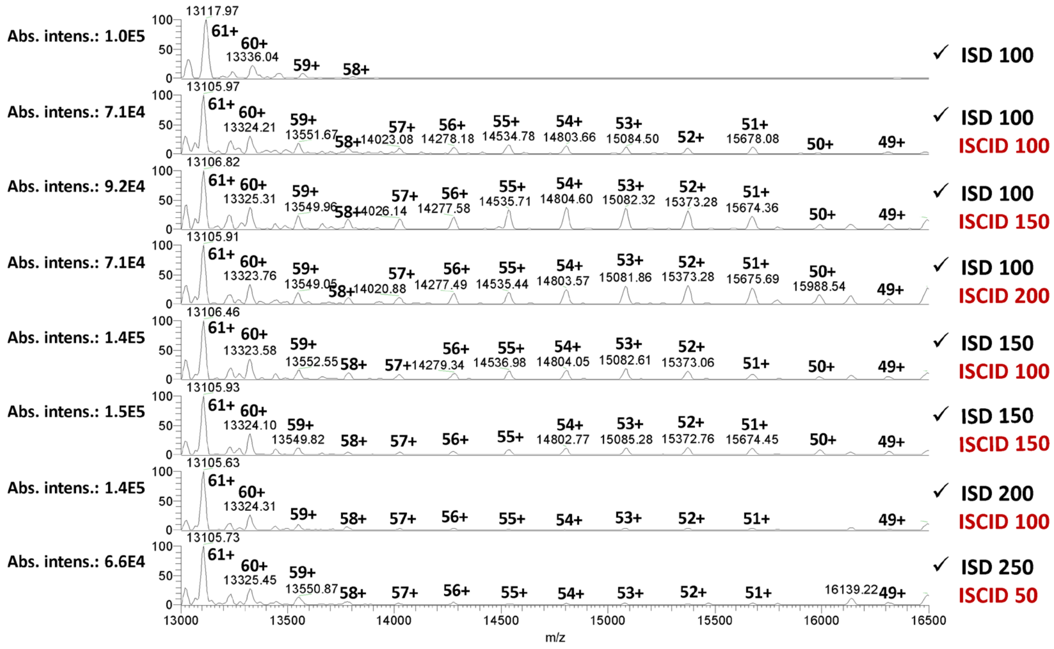

Figure S3: Native CE-MS analysis of 6 fmol mutant GroEL$_{14}$ varying ISD and in-source collision-induced dissociation (ISCID) energies. Two charge state distributions are detected in the *m/z* 10,000-16,500 scan range, corresponding to a less folded state (higher intensity charge state envelope centered on the 65+ ion) and a more folded state (lower intensity charge state envelope centered on the 53+ ion) of the mutant GroEL$_{14}$. Panel P is a zoomed-in region of panel O in the *m/z* 13,000-16,500 scan range.

Q) **WT GroEL$_{14}$**

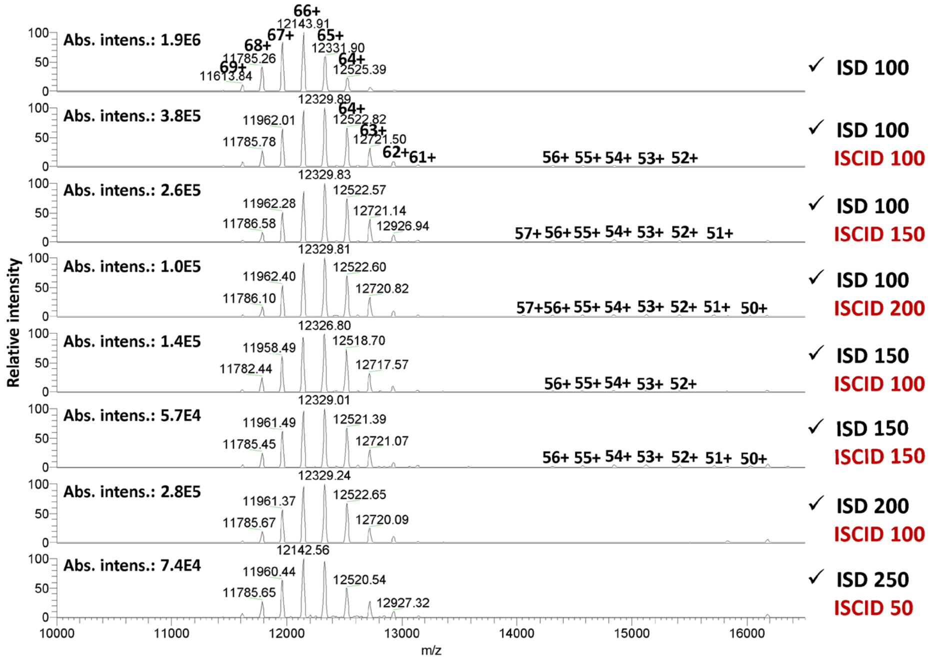

R)

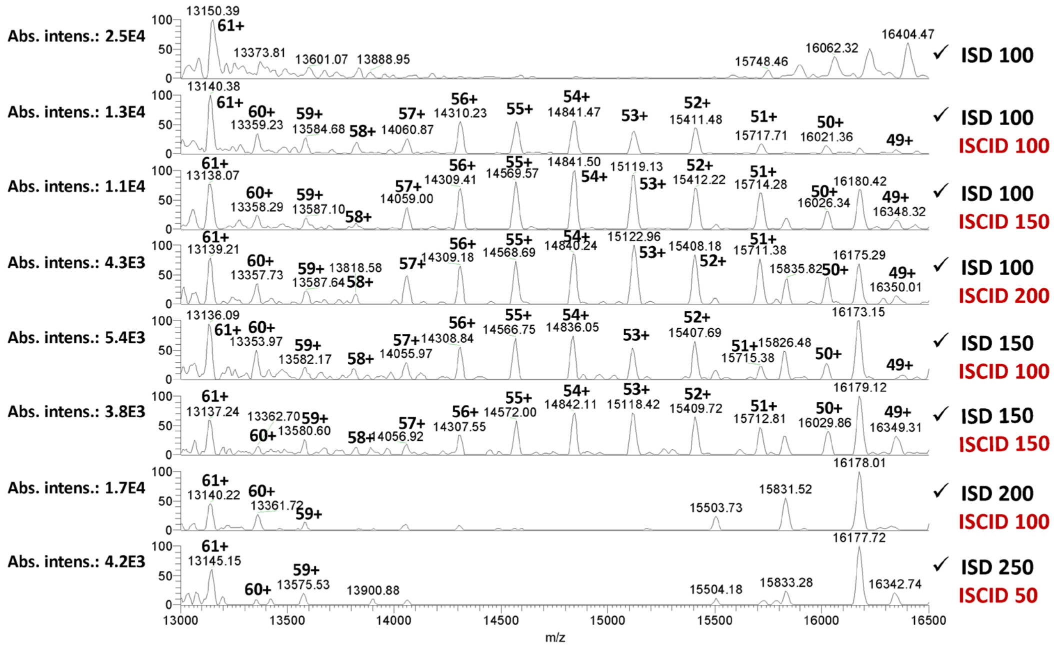

Figure S3: Native CE-MS analysis of 3 fmol WT GroEL$_{14}$ varying ISD and ISCID energies. Two charge state distributions are detected in the *m/z* 10,000-16,500 scan range, corresponding to a less folded state (higher intensity charge state envelope centered on the 66+ ion) and a more folded state (lower intensity charge state envelope centered on the 54+ ion) of the WT GroEL$_{14}$. Panel R is a zoomed-in region of panel Q in the *m/z* 13,000-16,500 scan range.

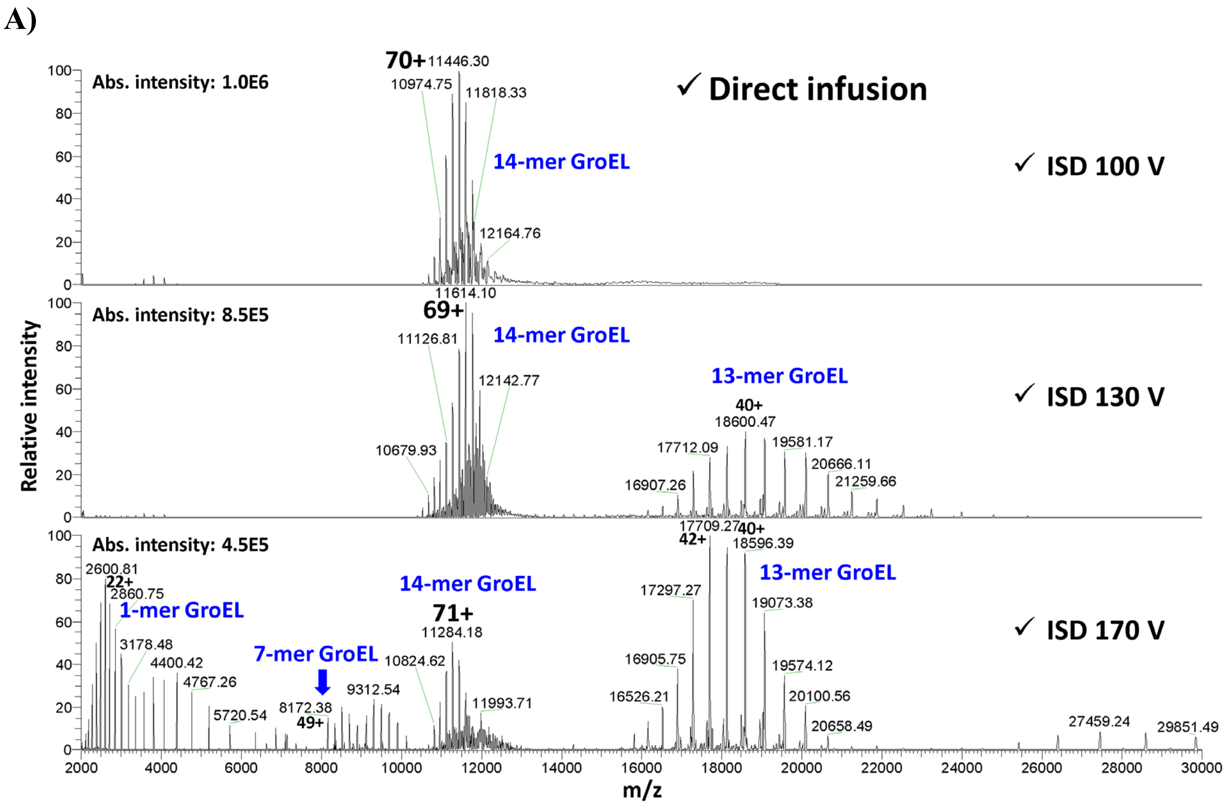

Figure S4: A) **Native ESI-MS analysis of DR GroEL$_{14}$ by direct infusion** of a mixture of 34 µM GroEL$_{14}$ in 200 mM ammonium acetate pH 6.7, varying the ISD voltage from 100 to 170 V.

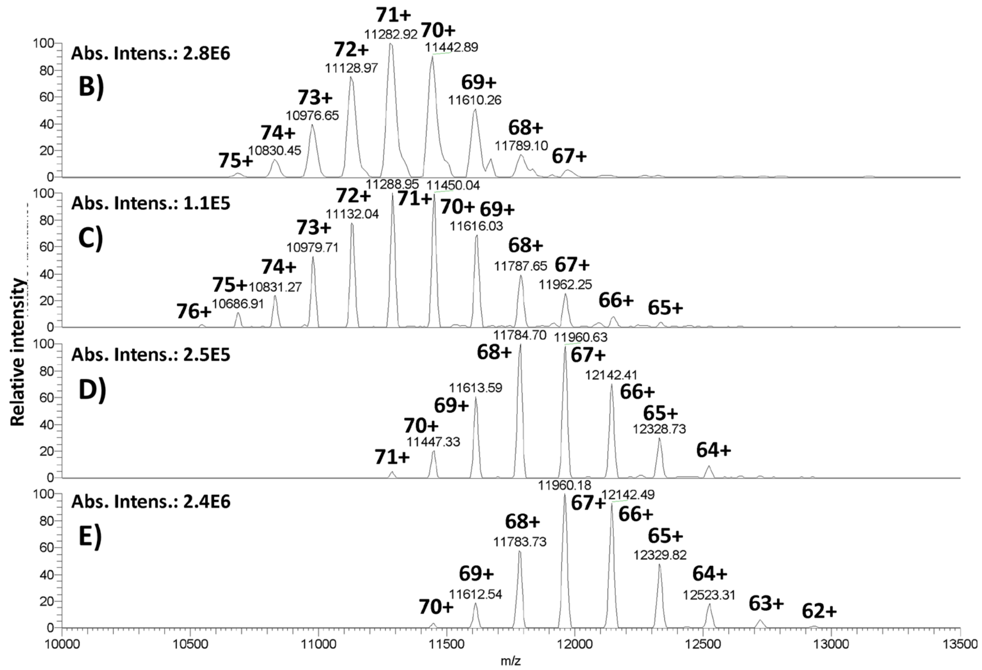

Figure S4: B-E) **Native ESI-MS and CE-ESI-MS analyses of GroEL$_{14}$.** B-D) Offline analysis of one GroEL$_{14}$ sample isolated with size exclusion chromatography (SEC) using B) **MS-based direct infusion** (infusion of 34 µM GroEL$_{14}$ in 200 mM ammonium acetate pH 6.7), C) **CE-MS-based direct infusion** (infusion of 6 µM GroEL$_{14}$ in 40 mM ammonium acetate pH 6.7), and D) conventional **CE-MS** (injection of 1 nL of 6 µM GroEL$_{14}$ in 40 mM ammonium acetate pH 6.7, 6 fmol injected). E) Online **CE-MS** analysis of GroEL$_{14}$ (injection of 1 nL of 6 µM GroEL$_{14}$ in refolding buffer pH 7.5, 6 fmol injected).

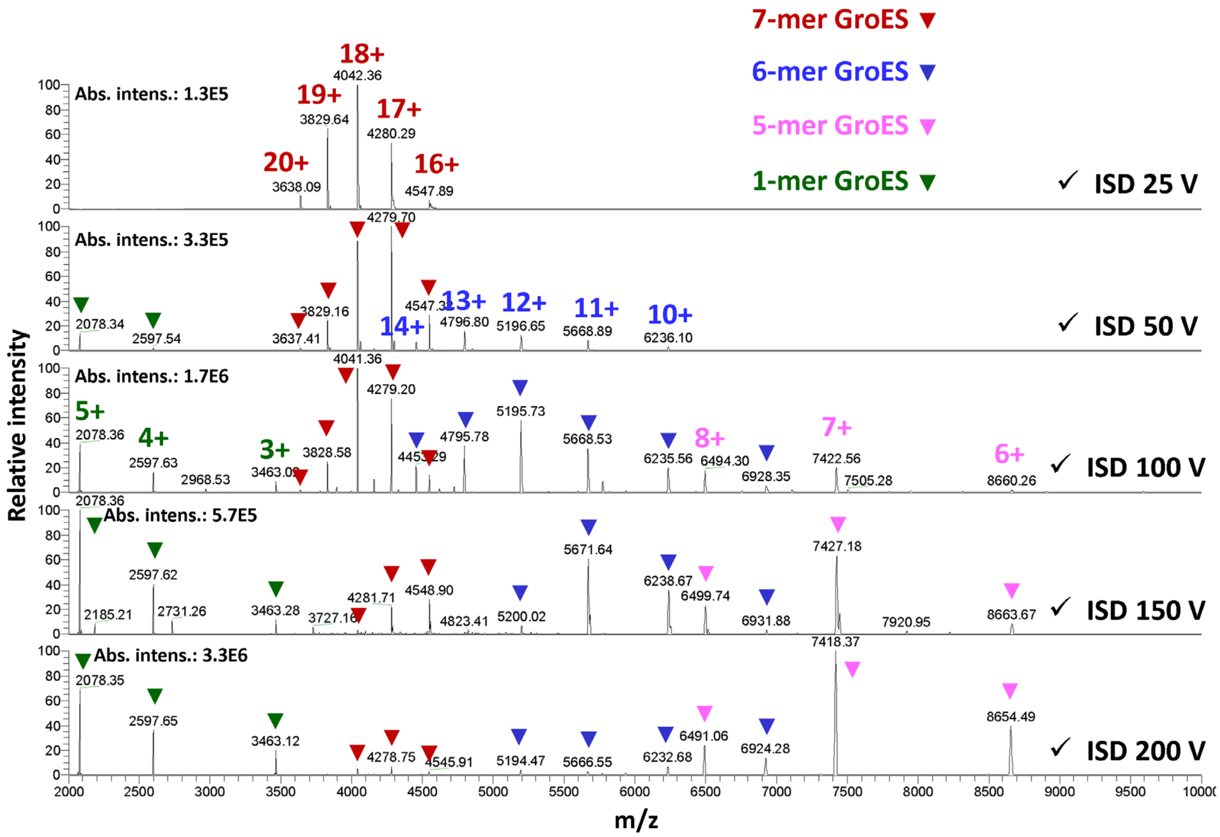

Figure S5: Native CE-MS analysis of GroES$_7$. Mass spectra acquired in the *m/z* 2,000-10,000 scan range varying the ISD voltage from 25 to 200 V. Applying an ISD voltage of 100 V, GroES$_7$ is partially dissociated into GroES$_6$ and GroES$_5$ complexes. Applying an ISD voltage of 150 V, the GroES$_6$ and GroES$_5$ complexes are detected at very high abundances. Finally, an ISD voltage of 200 V induces the almost complete dissociation of GroES$_7$ into GroES$_5$.

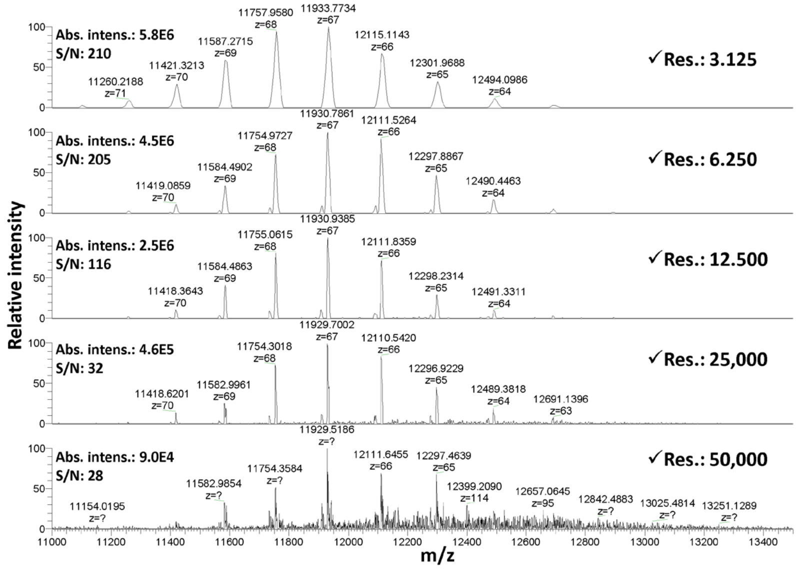

MS Resolution	$GroEL_{14}$ Mutant Mr_{exp} (Da)	Δ ppm *vs* Mr_{th}*	Δ ppm *vs* Mr_{th}**
3,125	799,440.25	516	321
6,250	799,303.63	345	150
12,500	799,287.06	324	129
25,000	799,258.44	289	93
50,000	799.182.75	194	-1

Mr_{th}*	Mr_{th}**
N-term Met truncated	N-term Met truncated
No adducts	6 Na^+ and 1 NH_4^+ adduct ions
799,027.77 Da	799,183.74 Da

Figure S6: Native CE-MS analysis of mutant $GroEL_{14}$. Mass spectra acquired varying the MS resolution from 3,125 to 50,000 at 200 *m/z*. The tables on the right-hand side show the experimental molecular masses (Mr_{exp}) obtained after the deconvolution of the mass spectra acquired at the selected MS resolutions, and the theoretical molecular mass (Mr_{th}) of the DR mutant $GroEL_{14}$ with fourteen N-terminal methionine cleaved monomers. For MS resolution ≥6,250, an additional charge state envelope was detected at lower *m/z* compared to apo-$GroEL_{14}$, which presumably corresponds to a C-terminal cysteine truncated form of $GroEL_{14}$ (Mr_{exp} 797,873.06 Da, ΔM 362 ppm).

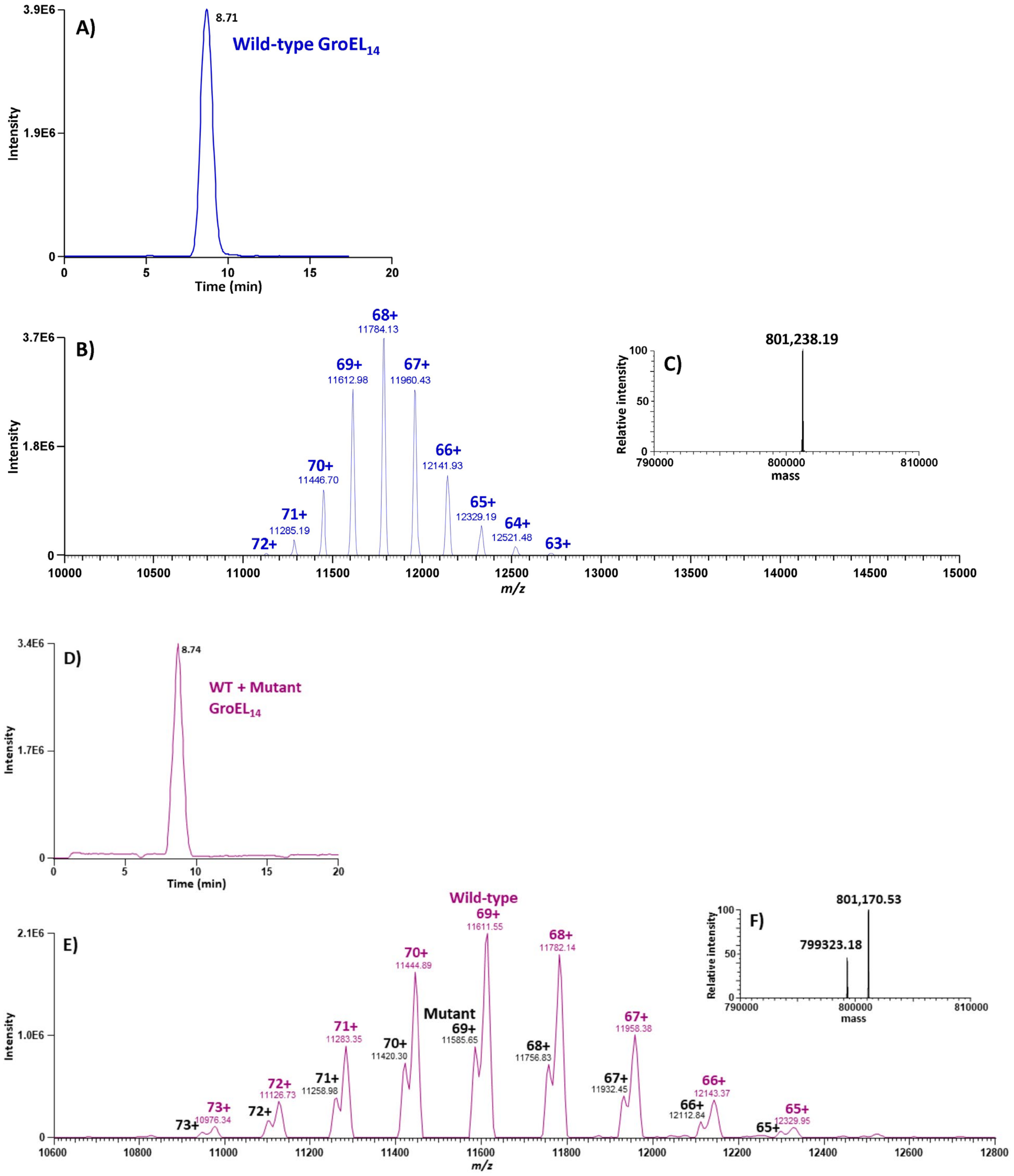

Figure S7: Native CE-MS analyses of WT and mutant $GroEL_{14}$. A-D) BPEs recorded in the CE-MS analyses of (A) WT $GroEL_{14}$ (6 fmol injected), and (D) a mixture of equimolar quantities of WT and mutant $GroEL_{14}$. (6 fmol of each injected). B-E) Mass spectra integrated across the BPE peaks shown in panels A and D, respectively. C-F) Molecular masses obtained from the deconvolution of the mass spectra shown in panels B and E, respectively.

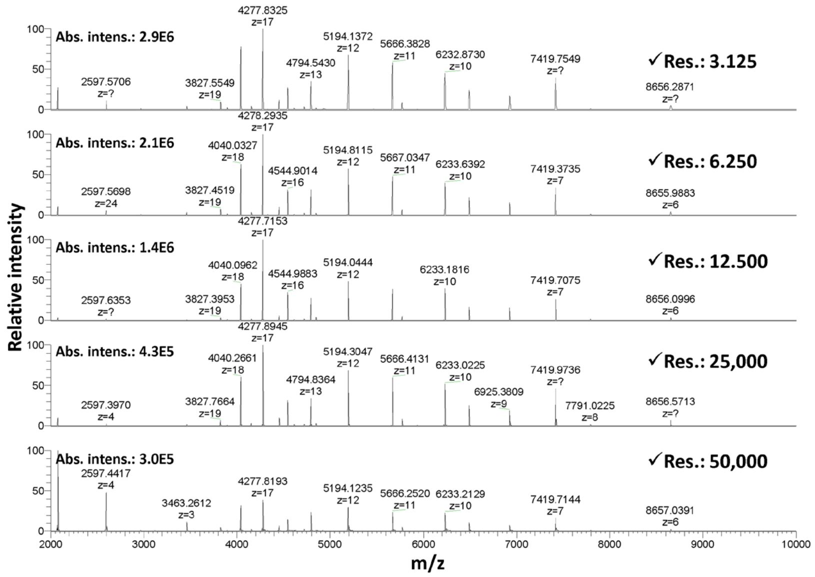

MS Resolution	$GroES_7$ Mr_{exp} (Da)	Δ ppm *vs* Mr_{th}
3,125	72,711.48	39
6,250	72,710.13	20
12,500	72,706.00	-36
25,000	72,705.09	-49
50,000	72,705.53	-43

Mr_{th}
No N-term Met truncated
72,708.65 Da

Figure S8: Native CE-MS analysis of $GroES_7$. Mass spectra acquired varying the MS resolution from 3,125 to 50,000 at 200 *m/z*. The tables on the right-hand side show the Mr_{exp} obtained after the deconvolution of the mass spectra acquired at the selected MS resolutions, and the Mr_{th} of the WT $GroES_7$ with seven non-truncated monomers (i.e., absence of N-terminal methionine cleavage).

Supplementary Note 1

We conducted native MS analysis of SEC isolates of GroEL complexes using (a) direct infusion from pulled glass nanoESI emitter tips into the mass spectrometer as well as native MS analyses of the same SEC GroEL isolates using two different modes: (b) direct infusion through the CE capillary and the CESI sheathless interface and (c) by conducting conventional CE-MS on the same CESI platform (**Figure S4BD**). The analysis of the SEC GroEL isolates with nanoESI-MS- and CESI-MS-based direct infusion resulted in a noticeably lower spectral quality (i.e., the signal was noisier, and broader peaks were observed in acquired spectra, perhaps due to adduct formation) compared to the data acquired using our developed online CE-MS method (**Figure S4BCE**). In addition, the infusion experiments required the injection of the GroEL complexes at a higher concentration (34 µM vs. 6 µM), compared to our developed technique. The analysis of the SEC isolates using conventional CE-MS resulted in a similar spectral quality, compared to the analyses acquired using our developed CE-MS method, but the signal intensity level was significantly decreased (on average the signal intensity was 10-fold lower in offline CE-MS analysis of SEC GroEL isolates compared to online CE-MS analysis of GroEL complexes) (**Figure S4DE**).

In all direct infusion experiments (nanoESI-MS- or CESI-MS-based) conducted using SEC GroEL isolates, we also noticed that the detected charge state distributions corresponded to a less folded state, i.e. more denatured state of the GroEL complexes, compared to our developed CE-MS method, which resulted in the detection of a more folded/more native state of the GroEL complexes (e.g., the charge state envelopes of apo-$GroEL_{14}$ were centered on average around the 71+ and 67+ ions, respectively, **Figure S4BCE**). Interestingly, a better preservation of the folded/native state of the GroEL complexes was also observed in online pseudo-MS^2 analysis of GroEL. The detected charge state envelopes of the ejected GroEL monomers (produced by in-source fragmentation of the GroEL 14-mer) were centered around the 27+ and 15+ ions, for direct infusion experiments and our CE-MS method, respectively, and therefore corresponded to a less and more folded GroEL monomer, respectively.

Finally, nanoESI-MS- and CESI-MS-based direct infusion of SEC isolates required substantially larger amounts of GroEL complexes for sample preparation and direct infusion analyses, in comparison to online CE-MS analysis of GroEL complexes. Indeed, at least 20 µL of GroEL complexes at a concentration of 34 µM were required for SEC fractionation, and the infusion of at least 1,000 nL of one SEC isolate was necessary to detect the GroEL complexes at a signal intensity level similar to the signal level recorded using our optimized CE-MS method. In contrast, only 3 µL of GroEL complexes at a concentration of 6 µM were sufficient in our CE-MS experimental setup, where the injected plugs were as small as 1 nL.

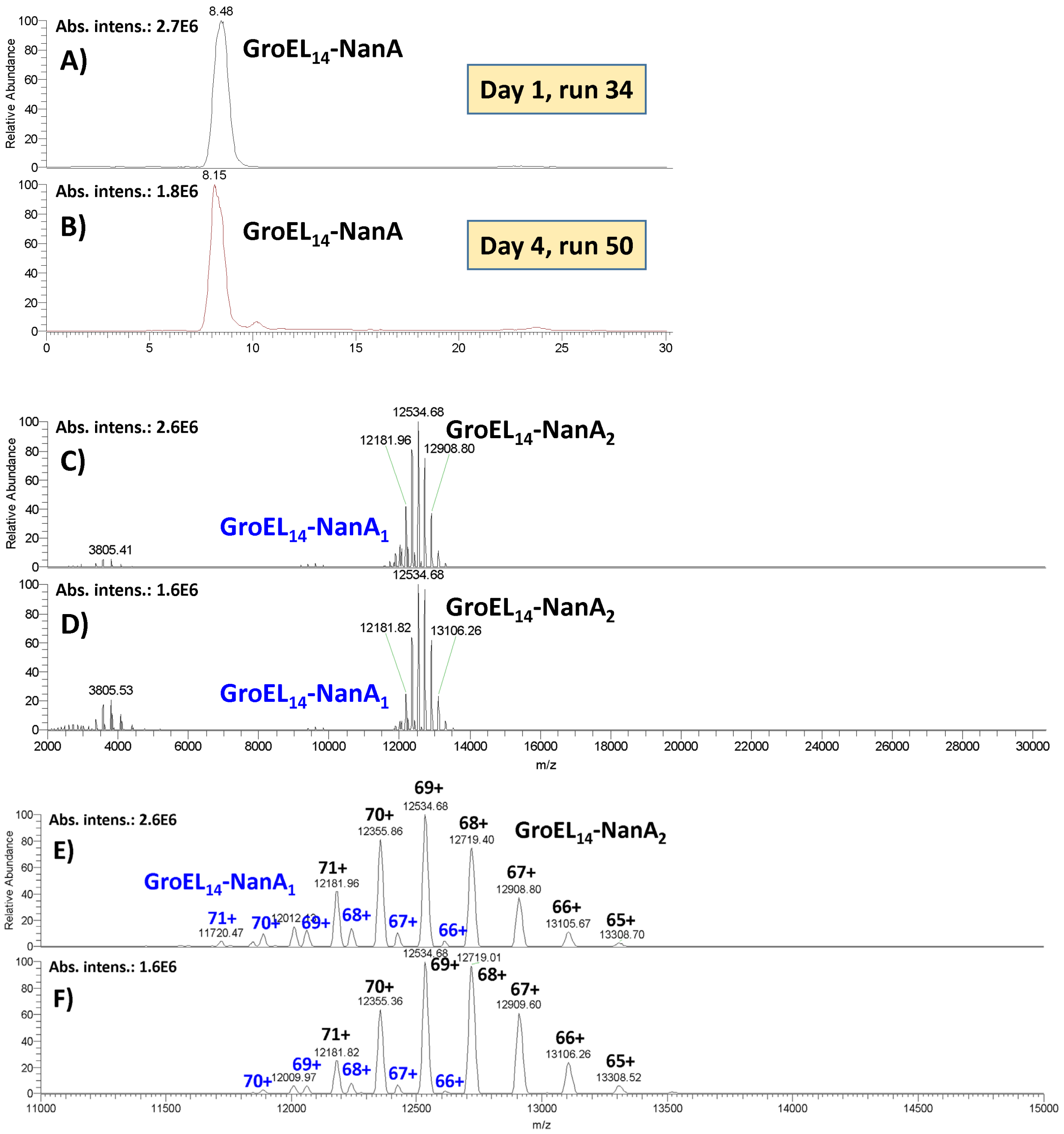

Figure S9: Stability study of the GroEL$_{14}$-NanA complex. A-B) BPEs recorded in the CE-MS analysis of GroEL$_{14}$-NanA complex. A) CE-MS analysis of the complex ~30 min after the mixing of 6 µM of GroEL$_{14}$ and 6 µM of NanA$_1$ monomer (the analysis shown in panel A corresponds to the 34th run performed on the same BFS capillary). B) CE-MS analysis of the same GroEL$_{14}$-NanA complex after its storage at 4°C for four days (the analysis shown in panel B corresponds to the 50th run performed on the same BFS capillary). C-D) Mass spectra integrated across the BPE peaks shown in panels A and B, respectively. Panels E and F are zoomed-in regions of panels C and D, respectively, in the *m/z* 11,000-15,000 scan range.

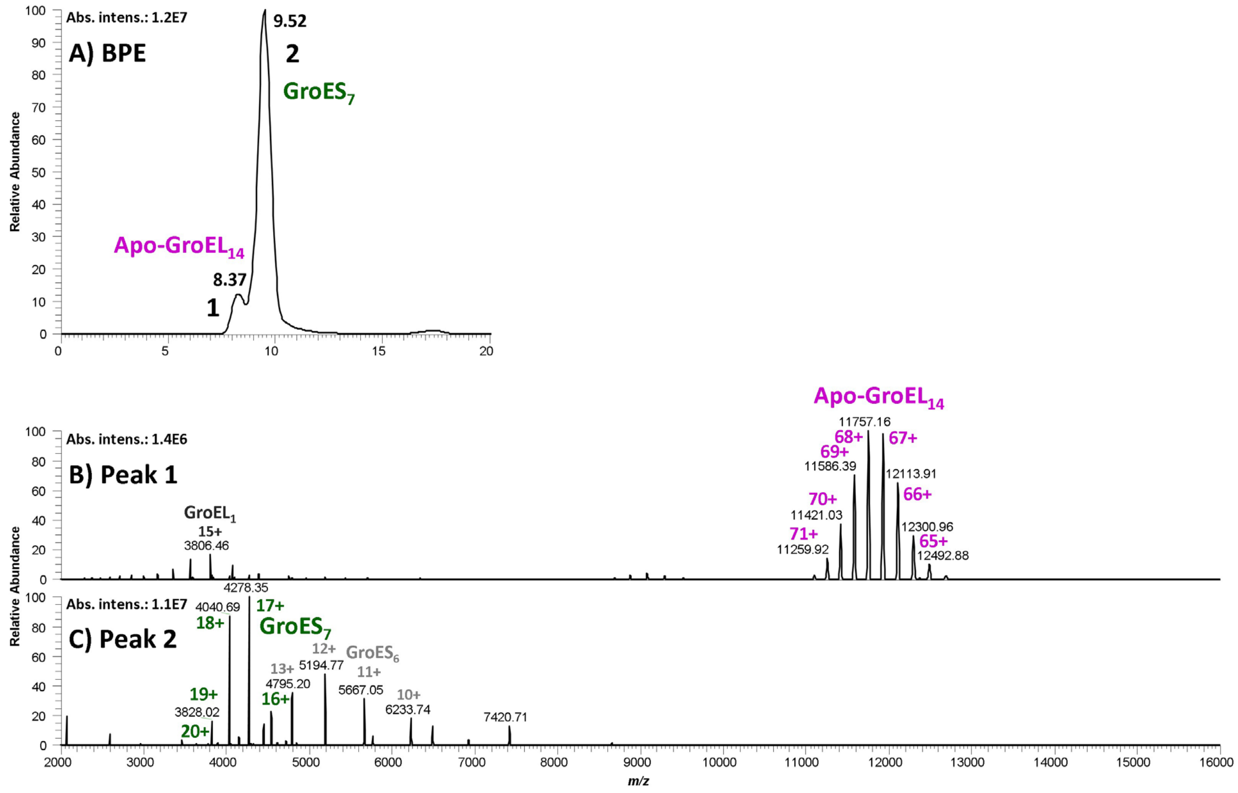

Figure S10: A) Example of **CE-MS analysis of a mixture of GroEL$_{14}$** (6 µM), **GroES$_7$** (6 µM), **and ATPγS** (1 mM) in a capillary filled with 50 mM ammonium acetate BGE devoid of ATPγS. A) BPE trace. B-C) Mass spectra integrated across the two BPE peaks shown in panel A.

D)

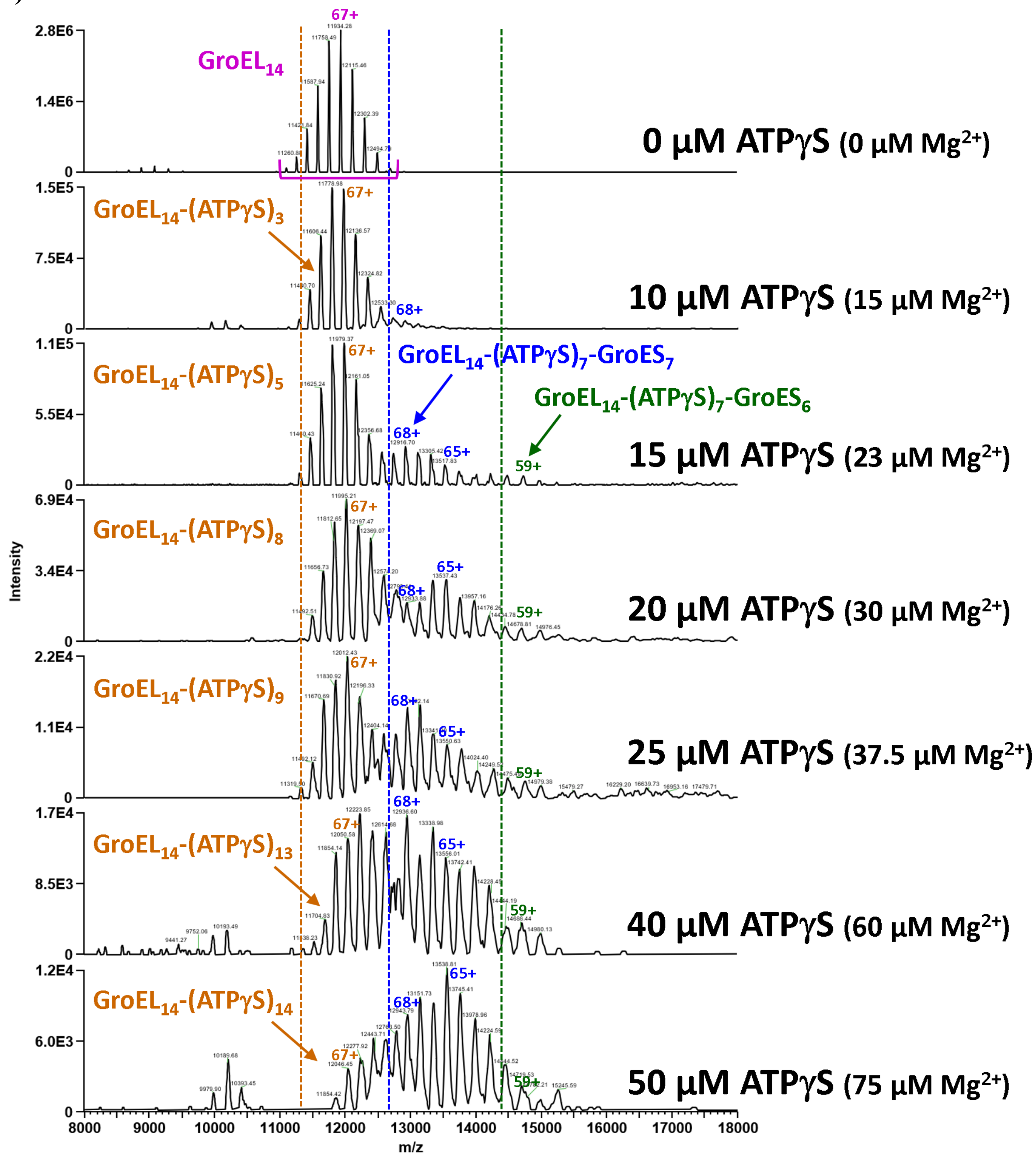

Figure S10: D) **CE-MS analysis of a mixture of GroEL$_{14}$** (6 µM) **and GroES$_7$** (6 µM) in a capillary filled with **50 mM ammonium acetate BGEs containing various concentrations of ATPγS** (from 0 to 50 µM). Each increment of ATPγS added to the BGE is associated with an increased concentration of Mg^{2+} in the respective BGE, as indicated in brackets, since the ATPγS stock solution used to prepare the different BGEs was diluted in a refolding buffer containing 15 mM $MgCl_2$ (see Experimental Section).

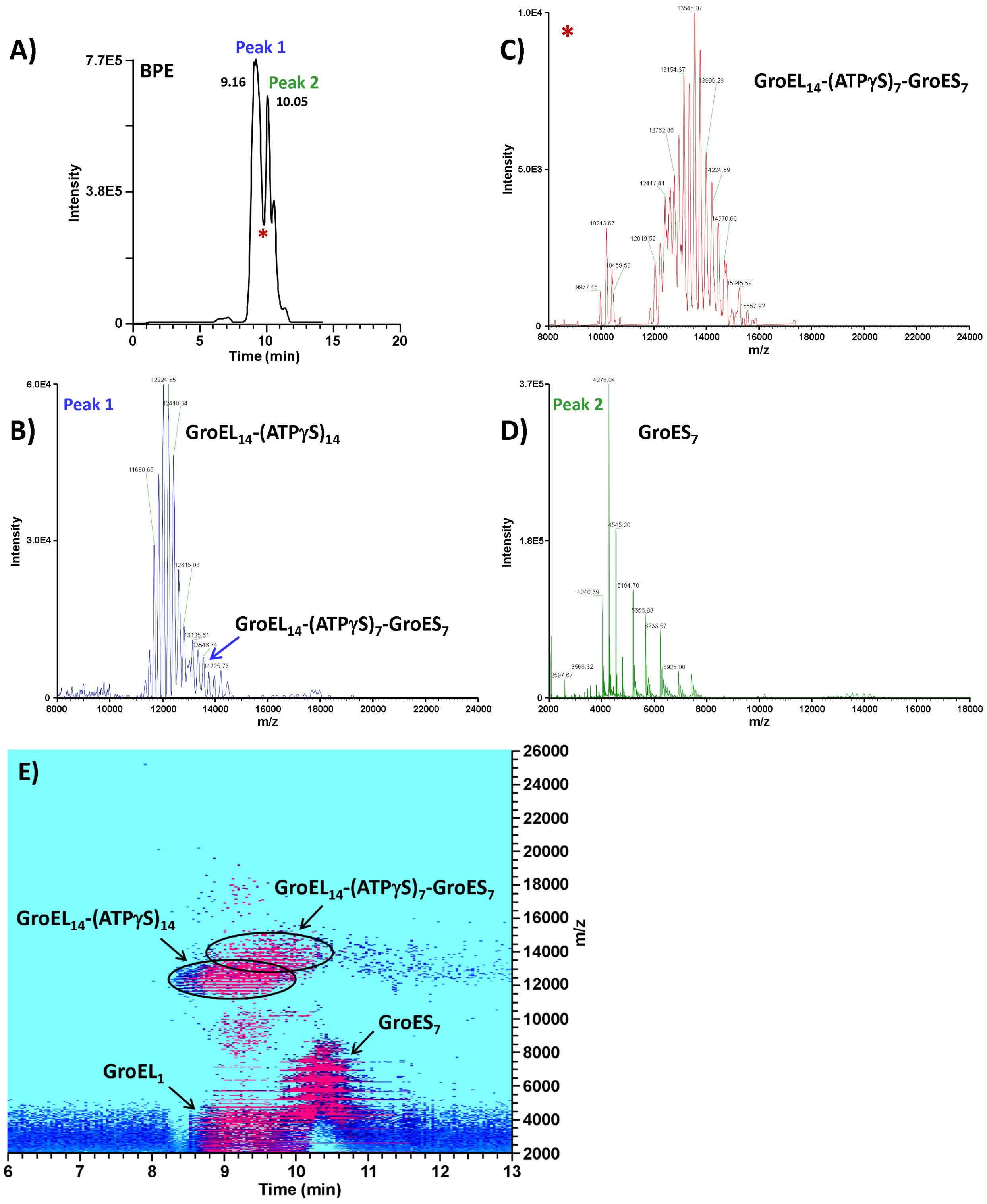

Figure S11: Example of characteristic **CE-MS analysis of the GroEL$_{14}$-(ATPγS)$_7$-GroES$_7$ complex** carried out with the injection of a mixture of GroEL$_{14}$ (6 µM) and GroES$_7$ (6 µM) in a capillary filled with 50 mM ammonium acetate BGE containing 50 µM of ATPγS. A) BPE profile showing two major separated peaks. B-D) Mass spectra integrated across (B) the first BPE peak

(from 9.04 to 9.26 min), (C) the region between the two BPE peaks (from 9.71 to 10.00 min), and (D) the second BPE peak (from 10.11 to 10.19 min). E) Ion density map acquired in the CE-MS analysis of the $GroEL_{14}$-$(ATP\gamma S)_7$-$GroES_7$ complex. Two different charged state ion distributions are separated over time, corresponding to $GroEL_{14}$-$(ATP\gamma S)_{14}$ and $GroEL_{14}$-$(ATP\gamma S)_7$-$GroES_7$ complexes, respectively.

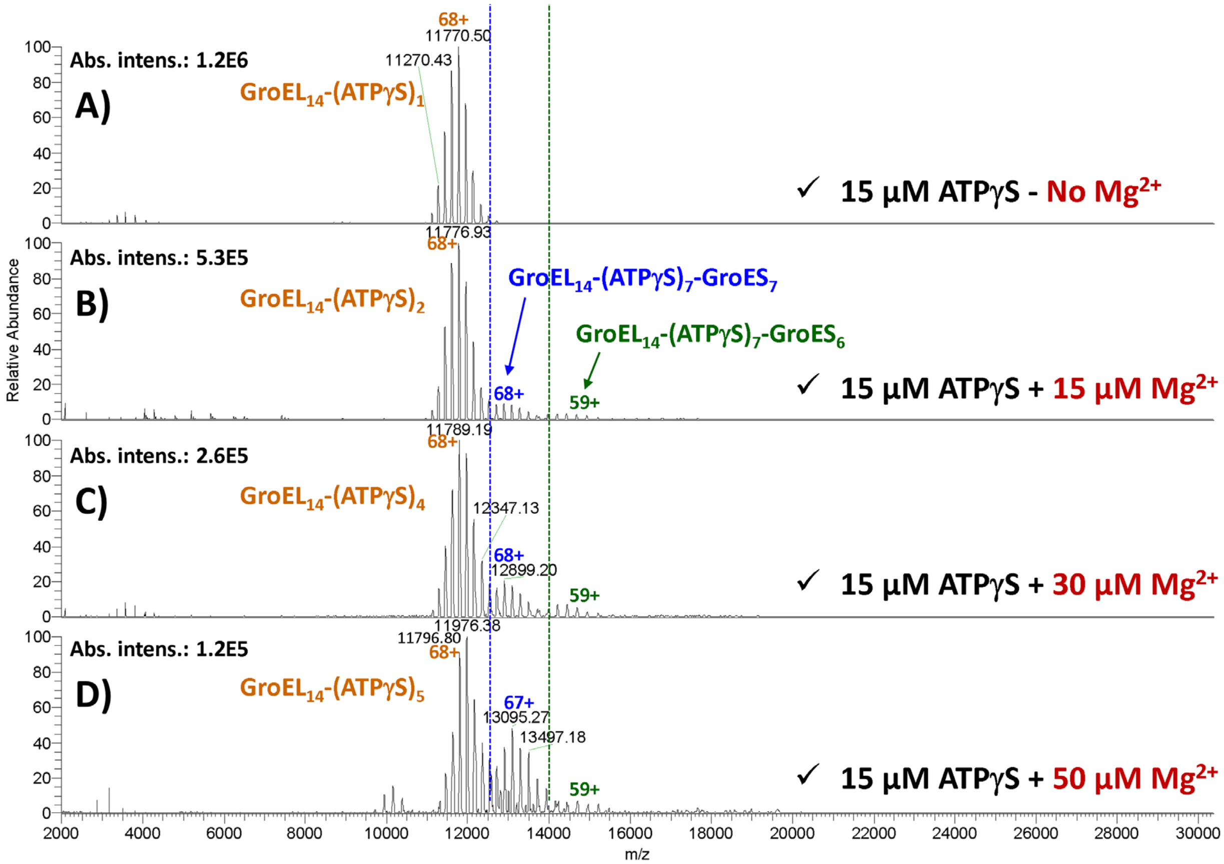

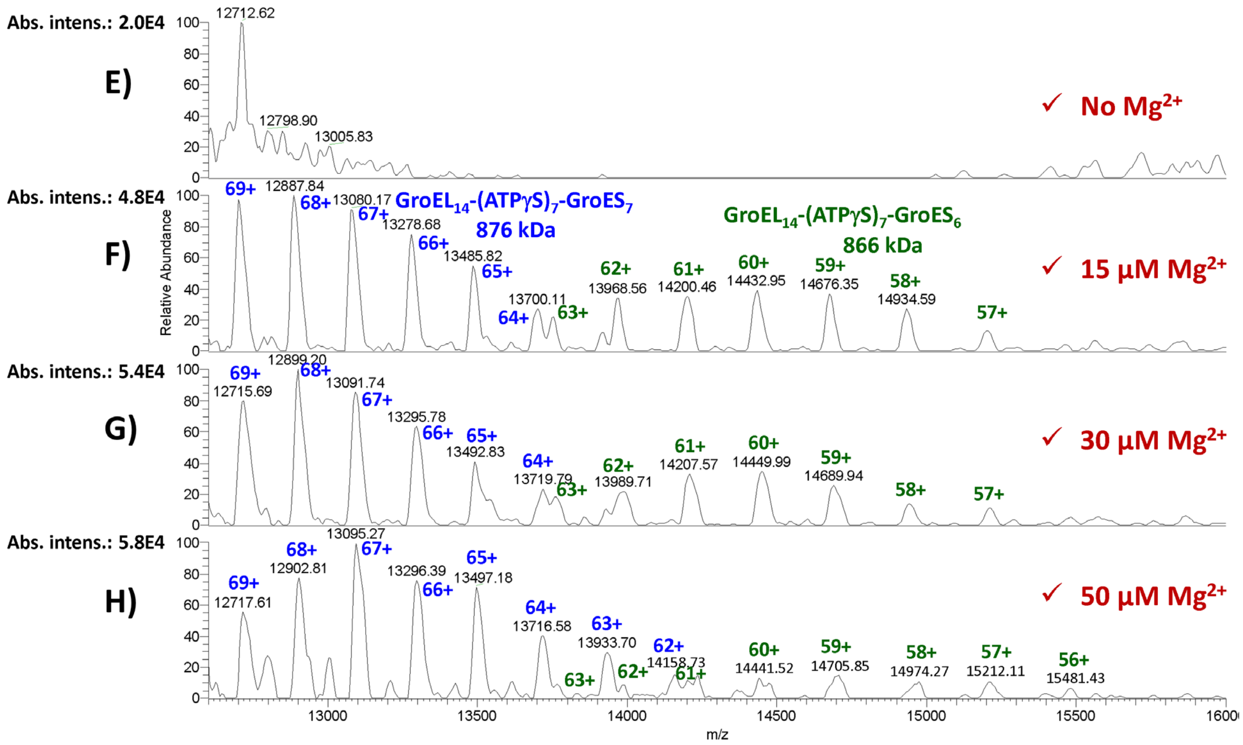

Figure S12: Role of Mg^{2+} ions in the stabilization of the $GroEL_{14}$-$(ATP\gamma S)_7$-$GroES_7$ complex. Example of characteristic CE-MS analysis carried out with the injection of a mixture of $GroEL_{14}$ (6 µM) and $GroES_7$ (6 µM) in a capillary filled with 50 mM ammonium acetate BGE containing 15 µM of ATPγS and various concentrations of Mg^{2+} (from 0 to 50 µM). Panels E-H are zoomed-in regions of panels A-D, respectively, in the *m/z* 12,600-16,000 scan range.

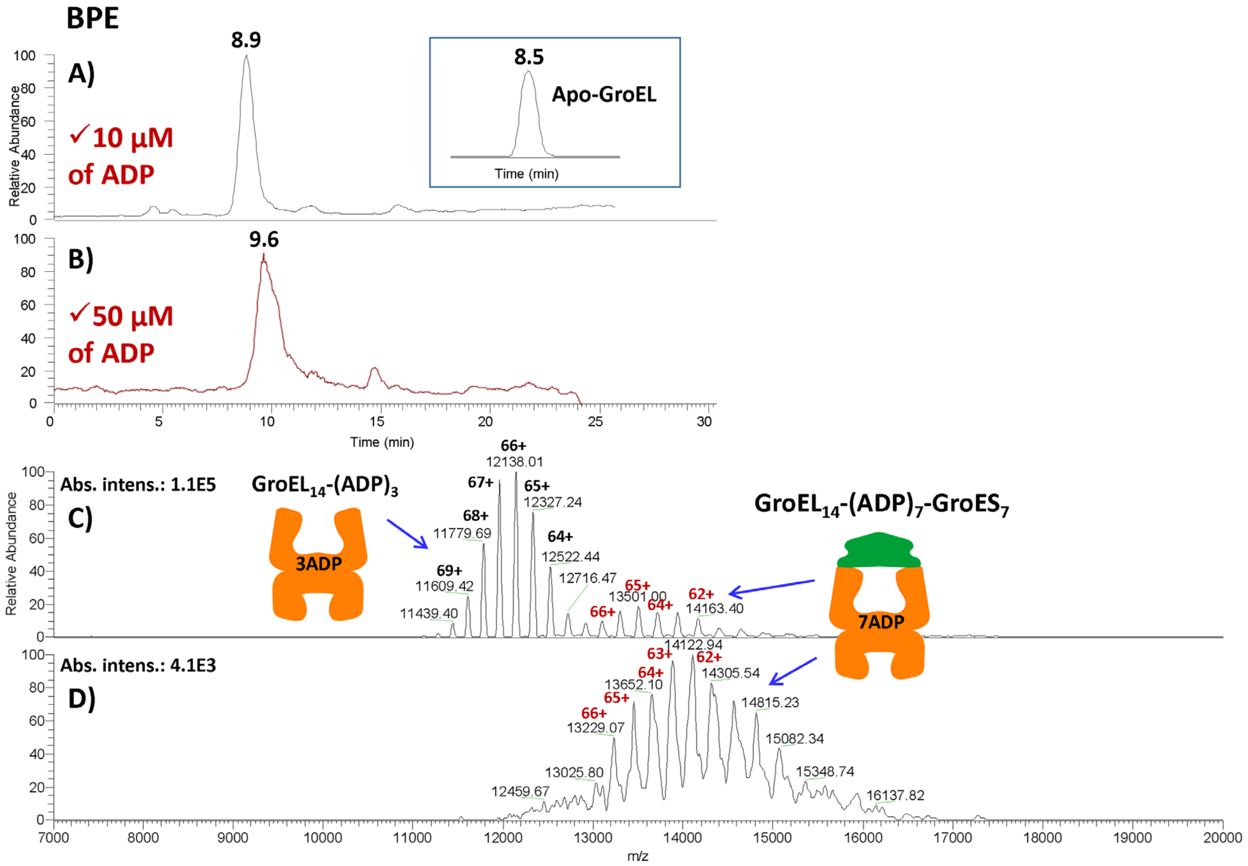

Figure S13: Example of characteristic **CE-MS analysis of the GroEL$_{14}$-(ADP)$_7$-GroES$_7$ complex.** A-B) BPEs recorded in the CE-MS analysis of a mixture of GroEL$_{14}$ (6 µM), GroES$_7$ (6 µM), and ADP (1 mM) in a capillary filled with 50 mM ammonium acetate BGE containing (A) 10 µM of ADP and (B) 50 µM of ADP. C-D) Mass spectra integrated across the BPE peaks shown in panels A and B, respectively.

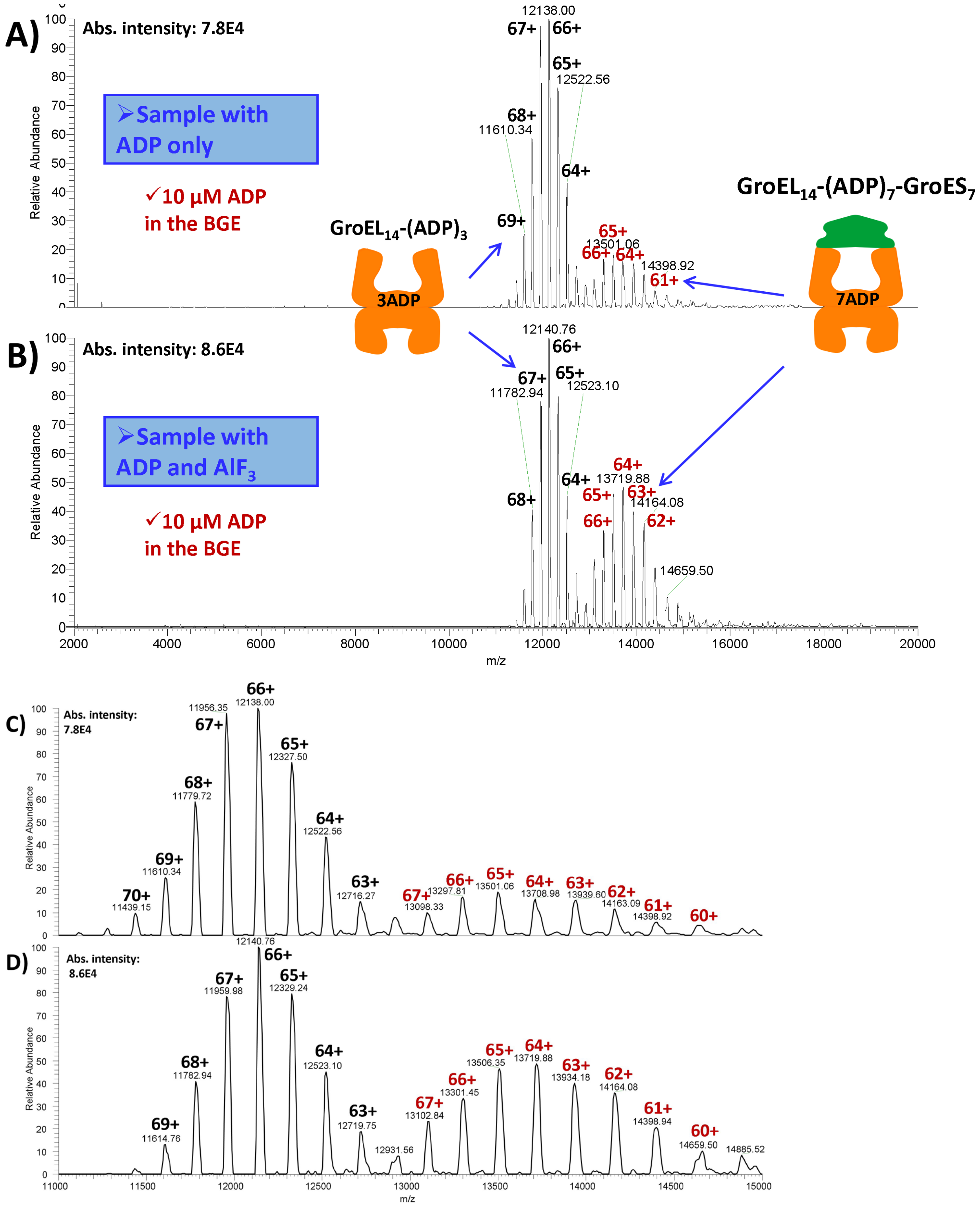

Figure S14: Example of characteristic **mass spectra acquired in the CE-MS analysis of a mixture of GroEL$_{14}$** (6 µM), **GroES$_7$** (6 µM), and **ADP** (1 mM) without (A-C) or with (B-D) **AlF$_3$** (1 mM) in a capillary filled with 50 mM ammonium acetate BGE containing 10 µM of ADP. Panels C and D are zoomed-in regions of panels A and B, respectively, in the *m/z* 11,000-15,000 scan range.

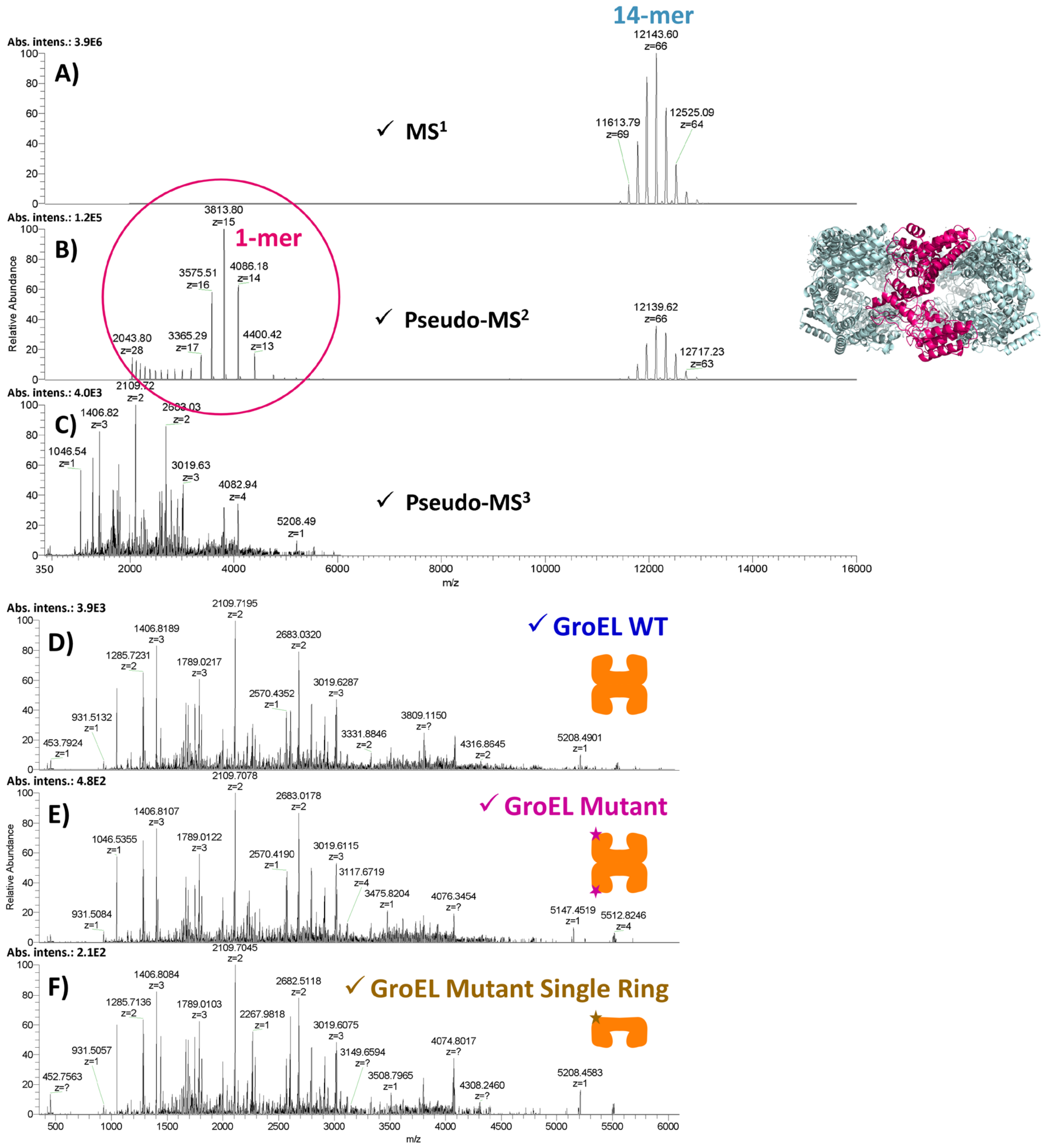

Figure S15: Native CE-pseudo-MS^3 analyses of WT and mutant DR GroEL$_{14}$, and mutant SR GroEL$_7$. A-C) Example of characteristic mass spectra acquired in (A) CE-MS^1 analysis of WT GroEL$_{14}$, B) CE-pseudo-MS^2 analysis of WT GroEL$_{14}$ to dissociate the tetradecameric GroEL$_{14}$ complex using in-source fragmentation applying higher ISD and ISCID voltages (See Experimental Section) in order to isolate and select the GroEL$_1$ monomeric subunits for their subsequent HCD fragmentation, and C) CE-pseudo-MS^3 analysis of WT GroEL$_{14}$ applying HCD collisional energy on the isolated and selected GroEL$_1$ monomeric subunits generated in CE-pseudo-MS^2. D-F) Example of characteristic mass spectra acquired in native CE-pseudo-MS^3 analyses of (D) WT GroEL$_{14}$, (E) DR mutant GroEL$_{14}$, and (F) SR mutant GroEL$_7$, which are illustrative of the high-quality of the HCD-based pseudo-MS^3 mass spectra generated for each GroEL species.

A)

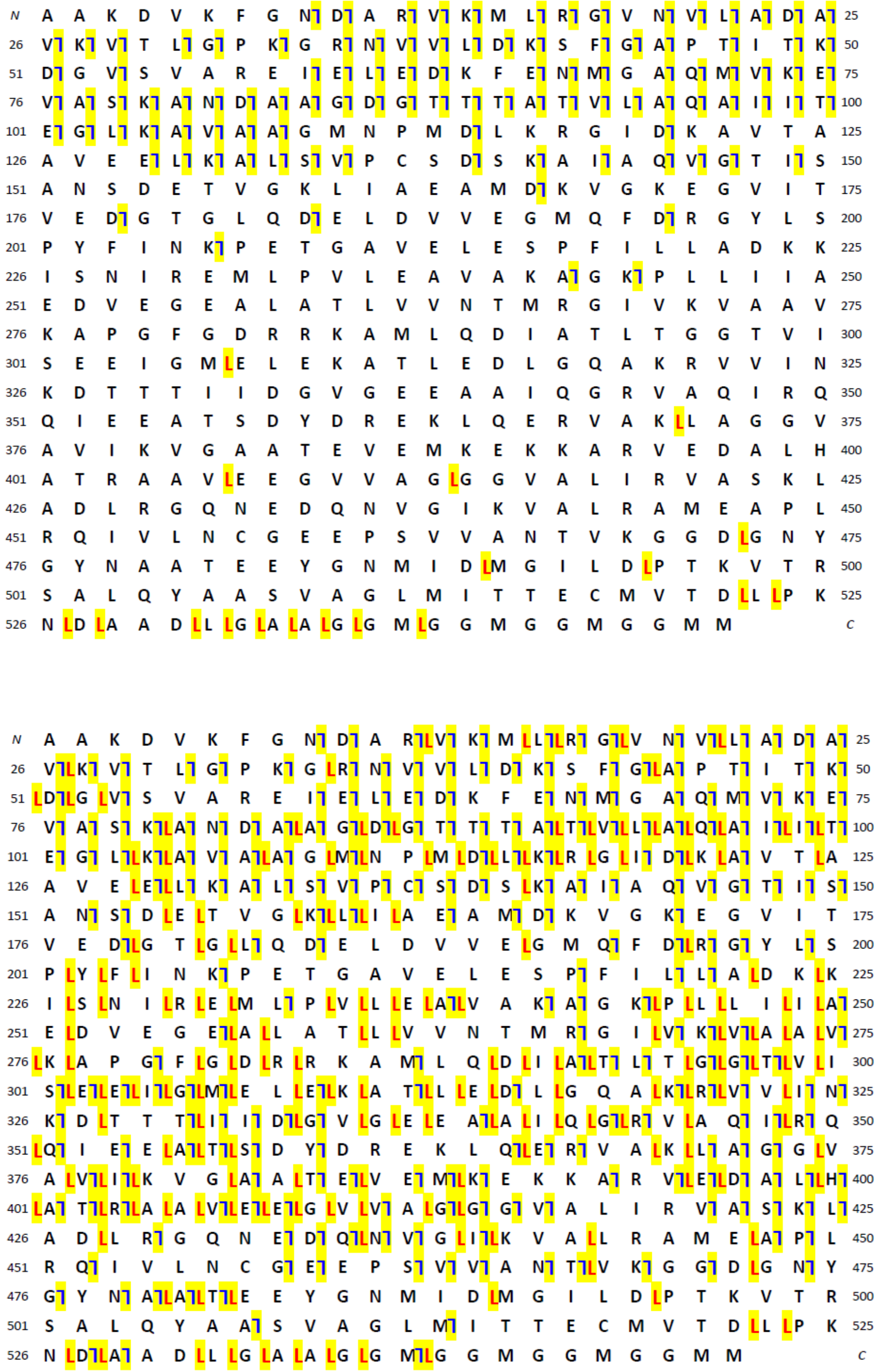

B)

Figure S16: Native CE-pseudo-MS^3 analysis of WT GroEL$_{14}$. Fragmentation maps of (A) *b* and *y* terminal fragment ions, and B) *b* and *y* terminal and *by* internal fragment ions generated in the CE-pseudo-MS^3 analysis of WT GroEL$_{14}$. The inclusion of internal fragments increased the sequence coverage from 23% to 63%.

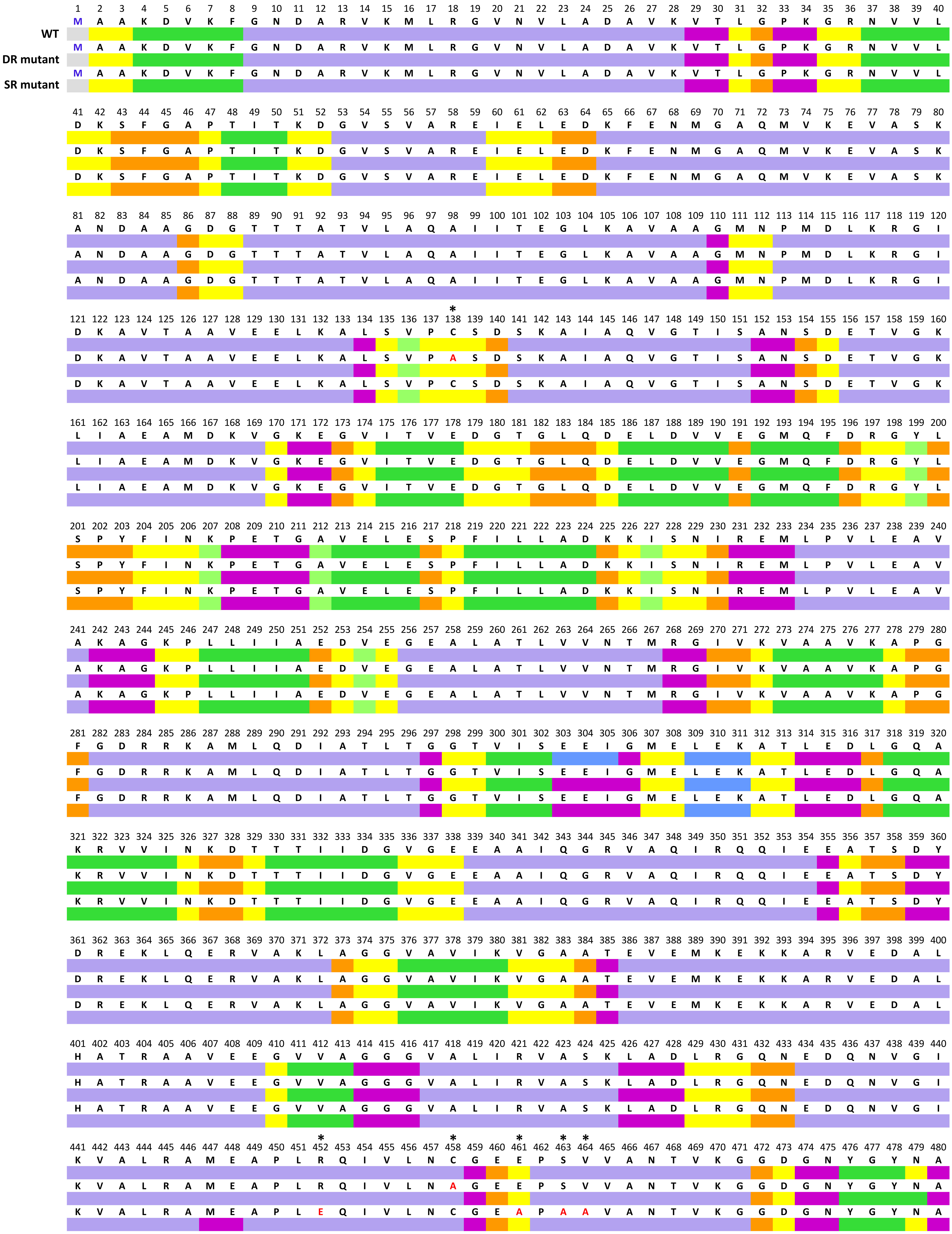
WT
DR mutant
SR mutant
1 2 3 4 5 6 7 8 9 10 11 12 13 14 15 16 17 18 19 20 21 22 23 24 25 26 27 28 29 30 31 32 33 34 35 36 37 38 39 40
M A A K D V K F G N D A R V K M L R G V N V L A D A V K V T L G P K G R N V V L
M A A K D V K F G N D A R V K M L R G V N V L A D A V K V T L G P K G R N V V L
M A A K D V K F G N D A R V K M L R G V N V L A D A V K V T L G P K G R N V V L
41 42 43 44 45 46 47 48 49 50 51 52 53 54 55 56 57 58 59 60 61 62 63 64 65 66 67 68 69 70 71 72 73 74 75 76 77 78 79 80
D K S F G A P T I T K D G V S V A R E I E L E D K F E N M G A Q M V K E V A S K
D K S F G A P T I T K D G V S V A R E I E L E D K F E N M G A Q M V K E V A S K
D K S F G A P T I T K D G V S V A R E I E L E D K F E N M G A Q M V K E V A S K
81 82 83 84 85 86 87 88 89 90 91 92 93 94 95 96 97 98 99 100 101 102 103 104 105 106 107 108 109 110 111 112 113 114 115 116 117 118 119 120
A N D A A G D G T T T A T V L A Q A I I T E G L K A V A A G M N P M D L K R G I
A N D A A G D G T T T A T V L A Q A I I T E G L K A V A A G M N P M D L K R G I
A N D A A G D G T T T A T V L A Q A I I T E G L K A V A A G M N P M D L K R G I
*
121 122 123 124 125 126 127 128 129 130 131 132 133 134 135 136 137 138 139 140 141 142 143 144 145 146 147 148 149 150 151 152 153 154 155 156 157 158 159 160
D K A V T A A V E E L K A L S V P C S D S K A I A Q V G T I S A N S D E T V G K
D K A V T A A V E E L K A L S V P A S D S K A I A Q V G T I S A N S D E T V G K
D K A V T A A V E E L K A L S V P C S D S K A I A Q V G T I S A N S D E T V G K
161 162 163 164 165 166 167 168 169 170 171 172 173 174 175 176 177 178 179 180 181 182 183 184 185 186 187 188 189 190 191 192 193 194 195 196 197 198 199 200
L I A E A M D K V G K E G V I T V E D G T G L Q D E L D V V E G M Q F D R G Y L
L I A E A M D K V G K E G V I T V E D G T G L Q D E L D V V E G M Q F D R G Y L
L I A E A M D K V G K E G V I T V E D G T G L Q D E L D V V E G M Q F D R G Y L
201 202 203 204 205 206 207 208 209 210 211 212 213 214 215 216 217 218 219 220 221 222 223 224 225 226 227 228 229 230 231 232 233 234 235 236 237 238 239 240
S P Y F I N K P E T G A V E L E S P F I L L A D K K I S N I R E M L P V L E A V
S P Y F I N K P E T G A V E L E S P F I L L A D K K I S N I R E M L P V L E A V
S P Y F I N K P E T G A V E L E S P F I L L A D K K I S N I R E M L P V L E A V
241 242 243 244 245 246 247 248 249 250 251 252 253 254 255 256 257 258 259 260 261 262 263 264 265 266 267 268 269 270 271 272 273 274 275 276 277 278 279 280
A K A G K P L L I I A E D V E G E A L A T L V V N T M R G I V K V A A V K A P G
A K A G K P L L I I A E D V E G E A L A T L V V N T M R G I V K V A A V K A P G
A K A G K P L L I I A E D V E G E A L A T L V V N T M R G I V K V A A V K A P G
281 282 283 284 285 286 287 288 289 290 291 292 293 294 295 296 297 298 299 300 301 302 303 304 305 306 307 308 309 310 311 312 313 314 315 316 317 318 319 320
F G D R R K A M L Q D I A T L T G G T V I S E E I G M E L E K A T L E D L G Q A
F G D R R K A M L Q D I A T L T G G T V I S E E I G M E L E K A T L E D L G Q A
F G D R R K A M L Q D I A T L T G G T V I S E E I G M E L E K A T L E D L G Q A
321 322 323 324 325 326 327 328 329 330 331 332 333 334 335 336 337 338 339 340 341 342 343 344 345 346 347 348 349 350 351 352 353 354 355 356 357 358 359 360
K R V V I N K D T T T I I D G V G E E A A I Q G R V A Q I R Q Q I E E A T S D Y
K R V V I N K D T T T I I D G V G E E A A I Q G R V A Q I R Q Q I E E A T S D Y
K R V V I N K D T T T I I D G V G E E A A I Q G R V A Q I R Q Q I E E A T S D Y
361 362 363 364 365 366 367 368 369 370 371 372 373 374 375 376 377 378 379 380 381 382 383 384 385 386 387 388 389 390 391 392 393 394 395 396 397 398 399 400
D R E K L Q E R V A K L A G G V A V I K V G A A T E V E M K E K K A R V E D A L
D R E K L Q E R V A K L A G G V A V I K V G A A T E V E M K E K K A R V E D A L
D R E K L Q E R V A K L A G G V A V I K V G A A T E V E M K E K K A R V E D A L
401 402 403 404 405 406 407 408 409 410 411 412 413 414 415 416 417 418 419 420 421 422 423 424 425 426 427 428 429 430 431 432 433 434 435 436 437 438 439 440
H A T R A A V E E G V V A G G G V A L I R V A S K L A D L R G Q N E D Q N V G I
H A T R A A V E E G V V A G G G V A L I R V A S K L A D L R G Q N E D Q N V G I
H A T R A A V E E G V V A G G G V A L I R V A S K L A D L R G Q N E D Q N V G I
* * * * *
441 442 443 444 445 446 447 448 449 450 451 452 453 454 455 456 457 458 459 460 461 462 463 464 465 466 467 468 469 470 471 472 473 474 475 476 477 478 479 480
K V A L R A M E A P L R Q I V L N C G E E P S V A N T V K G G D G N Y G Y N A
K V A L R A M E A P L R Q I V L N A G E E P S V A N T V K G G D G N Y G Y N A
K V A L R A M E A P L E Q I V L N C G E A P A A V A N T V K G G D G N Y G Y N A

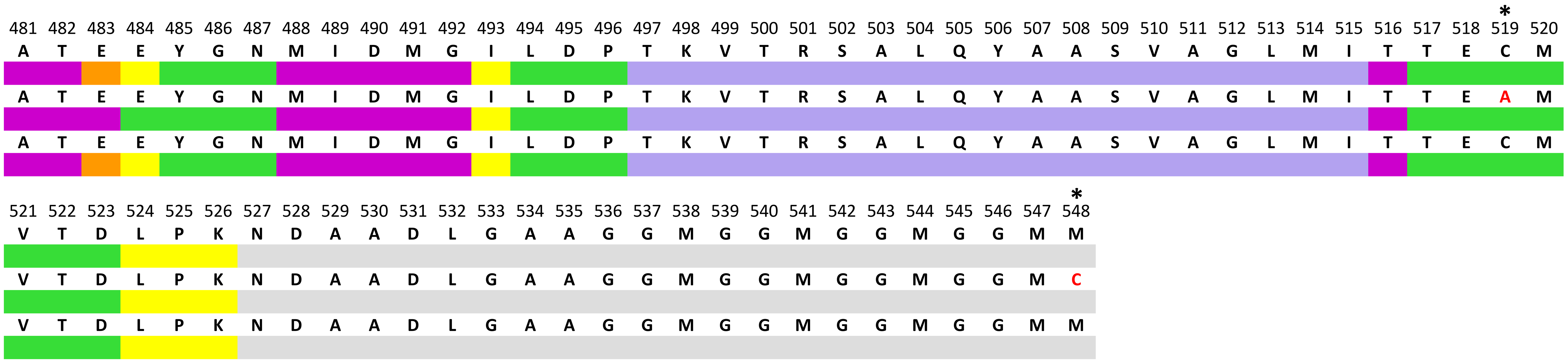

Figure S17: Color-coded motif-scale bars that fit the amino acid residues to their corresponding structural motif in the chain J of the **WT and the two mutated GroEL variants**. Light grey, unresolved region; light blue, α-helix; skyblue, 3_{10}-helix; green, extended strand participating in β-ladder; lime, one residue in isolated β-bridge; orange, bend; yellow, loop; purple, hydrogen-bonded turn (see Experimental Section). The amino acid sequences of the WT $GroEL_{14}$, DR mutant $GroEL_{14}$, and SR mutant $GroEL_7$ correspond to the first, second, and third rows of the sequence alignment, respectively. The amino acid residues that were mutated in the DR mutant $GroEL_{14}$ (C138A, C458A, C519A, and M548C) and in the SR mutant $GroEL_7$ (R452E, E461A, S463A, and V464A) are indicated with asterisks.

A)

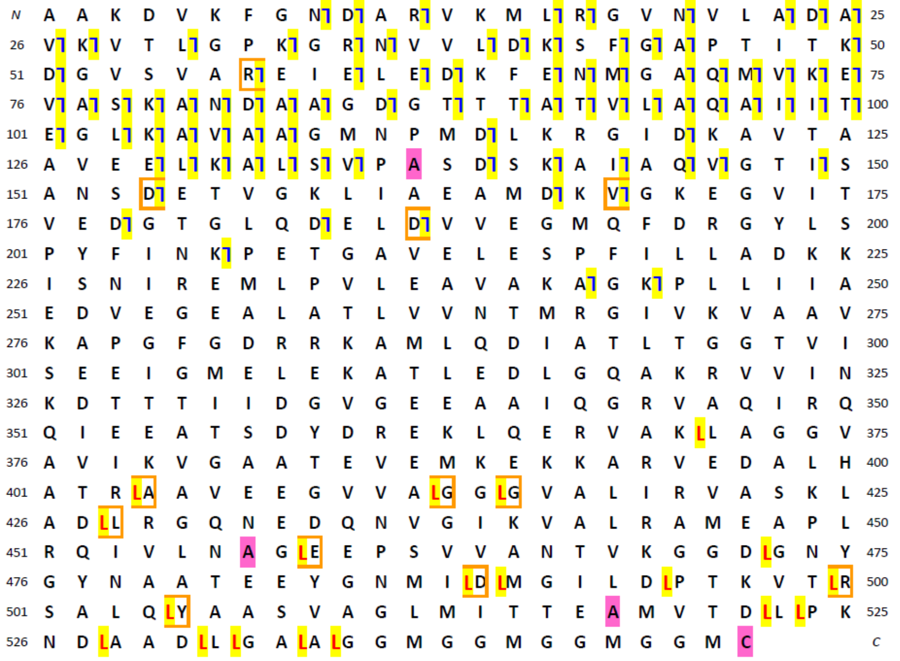

B)

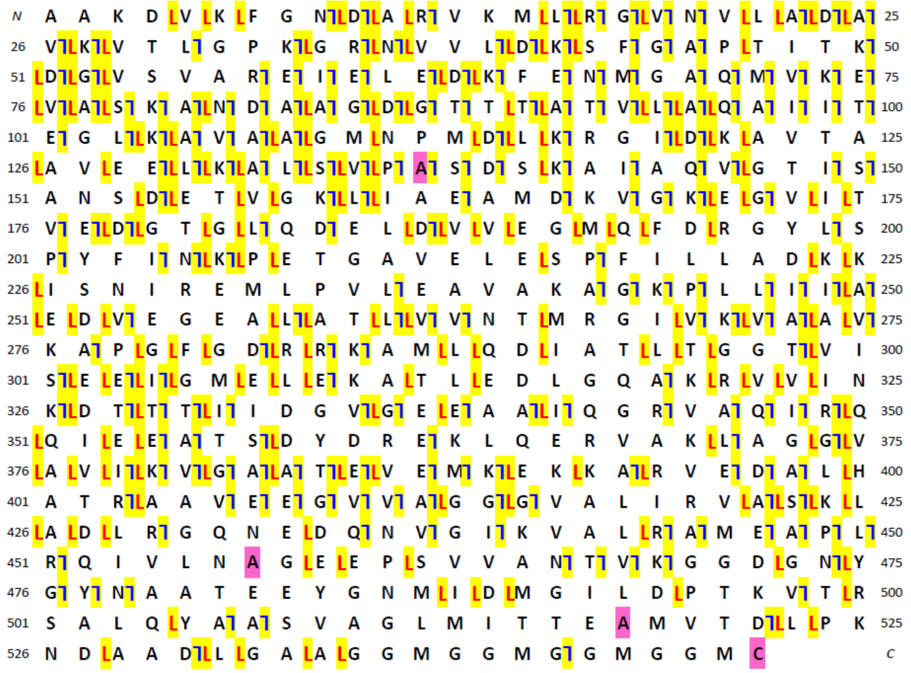

Figure S18: Native CE-pseudo-MS^3 analysis of mutant DR GroEL$_{14}$. Fragmentation maps of (A) *b* and *y* terminal fragment ions (the twelve fragmentations encircled in orange correspond to fragmentations specific to the DR mutant GroEL$_{14}$ that were not detected in the CE-pseudo-MS^3 analysis of WT GroEL$_{14}$), and B) *b* and *y* terminal and *by* internal fragment ions generated in the CE-pseudo-MS^3 analysis of mutant DR GroEL$_{14}$. The inclusion of internal fragments increased the sequence coverage from 20% to 57%.

A)

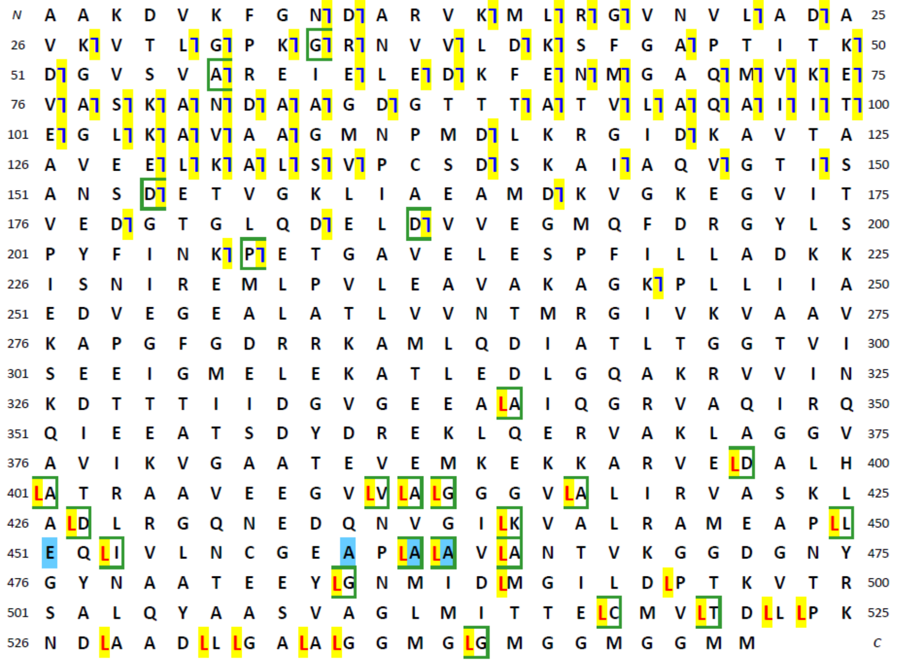

B)

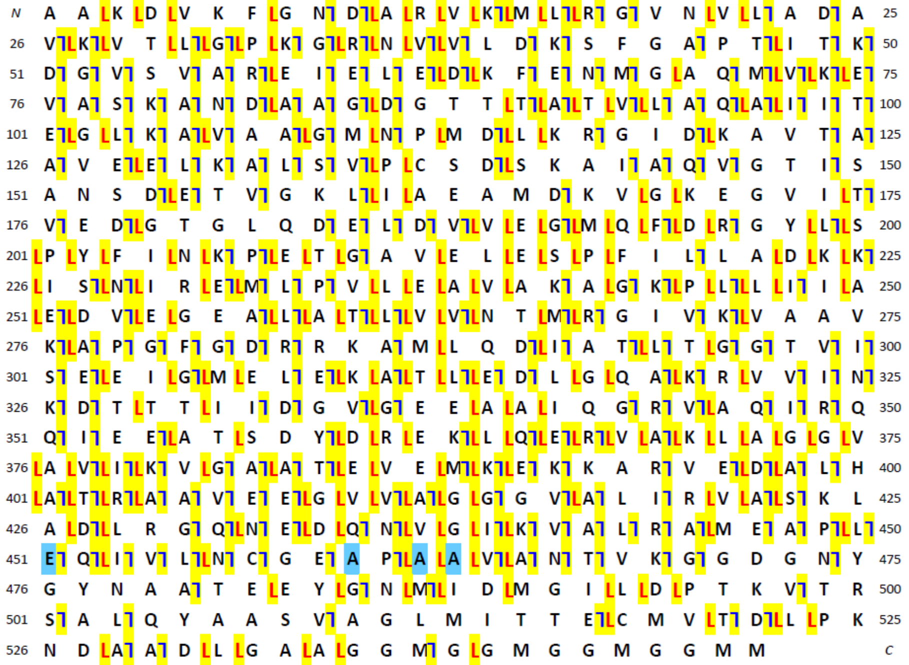

Figure S19: Native CE-pseudo-MS^3 analysis of mutant SR GroEL$_7$. Fragmentation maps of (A) *b* and *y* terminal fragment ions (the twenty-three fragmentations encircled in green correspond to fragmentations specific to the SR mutant GroEL$_7$ that were not detected in the CE-pseudo-MS^3 analysis of WT GroEL$_{14}$), and B) *b* and *y* terminal and *by* internal fragment ions generated in the CE-pseudo-MS^3 analysis of mutant SR GroEL$_7$. The inclusion of internal fragments increased the sequence coverage from 19% to 70%.

Protein complex	Mr_{exp} (Da)	Mr_{th} (Da)	ΔMr (Da)	ΔMr (ppm)
WT $GroEL_{14}$	801238.19	800767.13	471.06	588.26
Mutant $GroEL_{14}$	799488.75	799027.77	460.98	576.93
Mutant $GroEL_{7}$	399629.94	399479.45	150.50	376.74
$GroES_{7}$	72711.48	72708.65	2.83	38.92
$NanA_{4}$	129908.76	129849.09	59.67	459.55
$GroEL_{14}$-$NanA_{1}$	832159.89	831490.04	669.84	805.60
$GroEL_{14}$-$NanA_{2}$	864879.36	863952.31	927.04	1073.03
$GroEL_{14}$-$DapA_{1}$	833079.12	830297.74	2781.38	3349.86
$GroEL_{14}$-$DapA_{2}$	866664.56	861567.71	5096.85	5915.78
$GroEL_{14}$-$(ATP\gamma S)_{2}$	800683.54	800201.08	482.46	602.93
$GroEL_{14}$-$(ATP\gamma S)_{4}$	801711.93	801374.38	337.55	421.21
$GroEL_{14}$-$(ATP\gamma S)_{14}$	807942.65	807240.92	701.74	869.30
$GroEL_{14}$-$(ATP\gamma S)_{7}$-$(BeF_{3})_{7}$-$GroES_{7}$	876768.69	876305.05	463.64	529.09
$GroEL_{14}$-$(ADP)_{3}$	801047.32	800721.94	325.38	406.36
$GroEL_{14}$-$(ADP)_{7}$-$GroES_{7}$	877768.03	875689.48	2078.55	2373.61
$GroEL_{14}$-$(ADP)_{3}$-$(AlF_{3})_{3}$	801289.26	800973.87	315.39	393.76
$GroEL_{14}$-$(ADP)_{7}$-$(AlF_{3})_{7}$-$GroES_{7}$	877954.45	876277.32	1677.13	1913.93

Table S1: Experimental (Mr_{exp}) (n=3) and theoretical (Mr_{th}) molecular masses of the GroEL (wild-type and mutants), GroES, NanA, mutant GroEL-substrate protein, mutant GroEL-nucleotide, and mutant GroEL-GroES oligomeric complexes, as determined in CE-MS analyses of the protein complexes acquired at a MS resolution of 3,125. Possible post-translational modifications and adducts were not accounted for in these calculations. For the mutant GroEL-nucleotide and mutant GroEL-GroES complexes, Mg^{2+} and K^{+} ions were included in the determination of the Mr_{th} (see Methods section).
